# Discovering neutralizing antibodies targeting the stem epitope of H1N1 influenza hemagglutinin with synthetic phage-displayed antibody libraries

**DOI:** 10.1038/srep15053

**Published:** 2015-10-12

**Authors:** Chao-Ping Tung, Ing-Chien Chen, Chung-Ming Yu, Hung-Pin Peng, Jhih-Wei Jian, Shiou-Hwa Ma, Yu-Ching Lee, Jia-Tsrong Jan, An-Suei Yang

**Affiliations:** 1 Genomics Research Center, Academia Sinica, Taipei, Taiwan 115; 2 Institute of Biomedical Informatics, National Yang-Ming University, Taipei, Taiwan 112; 3 Bioinformatics Program, Taiwan International Graduate Program, Institute of Information Science, Academia Sinica, Taipei, Taiwan 115

## Abstract

Broadly neutralizing antibodies developed from the IGHV1–69 germline gene are known to bind to the stem region of hemagglutinin in diverse influenza viruses but the sequence determinants for the antigen recognition, including neutralization potency and binding affinity, are not clearly understood. Such understanding could inform designs of synthetic antibody libraries targeting the stem epitope on hemagglutinin, leading to artificially designed antibodies that are functionally advantageous over antibodies from natural antibody repertoires. In this work, the sequence space of the complementarity determining regions of a broadly neutralizing antibody (F10) targeting the stem epitope on the hemagglutinin of a strain of H1N1 influenza virus was systematically explored; the elucidated antibody-hemagglutinin recognition principles were used to design a phage-displayed antibody library, which was then used to discover neutralizing antibodies against another strain of H1N1 virus. More than 1000 functional antibody candidates were selected from the antibody library and were shown to neutralize the corresponding strain of influenza virus with up to 7 folds higher potency comparing with the parent F10 antibody. The antibody library could be used to discover functionally effective antibodies against other H1N1 influenza viruses, supporting the notion that target-specific antibody libraries can be designed and constructed with systematic sequence-function information.

A large portion of the stem-specific antibodies (40% out of 197 stem-specific antibodies as shown in Pappas *et al.*^1^) bind to the hemagglutinin (HA) of influenza virus through the VH domain originated from human IGHV1–69 germline gene with only a few somatic mutations. B-cell receptors encoded with the germline VH sequence in IgM form can be activated by HA and the affinity maturation of the precursor IgG requires only 2 mutations in the complementarity determining region H1 (CDR-H1) and 5 mutations in the framework region 3 (FR3) to restore activity of a fully matured broadly neutralizing antibody, or bnAb^2^. Phage display selection of bnAbs specifically targeting the stem region of HA from a semi-synthetic antibody library overwhelmingly biased towards the germline IGHV1–69 sequence reveals that in addition to the two key germline-encoded residues in CDR-H2 (Ile53 and Phe54; antibody residue numbers are shown in Kabat number throughout this work) and Tyr98 in CDR-H3, a distinctive Ile52Ser mutation in the CDR-H2 and one additional mutation in the CDR-H1 can restore hetero-subtypic neutralizing activity from a non-active precursor IgG encoded with germline IGHV1–69 sequence, suggesting that IGHV1–69-bnAbs can be efficiently matured from IGHV1–69-encoded B-cell receptors^3^. Recent analysis of the developmental pathways of anti-stem IGHV1–69-bnAbs from a single donor confirms the key roles played by Phe54 and Tyr98 in the initial development of most IGHV1–69-bnAbs^1^. But most strikingly, as few as 2 ~ 3 additional CDR-H1 and CDR-H2 mutations (Ser30Arg – Pro52AAla, or Thr28Pro – Ser30Ile – Ile53Val) in the IGHV1–69-encoded precursor IgG enable hetero-subtypic neutralizing activity, and moreover, additional favorable mutations can substitute the key roles of the aromatic side chains of Phe54 and Tyr98, suggesting that the affinity maturation pathways of the IGHV1–69-bnAbs could be redundant^1^. All these results support the speculation that IGHV1–69 germline gene has been optimized by Darwinian evolution co-existed with the ubiquitous influenza virus infections in humans^2^.

Structures of IGHV1–69-bnAbs in complex with HAs reveal a highly conserved antibody-antigen interaction interface. The antibody VH domain binds to the highly conserved patch on the stem region of HA mainly through the germline-encoded Phe54 in CDR-H2 in connection with the adjacent interaction involving Tyr98 in CDR-H3; CDR-H1 interacts with the stem region of HA through diverse configurations and the antibody VL domain does not make notable contact with the HA in the complex structures. CR6261 in complex with SC1918/H1 HA or Viet04/H5 HA interacts with two patches of epitope on HA: the membrane-distal patch composed of hydrophobic side chains from C-terminal end of helix A in HA2 and adjacent hydrophobic side chains from N- and C-terminal ends of HA1 interacts with Pro28 and Phe29 in CDR-H1 and Phe74 in FR3; the membrane-proximal patch composed of highly conserved Gly20, Trp21, Tyr22, and Ile/Val45 from HA2 with adjacent conserved His18 and His38 (conserved in group 1 HA) from HA1 interacts with IGHV1–69-encoded Ile53 and Phe54 in CDR-H2 and Tyr98 in CDR-H3 (HA residue numbers throughout this work are based on the numbering of the HA structure in 3GBN^4^). The CDR-H1 adapts a non-canonical loop structure to make the side chain of Phe29 accessible for antigen binding even in the absence of the antigen^2^. These interactions enable CR6261 – a bnAb of the group 1 type A influenza viruses – neutralizing viral infection by inhibiting fusion of the viral membrane with that of the host cell^4^. CR9114 – a IGHV1–69-bnAb of both type A and B influenza viruses – recognizes the highly conserved membrane-proximal patch of the epitope on HA with CDR-H2 loop and Tyr98 in CDR-H3 in a complex structure mostly identical to that of CR6261^5^, but the membrane-distal hydrophobic patch of the epitope interacts mostly with Phe74 in FR3 in the absence of the CR6261-like non-polar side chains in CDR-H1, which adapts the conventional type 1 canonical structure^5^. F10 – another IGHV1–69-bnAb of the group 1 type A influenza viruses – also recognizes the common IGHV1–69-bnAb epitope with similar CDR-H1~3 loop conformations: while the highly conserved membrane-proximal epitope patch is recognized with the paratope configuration similar to that of CR6261, the membrane-distal epitope patch is recognized with CDR-H1 loop adapting the type 1 canonical structure where the germline-encoded Phe29 is not accessible for antigen-binding and with the key aromatic side chain of Phe74 in FR3 replaced by small hydrophilic side chain of Ser^6^. Unlike the aromatic interactions between the IGHV1–69 germline-encoded Phe54 with the highly conserved Trp21 of HA2 and His18 and His38 of HA1 common in all the three IGHV1–69-bnAbs, the interactions between the membrane-distal hydrophobic epitope patch and the bnAb CDR-H1/FR3 vary among the complex structures along with diverse CDR-H1 sequences and local conformations. The membrane-distal and membrane-proximal epitopes/paratopes in the complex structures of HA and F10 or CR6261 are shown in Supplementary Figure S1; the membrane-distal epitope and membrane-proximal epitope residue comparisons for 28627 HA sequences of Influenza A from all hosts and subtypes are shown in Supplementary Table 1.

The atomic details of the IGHV1–69-bnAbs in complex with HA and the bnAbs’ maturation pathways during vaccination or infection could lead to better designs of active immunotherapy to elicit universal bnAbs against influenza viruses, but other immunotherapeutic strategies based on antibody therapeutics^7^ and vectored immunoprophylaxis^8^ nevertheless require the bnAb amino acid sequences to be further optimized by exploring more expansive sequence space. Although by definition, bnAbs are able to recognize HA transcending subtypes of influenza virus, it is not expected that a single bnAb sequence could be optimally effective against diverse strains of HA because of the diverse sequences and local conformations of different HAs. It would be more reasonable to anticipate that optimal neutralizing antibodies for corresponding HA strains are to be attained from an antibody library constructed based on bnAb-HA recognition principles. These bnAb-HA recognitions are mediated either by heavy-chain CDR binding to the receptor-binding pocket on the head region of HA^9^ or by heavy-chain CDR binding to the stem region of HA, as described above. While both epitopes are vulnerable sites for broadly neutralizing antibodies, we focused only on the stem epitope of H1N1 HA in this work, because H1 is one of the major influenza virus subtypes and the H1 HA stem region sequences are highly conserved (Supplementary Table 1). Hence, it is reasonable to anticipate that the antibody library developed herein could be applicable to some portion of H1 HA strains.

To elucidate the stem-specific antibody-HA recognition principles, we focused on first optimizing the CDR-H2 sequences of a bnAb against BS/07 H1 HA with a phage-displayed synthetic antibody library, aiming at exhaustively exploring the optimal interactions between the CDR-H2 loop and the highly conserved amino acid cluster on the membrane-proximal epitope of the HA stem. The sequence spaces of the CDR-H1 and CDR-H3 loops were then sequentially explored with additional phage-displayed library designs with the exhaustively optimized CDR residues fixed. The results suggested that the natural IGHV1–69-bnAbs have already been mostly optimized in the CDR-H2 sequences, especially in the CDR-H2 sequence of F10. The critical Tyr98 in CDR-H3 was well-conserved in the optimized CDR-H3 sequences, poised to augment the epitope-recognition by CDR-H2. By contrast, the essential sequence in the CDR-H1 is less well-defined, perhaps due to the flexibility of the loop conformation, ideally to accommodate the less conserved membrane-distal epitope on the HA stem. Together, IGHV1–69-bnAbs mainly recognize the highly conserved epitope patch in the stem region of HA with the largely globally optimized CDR-H2 and the somatic mutations in CDR-H1 can accommodate sequence variation in HA strains.

The findings enabled an antibody library design (named F10-CDRH123) aiming at generating specific neutralizing antibodies targeting the corresponding stem epitope on all strains of H1 HA. To test the antibody library design, we discovered more than 1000 antibody scFv leads from the phage-displayed F10-CDRH123 library panning against another H1 HA: CA/09 H1 HA. All these scFvs bound to the common IGHV1–69-bnAb epitope, and the maximum neutralizing potency was about 3 ~ 7 folds (IC_50_) superior to that of F10 against CA/09 and BS/07 H1N1 influenza viruses. It is thus anticipated that corresponding neutralizing antibodies specific to the stem epitope of many H1 HAs could be developed from the phage-displayed F10-CDRH123 antibody library.

## Results

### Global search of the CDR-H2 sequence of F10-derived binders against a HA revealed sequence preference profiles only compatible with the IGHV1–69*01 gene

The F10 scFv (single chain variable fragment) was used as a model antibody molecule to exhaustively explore the sequence space of CDR-H2 for residues ranging from H52 to H56 – the CDR-H2 region defined by Chothia^10^. The aim was to explore globally optimal CDR-H2 sequences targeting the highly conserved membrane-proximal epitope on HA (Supplementary Figure S1 and Supplementary Table S1). We used F10 scFv as the template because the VH-VL scFv of F10 was successfully expressed both as M13-phage displayed scFv and as phage-free soluble scFv in the supernatant of *E. coli* culture, although the expression of the phage-displayed scFv of CR6261 was known previously^11^. The phage-displayed synthetic antibody library (F10-CDRH2) was constructed with the F10 template (Supplementary Figure S2), where the CDR-H2 region was diversified with degenerate codon NNK (N: A/G/T/C and K: G/T) to cover all 20 natural amino acid types. Oligonucleotide directed mutagenesis was used to construct the phage-displayed F10-CDRH2 library^12^ with complexity >10^9^, which was comparable to the theoretical gene diversity (32^6^) of the antibody library by design.

One caveat of the phage-based neutralizing antibody discovery is to ensure that selected scFv binders relevant to the stem-specific antibody-HA neutralization mechanism could be separated from the antigen with elution buffer for further amplification. The phage display selection procedure was carried out in PBS (pH 7.4) and the bound phage particles were eluted with elution buffer at pH 2.2 (Methods), which is sufficient to shift the electrostatic interactions involving real charges in the antibody-antigen interfaces by removing the charges on aspartic acids and glutamic acids and by adding real charges on histidines on the antibody-antigen complexes^13^,^14^,^15^. As such, most of the bound scFvs on the immobilized antigens should be eluted from the solid substrate, although there is no assurance that all binders could be eluted under this elution condition. The F10 scFv-HA interactions at neutral pH (pH8 to pH6) are abolished at pH lower than 5, where most of the histidines in the antibody-antigen interface are charged, weakening the binding energetics due to real charge electrostatic interactions. This result suggests that the neutralizing potency of F10 does not require sustained binding to HA below pH 5 - it was anticipated that the elution condition was sufficient to select at least a portion of the scFvs with neutralizing potency against HA for further amplification.

Another caveat is to ensure that the selected scFv binders at pH 7.4 retain binding affinity at mild acidic environment in light of the observation that stem-specific neutralizing antibodies bind to HA in the acidic environment of the endosome (pH 5.2 ~ 5.4) to prevent conformational change of the HA^4^. One way to satisfy this criterion would use two consecutive biopanning procedures, one at neutral pH and the other at pH around 5, to isolate scFvs binding to HA in both conditions. Moreover, an ELISA-based procedure would also be needed to screen potential neutralizers with binding affinity to HA in the two pH conditions. Alternatively, to simplify the selection/screening process, we selected the scFv binders at neutral pH and measured the neutralizing capabilities of the selected scFvs, with the goal to confirm the validity of the phage-display selection/amplification cycles during the optimization of the binding affinity and neutralizing potency of the scFvs from the phage-displayed antibody library.

**Figure 1:**
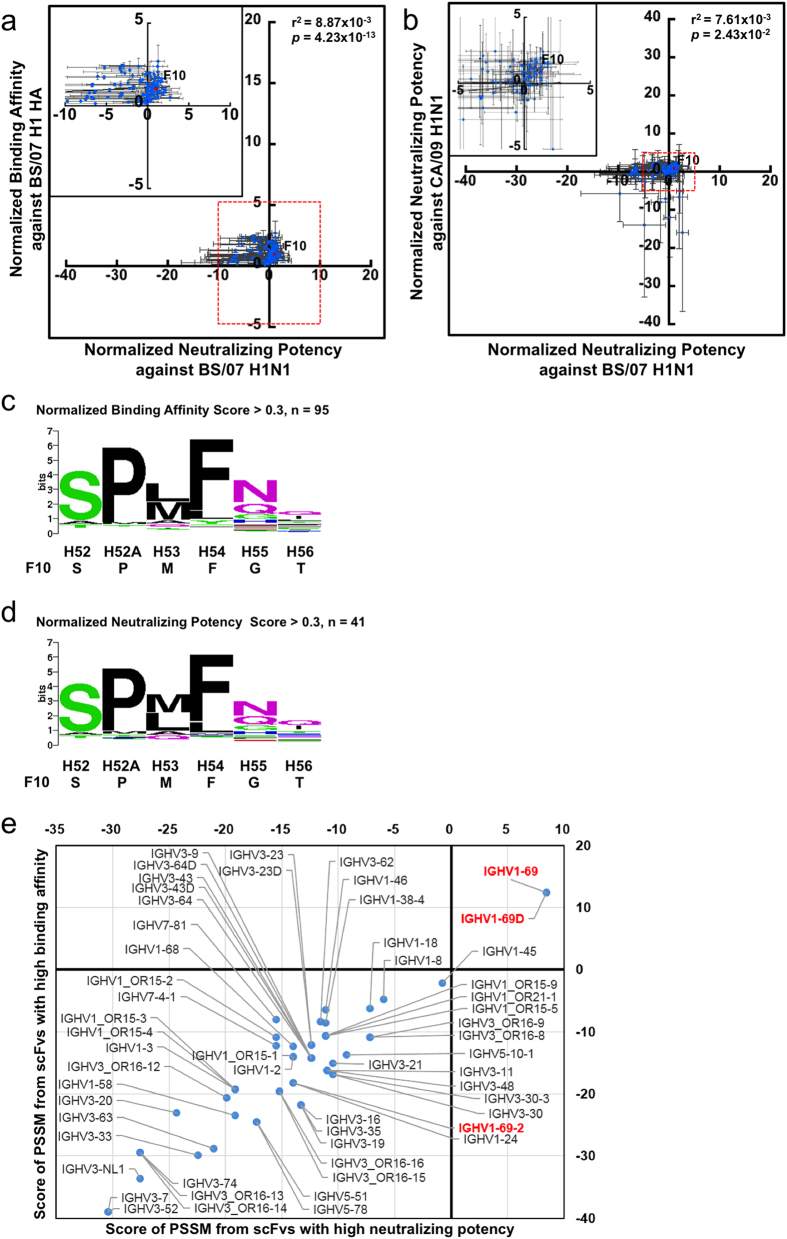
Binding affinities and neutralizing potencies of scFv variants selected from the F10-CDRH2 phage-displayed antibody library. (**a**) The x-axis shows the normalized neutralizing potencies of the 108 selected scFvs from the phage-displayed library against BS/07 H1N1 influenza virus. The y-axis shows the normalized binding affinities of the scFvs to immobilized BS/07 H1 HA. Both scales were normalized to compare with the binding affinity and neutralizing potency of F10 scFv, as shown by the data point colored in red. The error bars were calculated with at least three repeats of each data point measurement. The inset on the left-hand corner shows in enlarged scale of the area enclosed in red dashed square. (**b**) The x-axis shows the normalized neutralizing potencies of the 108 selected scFvs from the phage-displayed library against BS/07 H1N1 influenza virus. The y-axis shows the normalized neutralizing potencies of the selected scFvs against CA/09 H1N1 influenza virus. Both scales were normalized to compare with the neutralizing potency of F10 scFv, as shown by the data point colored in red. The error bars were calculated with at least three repeats of each data point measurement. The inset shows the enlarged scale for the area enclosed in red dashed square. (**c**) The sequence LOGO was calculated from 95 CDR-H2 sequences, which were selected with the threshold of 0.3 in normalized binding affinity against BS/07 H1 HA. (**d**) The sequence LOGO was calculated from 41 CDR-H2 sequences, which were selected with the threshold of 0.3 in normalized neutralizing potency against BS/07 H1N1 virus. The LOGOs in panels (**c**,**d**) were calculated with the background probabilities based on the degenerate codon NNK. (**e**) The x-axis shows the scores of the human germline CDR-H2 sequences calculated with the PSSM constructed with the sequences of scFvs for which the normalized neutralizing potency is greater than 0.3. The y-axis shows the scores of the human germline CDR-H2 sequences calculated with the PSSM constructed with the sequences of scFvs for which the normalized binding affinity is greater than 0.3. The detailed data are summarized in Supplementary Table S2.

The selected phage-displayed scFvs from the selection/amplification cycles were indeed capable of neutralizing the corresponding influenza viruses; the binding affinity and the neutralizing potency of these selected scFvs were nevertheless poorly correlated. Phage-displayed scFvs from the F10-CDRH2 library were selected with 3 rounds of selection-amplification phage display cycle against immobilized recombinant BS/07 H1 HA. The selected scFvs were then expressed as phage-free soluble scFv in the supernatant of the cell culture, which was tested for binding to the recombinant BS/07 H1 HA and with micro-neutralization assay on BS/07 H1N1 (H1N1 A/Brisbane/59/2007) and CA/09 H1N1 (H1N1 A/California/7/2009) viral infection to MDCK cells (Fig. 1a,b). While the maximal binding affinity of the selected scFv variants to HA were a few folds higher than that of F10 scFv (Fig. 1a), the neutralizing potencies of these scFv variants against both BS/07 and CA/09 H1N1 viruses were not clearly superior to that of F10 scFv (Fig. 1a,b). The binding affinities of 108 selected scFv variants from the F10-CDRH2 library had no clear linear correlation (r^2^ < 0.01) with their neutralizing potencies against the corresponding influenza virus (Fig. 1a). The sequences and the binding/neutralization data of the 108 selected scFv variants are summarized in Supplementary Table S2. These results suggested that the F10 CDR-H2 sequence had been mostly optimized for neutralizing both of the H1N1 viruses.

The germline sequence of F10 CDR-H2 is remarkably compatible with the sequence preference profiles of the scFv variants selected for high binding affinity or high neutralizing potency. The sequence profiles of the scFv variants selected for high binding affinity and high neutralizing potency are summarized in Fig. 1c,d respectively. Comparing these sequence profiles with the sequence profile of F10-CDRH2 scFvs randomly selected before selection/amplification cycles (Supplementary Figure S3) indicates that the sequence profiles in Fig. 1c,d reflected the sequence requirements for binding and neutralization. Sequence preference biased due to structural requirements had been ruled out because scFvs selected for folding stability do not have any significant sequence preference in the CDRs^16^. The similarity between the sequence profiles in Fig. 1c,d indicated that the sequence requirements for binding are consistent with the sequence requirements for neutralization. In addition, the consensus sequence (S-P-M/L-F-N-Q) was strikingly similar to F10 CDR-H2 sequence (S-P-M-F-G-T), especially in the H52 ~ H54 residue positions, again suggesting that the sequence of F10 CDR-H2 had largely reached the global neutralizing potency optimum targeting the highly conserved membrane-proximal epitope of IGHV1–69-bnAbs on the HA stem (Supplementary Figure S1). The position specific scoring matrices (PSSMs)^17^ derived from the sequences of the scFv variants for which the sequence LOGOs are shown in Fig. 1c,d were used to calculate the fitness scores of all human germline CDR-H2 sequences (Methods). The CDR-H2 sequence of IGHV1–69 gene appears as the only human germline CDR-H2 sequence that is positively compatible with the PSSMs (Fig. 1e), explaining the observations that the highly conserved epitope patch in the stem region of HA (Supplementary Figure S1 and Supplementary Table S1) can be optimally recognized with the CDR-H2 loop of the IGHV1–69 germline gene with only a few somatic mutations^18^.

### Global search of the CDR-H1 sequence of F10-derived binders against the HA yielded less well-defined sequence preference profiles compatible with many human germline VH genes

The sequence space of CDR-H1 for residue positions ranging from H24 to H32 in the F10 template was explored exhaustively in the F10-CDRH1 library. Two additional residue positions (H24 ~ H25) were simultaneously explored along with the CDR-H1 region (H26 ~ H32) defined by Chothia^10^ because the close proximity of the side chain in H24 to that of H26 in the type 1 canonical structure could influence the local conformation of the CDR-H1 loop. Since the CDR-H2 sequence was known to be optimized in F10 (see above), only the residue positions from H24 to H32 were diversified with the NNK degenerate codon in the F10-CDRH1 library with complexity >10^9^ (Supplementary Figure S2); the sequence profile of randomly selected F10-CDRH1 scFvs did not show any significant biases due to phage library preparation (Supplementary Figure S3). The complexity limit of the F10-CDRH1 library is not enough to cover the theoretical gene diversity (32^9^) of the experimental design. Still, the complexity of 10^9^ is sufficiently applicable to investigate cooperative combinations involving up to any 4 residues in 9 NNK-encoded residue positions (32^4^ × 9!/5!/4!). Given that most local sequence features involve one or two cooperatively dependent residues (as in tight turns and salt bridges), the library complexity should sustain the aim to explore globally optimal CDR-H1 sequences for the HA epitope of IGHV1–69-bnAbs to a large extent.

**Figure 2:**
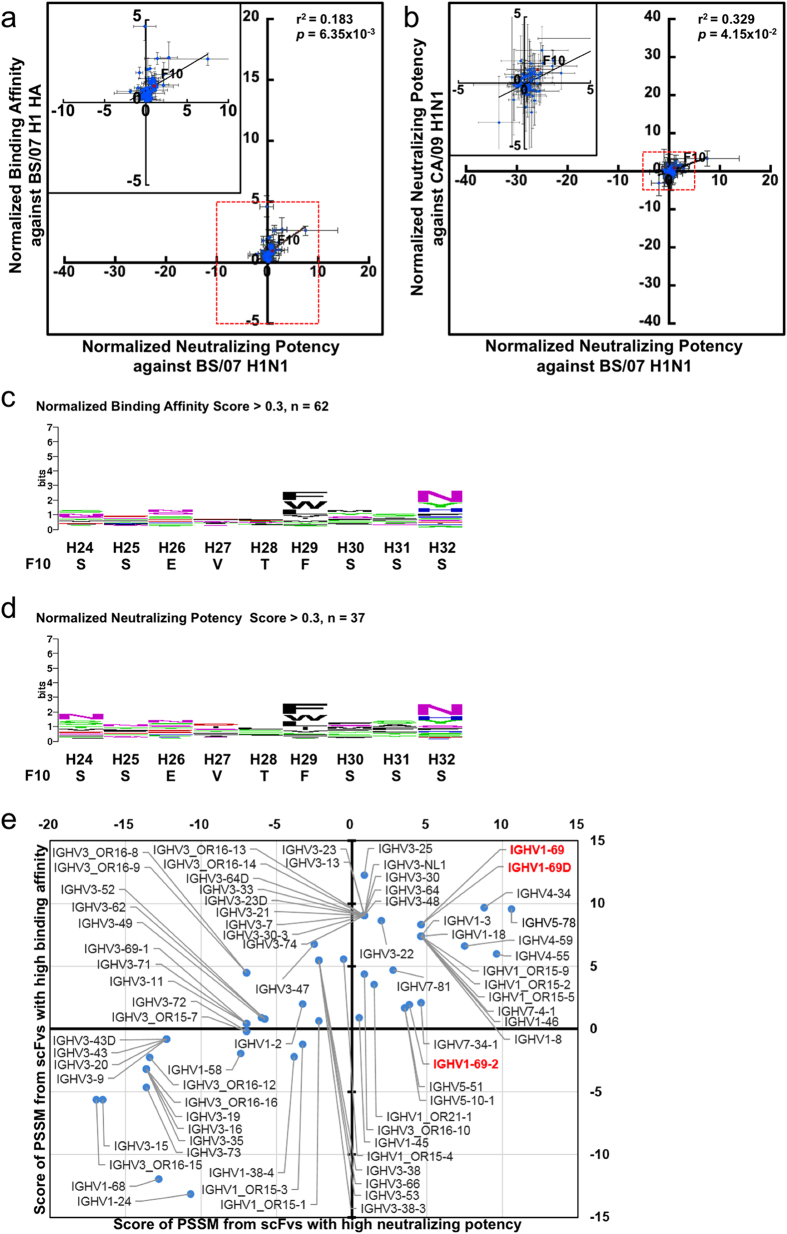
Binding affinities and neutralizing potencies of scFv variants selected from the F10-CDRH1 phage-displayed antibody library. The descriptions of the panels are the same as in Fig. 1. The detailed data for the 78 data points are summarized in Supplementary Table S3.

The maximal binding affinity of the selected scFv variants from the F10-CDRH1 library with immobilized BS/07 H1 HA were a few folds higher than that of F10 scFv (Fig. 2a); the neutralizing potencies of a few scFv variants against both BS/07 and CA/09 H1N1 viruses were slightly superior to that of F10 scFv (Fig. 2a,b). Again, the binding affinity of 78 selected scFv variants from the F10-CDRH1 library had no clear linear correlation (r^2^ < 0.2) with their neutralizing potency against the corresponding influenza virus (Fig. 2a); the correlation of the neutralizing potencies against the two H1N1 influenza viruses were not obvious given the limitation of the experimental accuracy (Fig. 2b). The sequences and the binding/neutralization data of the 78 selected scFv variants are summarized in Supplementary Table S3.

The sequence profiles of the scFv variants selected for high binding affinity and high neutralizing potency, as summarized in Fig. 2c,d respectively, did not shown strong preferences for amino acid types in all the residue positions explored. The most conserved residue position was H29, for which the aromatic/hydrophobic side chain is expected to stabilize the type 1 canonical structure of the CDR-H1 loop by packing with the upper hydrophobic core of the VH domain^16^. The other conserved residue position is H32, where the aromatic/hydrophilic side chain is in contact with the membrane-distal epitope on HA stem. Overall, the similarity between the sequence profiles indicated that the sequence requirements for binding are consistent with the sequence requirements for neutralization, but the lack of sequence preferences in the sequence profiles for CDR-H1 shown in Fig. 2c,d were in sharp contrast to the well-defined sequence preference profiles for CDR-H2 (Fig. 1c,d). The lack of prominent sequence preferences in the CDR-H1 region was unexpected, judging by the extensive intermolecular hydrogen bonding involving the side chains of Thr28, Ser30 and Ser31 and the hydrophobic/aromatic interactions involving the side chains of Val27 and Phe32 in the F10-HA complex structure^6^. The discrepancy could be attributed to that the H5 HA structure in the F10-HA complex structure (Supplementary Figure S1) might not reflect the structural contacts relevant to the interactions between F10 and H1 HA in this work. Nevertheless, the maximal affinity for the scFv variants shown in Fig. 2a indicates that many alternative contacts are equally adequate to provide favorable binding energy in place of those of F10 CDR-H1.

The sequence preference profiles shown in Fig. 2c,d were not as compatible with the CDR-H1 germline sequence of IGHV1–69-bnAbs. As shown in Fig. 2e, many other human germline CDR-H1 sequences were better compatible with the PSSMs derived from the selected scFv variants for which the sequence profiles are shown in Fig. 2c,d. This result is in agreement with the conclusion of the previous paragraph that the F10 CDR-H1 local structure could be quite flexible to accommodate large number of alternative sequences that are equally effective in binding to the corresponding epitope on the HA stem, and as such, the CDR-H1 sequences could be specific to the HA mutations in the corresponding epitope. The flexibility of CDR-H1 is advantageous to accommodate the less conserved membrane-distal patch of epitope in the HA stem (Supplementary Table S1).

### Global search of the CDR-H3 sequence space of F10 revealed the prominent role of Tyr98 and Pro96 in assistance of CDR-H2 recognizing the conserved HA stem epitope

**Figure 3:**
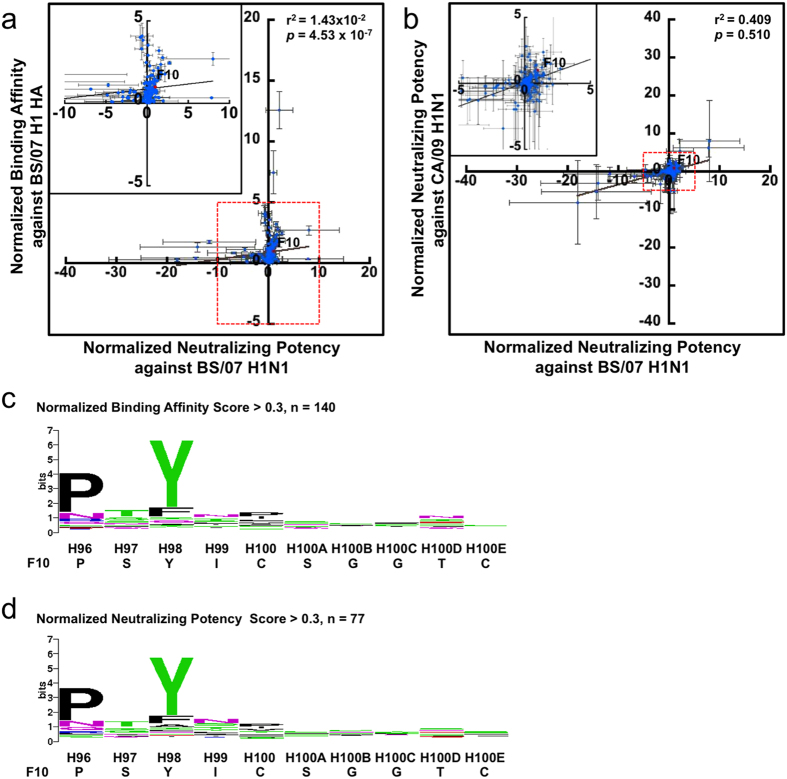
Binding affinities and neutralizing potencies of scFv variants selected from the F10-CDRH3 phage-displayed antibody library. The descriptions of the panels (**a**–**d**) are the same as in Fig. 1a–d, respectively. The detailed data for the 201 data points are summarized in Supplementary Table S4.

The sequence space of CDR-H3 for residue positions ranging from H96 to H100E in the F10 template was explored exhaustively in the F10-CDRH3 library with complexity >10^9^; the sequence profile of randomly selected F10-CDRH3 scFvs did not show any significant biases due to phage library preparation (Supplementary Figure S3). The aim was to explore globally optimal CDR-H3 sequences that are in contact with the HA epitope of IGHV1–69-bnAbs. Again, the maximal binding affinity of the selected scFv variants from the F10-CDRH3 library was a few folds higher than that of F10 scFv (Fig. 3a); the neutralizing potencies of a few scFv variants against both BS/07 and CA/09 H1N1 viruses were only marginally superior to that of F10 scFv (Fig. 3a,b). The binding affinity of 201 selected scFv variants from the F10-CDRH3 library had no clear linear correlation (r^2^ < 0.1) with their neutralizing potency against the corresponding influenza virus (Fig. 3a); the neutralizing potencies against the two influenza viruses were marginally correlated (r^2^ = 0.41) at best considering the experimental error (Fig. 3b). The sequences and the binding/neutralization data of the 201 selected scFv variants are summarized in Supplementary Table S4. The sequence profiles of the scFv variants selected for high binding affinity and high neutralizing potency are summarized in Fig. 3c,d respectively. The conserved residue position Tyr98 aromatic side chain is expected to augment the CDR-H2 recognition of the highly conserved epitope in the HA stem by contacting the aliphatic atoms on Thr41 and Ile45 of HA2^6^ (Supplementary Figure S1). Pro96 was also highly conserved. This residue does not contact the antigen (Supplementary Figure S1) and could support the rigidity of the CDR-H3 loop structure, which in turn sustain the contact position of the side chain of Tyr98. Phage library preparation biases (Supplementary Figure S3) and structural preferences^16^ have been ruled out from accounting for the sequence preferences of Pro96 and Tyr98. The other CDR-H3 positions are not in contact with HA, and thus the preference profiles were not expected to be well-defined. However, it was unexpected that the disulfide bond Cys100-Cys100E in F10 was not conserved, although disulfide bonds in CDR-H3 are frequently required for antigen recognition^19^. The results suggested that the F10 CDR-H3 local structure could be also quite flexible to accommodate alternative sequences, as long as the key residue Tyr98 is in proper contact, perhaps assisted by Pro96, with the corresponding epitope.

### H1 HA-binding scFvs selected from F10-CDRH1~3 libraries bound to the H1 HAs with correlation but did not cross-react to H3 HAs or other human antigens

**Figure 4:**
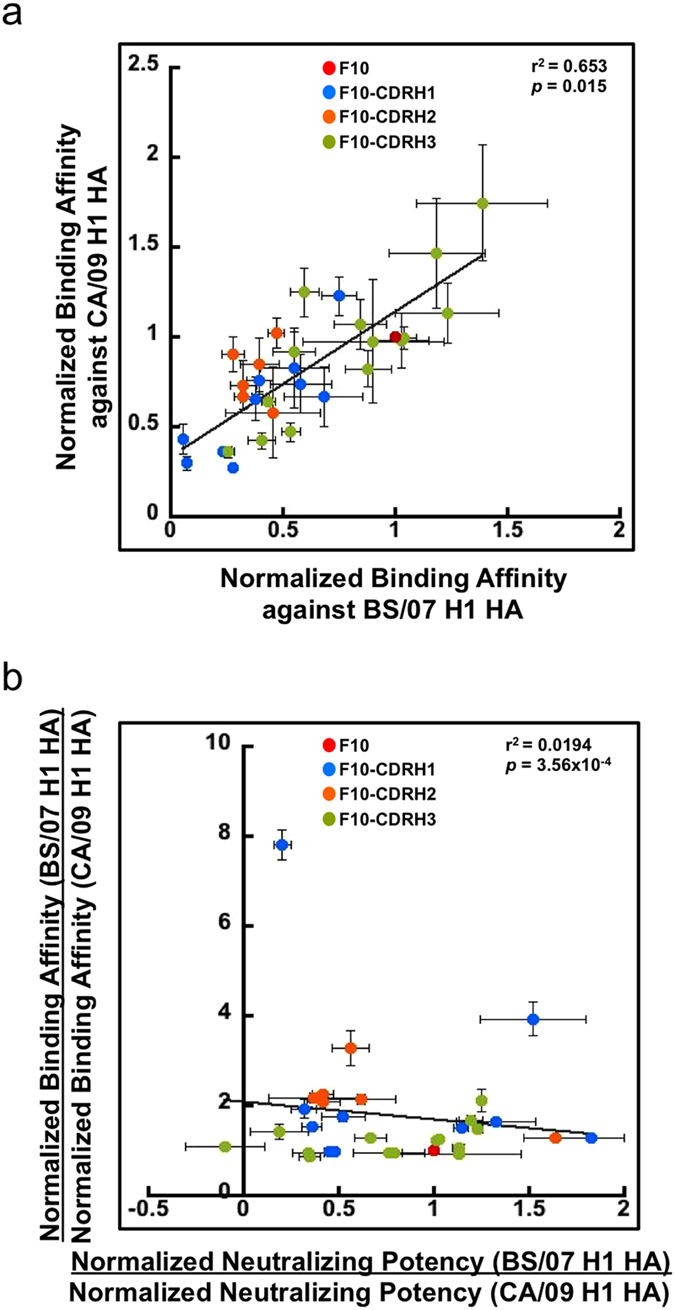
Correlations of binding affinities and neutralizing potencies of scFv variants from F10-CDRH1~3 phage-displayed antibody libraries. (**a**) Normalized binding affinities for 6, 10, 14 randomly selected scFv variants from Figs 1a, 2a and 3a respectively were measured against CA/09 H1 HA (y-axis) and BS/07 H1 HA (x-axis) respectively. (**b**) Ratios of normalized neutralizing potencies are plotted against ratios of normalized binding affinities. In both panels (**a**,**b**), the error bars were calculated with at least three repeats of each data point measurement. Pearson’s correlation coefficient (r) and the p-value (p) of Student’s t-test were calculated with Microsoft Excel.

The normalized binding affinities of scFvs from library F10-CDRH1~3 had strong linear correlation in binding to CA/09 H1 HA and BS/07 H1 HA, but the correlated improvement of affinity against both strains of virus did not necessarily imply that the gain of the neutralizing potency against the two strains of virus are correlated. Figure 4a shows the strong correlation of the normalized binding affinity for randomly selected scFvs from the three synthetic antibody libraries against the two H1 HAs, explaining that the ratios of the normalized binding affinities for the two H1 HAs were centered near 1 ~ 2 (Fig. 4b, y-axis). However, the ratios of the normalized neutralizing potency against the two strains of influenza virus scattered between 0 and 2 (Fig. 4b, x-axis), indicating that the improvement of binding affinity and neutralizing potency of an antibody against one virus strain were likely to lead to improvement of binding affinity to the other strain but not necessarily lead to improvement of neutralizing potency.

**Figure 5:**
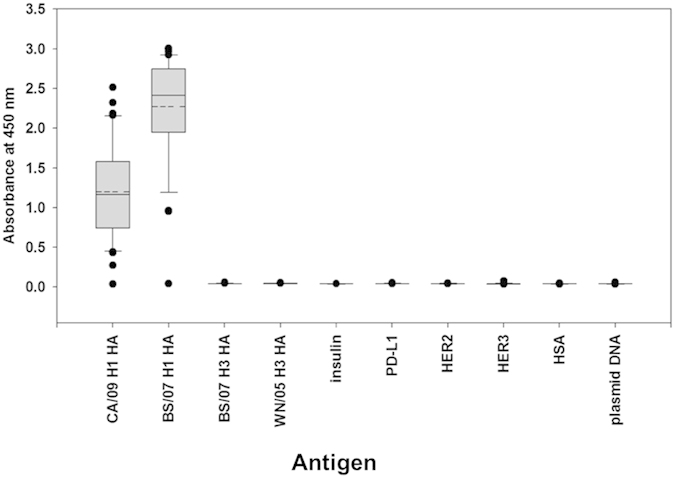
Cross-reactivity of randomly selected scFvs from F10-CDRH1~3 libraries. The x-axis shows the antigens - CA/09 H1 HA: hemagglutinin of A/California/7/2009 (H1N1); BS/07 H1 HA: hemagglutinin of A/Brisbane/59/2007(H1N1); BS/07 H3 HA: hemagglutinin of A/Brisbane/10/2007 (H3N2); WN/05 H3 HA: hemagglutinin of A/Wisconsin/67/2005 (H3N2); Insulin: human insulin; PD-L1: programmed death-ligand 1; HER2: human epidermal growth factor receptor 2; HER3: human epidermal growth factor receptor 3; HSA: human serum albumin; plasmid DNA: pCANTAB5E. The y-axis shows the distributions of the ELISA measurements in OD450 nm for randomly selected 10, 6, and 14 scFvs from Supplementary Table S3, S2 and S4 respectively. The lower boundary of the grey box in a box plot is the 25th percentile. The upper boundary of the box is the 75th percentile. The solid line in the box marks the median. The dash line in the box marks the mean. The whiskers above and below the box indicate the 90th and 10th percentiles. Black circles are outlying points.

The scFvs selected from the F10-CDRH1~3 synthetic antibody libraries were specific only to the H1 HAs. Synthetic antibodies are not negatively selected by immune system and thus could cross-react to irrelevant antigens. To investigate the cross-reactivity for the scFvs selected from the F10-CDRH1~3 scFv libraries, we randomly selected 10, 6, and 14 scFvs from Supplementary Table S3, S2 and S4 respectively and measured the binding of these scFvs to H1 HAs, H3 HAs, other diverse human proteins and DNA (Fig. 5). None of the selected scFvs bound to antigens other than CA/09 H1 HA and BS/07 H1 HA (Fig. 5), suggesting that the selected scFvs from the F10-CDRH1~3 scFv libraries were specific only to H1 HAs. This specificity is in agreement with that of the parent template F10, which only binds to group 1 HAs (such as H1 HAs) but not group 2 HA (such as H3 HAs)^6^. The averaged affinity for the representative scFvs binding to BS/07 H1 HA was higher than that binding to CA/09 H1 HA (Fig. 5) because these scFvs were selected against immobilized BS/07 H1 HA.

### Antibody library designed with information from the antibodies binding to BS/07 H1 HA can be used to discover potent neutralizing antibodies targeting the corresponding stem epitope on CA/09 H1 HA

**Figure 6:**
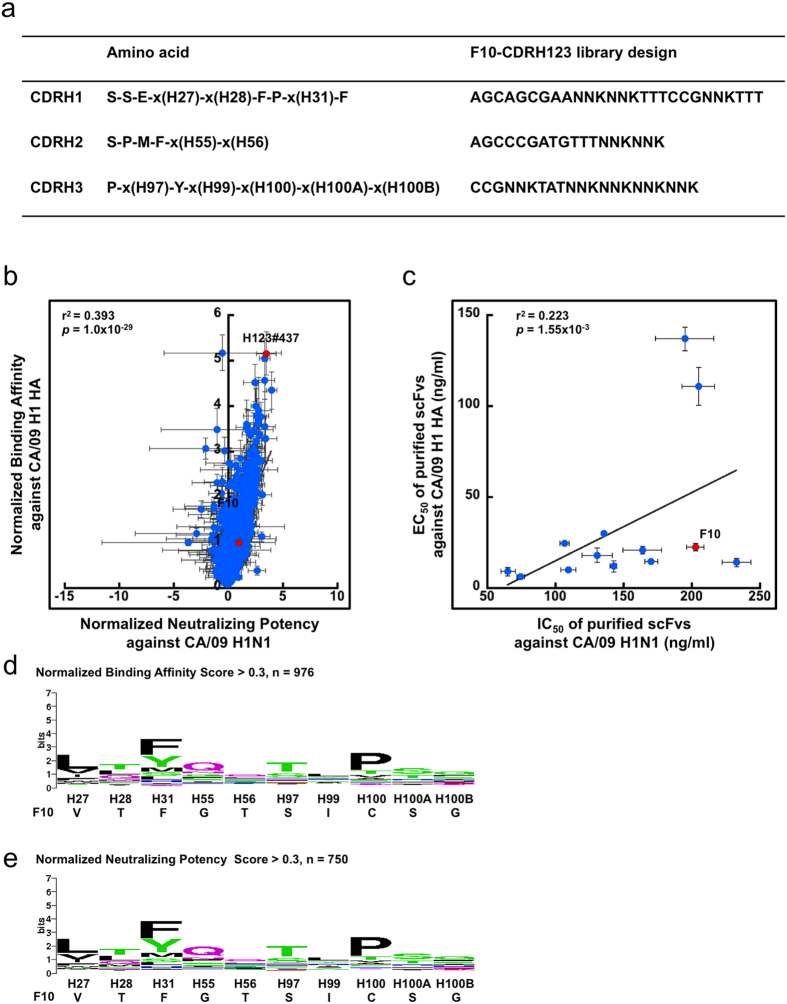
The design and antibody discovery results of F10-CDRH123 phage-displayed antibody library. (**a**) The antibody library design is shown. The rationale is described in the main text. (**b**) The x-axis shows the normalized neutralizing potencies of the 1049 selected scFvs from the phage-displayed library against CA/09 H1N1 influenza virus. The y-axis shows the normalized binding affinities of the scFvs to immobilized CA/09 H1 HA. Both scales were normalized to compare with the binding affinity and neutralizing potency of F10 scFv, as shown by the data point colored in red. (**c**) 12 randomly selected scFvs from panel (**b**) were expressed and purified. The EC_50_’s of these scFvs for binding to CA/09 H1 HA are plotted against the IC_50_’s for neutralizing CA/09 H1N1 virus. In both panels (**b**,**c**), the error bars were calculated with at least three repeats of each data point measurement. Pearson’s correlation coefficient (r) and the p-value (p) of Student’s t-test were calculated with Microsoft Excel. (**d**) The sequence LOGO was calculated from the CDR-H1~H3 sequences with the normalized binding affinity greater than 0.3. (**e**) The sequence LOGO was calculated from the CDR-H1~H3 sequences with the normalized neutralizing potency greater than 0.3. Both LOGOs were calculated with the background probabilities based on the degenerate codon NNK. The detailed data for the 1049 data points are summarized in Supplementary Table S5.

The results above (Figs 1, 2, 3, 4, 5) informed an antibody library design aiming at generating specific and optimal neutralizing antibodies targeting the corresponding stem epitope on diverse strains of H1 HA. Figure 6a shows the F10-CDRH123 antibody library design, where the CDR-H2 residues Ser52-Pro52A-Met53-Phe54 and CDR-H3 residues Pro96 and Tyr98 were fixed for optimal binding to the highly conserved membrane-proximal epitope patch on the HA stem. Met53 and Phe54 make key contacts with the HA epitope (Fig. 1). Ser52 does not contact the antigen, but the hydroxyl side chain forms hydrogen bond with the backbone carboxyl group of Tyr98^6^, likely to stabilize the core paratope structure. Pro52A is encoded in IGHV1–69 gene and could be responsible for the rigidity of the CDR-H2 loop structure. Residue position Phe29 was fixed to stabilize the CDR-H1 type 1 canonical structure; Pro30 was fixed to interact with the largely hydrophobic membrane-distal epitope patch on the HA stem and also to reduce the conformational entropy penalty of the CDR-H1 loop upon binding to the epitope; F32 was fixed to aid the possible hydrophobic interactions with hydrophobic residues in the membrane-distal epitopes of H1 HAs. The CDR-H1 residue positions H27, H28, H31 are expected to contact with the less conserved membrane-distal epitope and thus are diversified with degenerate codon NNK. The other unfixed residue positions (marked ‘x’ in Fig. 6a) were also diversified because of their vicinity to the fixed residue positions and their likelihood to interact, albeit marginally, with the HA stem epitope. In addition, the CDR-H3 sequence length in the F10-CDRH123 library was fixed at 12 residues as in CR6261 and CR9114 without the disulfide bond as in F10 (Supplementary Figure S1). The disulfide bond was not expected to be critical for antigen recognition, as revealed in the results above (Fig. 3c,d). The aim of the F10-CDRH123 library was to explore globally optimal sequences targeting the epitope of IGHV1–69-bnAbs in diverse strains of HA.

The phage-displayed F10-CDRH123 antibody library was constructed (Supplementary Figure S2) to the complexity >10^9^ without significant biases due to phage library preparation (Supplementary Figure S3). The library was tested for neutralizing antibody discovery against CA/09 H1N1 influenza virus. The experiment was designed to test the rationale that a library designed with the information arrived with one strain of H1 HA (BS/07, Figs 1, 2, 3) is equally applicable of discovering optimal binder and neutralizer against another strain of H1 HA (CA/09 in this case). More than 1000 scFvs binding to immobilized CA/09 H1 HA were discovered after three rounds of phage display selection-amplification cycle. All these selected scFvs competed with F10 scFv for binding to HA, indicating that all these scFvs bound to the common epitope of IGHV1–69-bnAbs. The binding affinities of a large portion of the selected scFvs were a few folds higher than that of F10 scFv (Fig. 6b). Unlike the results from the F10-CDRH1~3 libraries (Figs 1a, 2a, 3a and 4b), the binding affinities of the scFv variants from the F10-CDRH123 library were overall correlated to an extent (r^2^ = 0.39, p-value = 10^−29^) with their neutralizing potencies against CA/09 H1N1 influenza virus (Fig. 6b), suggesting that the conformational uncertainty could be partially alleviated by the constant residues in the key CDR positions. By contrast to the marginal improvement in neutralizing potencies shown in Figs 1, 2, 3, the neutralizing potencies of a large portion of the selected scFv variants against CA/09 H1N1 virus were superior to that of F10 scFv (Fig. 6b). These results indicated that simultaneous diversification of the residues in all CDRs are necessary to cooperatively optimize the binding affinity and neutralizing potency.

The measurements of normalized binding affinity and normalized neutralizing potency can be carried out in microtiter plate format so as to generate large amount of data as shown in Figs 1, 2, 3, 4, 5. But the data sets came with large experimental uncertainties, as indicated in the error bars in Fig. 6b. An alternative is to measure the EC_50_ and IC_50_ for the purified scFvs, as shown in Fig. 6c. The EC_50_ and IC_50_ for a tiny portion of the scFvs were measured (Fig. 6c), because the expression and purification of the scFvs (Supplementary Methods) and the measurements of EC_50_ and IC_50_ (Supplementary Methods) are labor- and resource-intensive. Nevertheless, the EC_50_ for CA/09 H1 HA and IC_50_ for CA/09 H1N1 virus for randomly selected scFvs were marginally correlated to an extent (Fig. 6c and Supplementary Table S6), although the correlation is less significant as that shown in Fig. 6b, likely due to the limited data volume in Fig. 6c.

The sequence profiles of the selected scFv variants are summarized in Fig. 6d,e. The sequences and the binding/neutralization data of 1049 selected scFv variants are summarized in Supplementary Table S5. The moderately conserved hydrophobic residues in H27 and H31 were expected due to the hydrophobic interactions with the membrane-distal hydrophobic epitope patch. The preference of Pro at H100 is consistent with similar findings previously^3^. Similar to the results shown in Figs 1, 2, 3, the sequence preference profiles for the other CDR positions in Fig. 6d,e were only moderately conserved at best, confirming that the amino acid types of these positions are not critically restricted for the antigen recognition.

**Figure 7:**
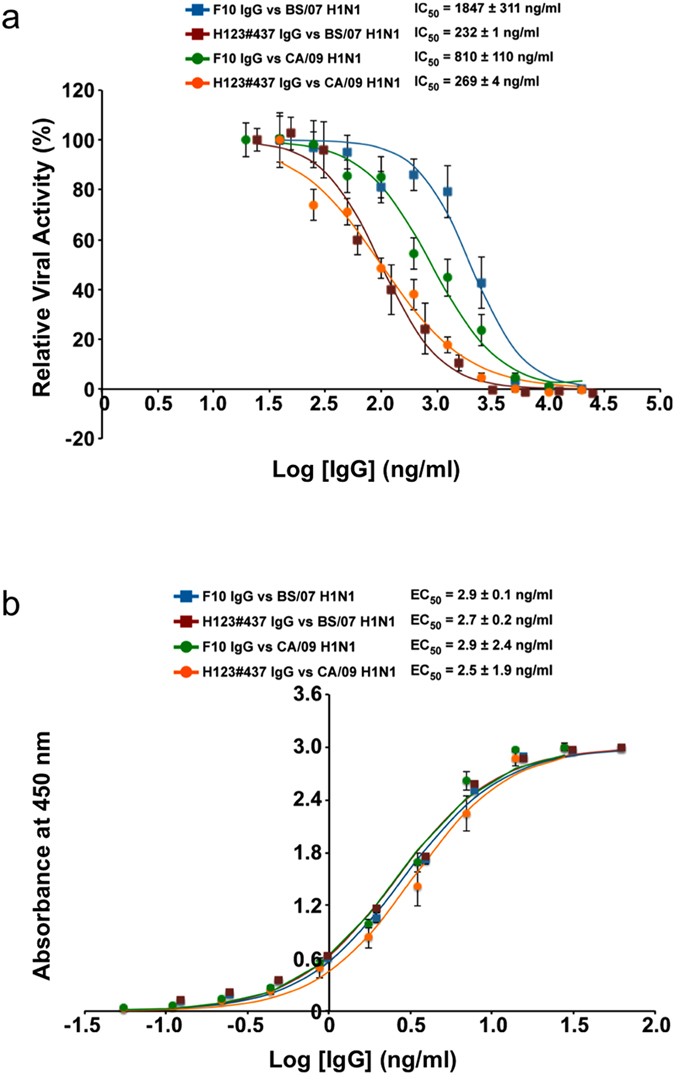
Comparisons of neutralizing potencies and binding affinities of F10 IgG and H123#437 IgG against two strains of H1N1 virus. (**a**) The x-axis shows the concentration of IgG in logarithm scale and the y-axis shows the percentage of neutralization. Each neutralization data point was measured in triplicate to calculate the error bar. The IC_50_’s and standard deviations were calculated with three independent measurements. (**b**) The x-axis shows the concentration of IgG in logarithm scale and the y-axis shows the OD450 nm value from ELISA measurement. Each binding data point was measured in triplicate to calculate the error bar. The EC_50_’s and standard deviations were calculated with three independent measurements.

IgG constructed based on the best of the selected scFv variants from F10-CDRH123 was demonstrated to gain neutralizing potency against the influenza virus infection by 3 ~ 7 folds depending on the H1N1 virus strains. H123#437 scFv (highlighted in red in Supplementary Table S5 and Fig. 6b) had the highest normalized binding affinity (5.2 ± 0.5) and second highest normalized neutralizing potency (3.5 ± 0.9) among the selected scFvs. The H123#437 IgG constructed based on the H123#437 scFv neutralized CA/09 H1N1 infection with IC_50_ of 269 ± 4 ng/ml, which was about 3 folds more potent than that of the F10 IgG (IC_50_ = 810 ± 110 ng/ml) against the infection of the same H1N1 virus (Fig. 7a), confirming the anticipated improvement in neutralizing potency of the H123#437 scFv. Moreover, the H123#437 IgG neutralized BS/07 H1N1 infection with IC_50_ of 232 ± 1 ng/ml, which was about 7 folds more potent than that of the F10 IgG (IC_50_ = 1847 ± 311 ng/ml) against the infection of the same H1N1 virus (Fig. 7a). This improvement is remarkable in that the F10 IgG had already been refined to sub nano-M affinity through natural affinity maturation. These results indicate that the neutralizing potency of F10 can be further optimized specific to the strain of the antigen HA by modifying the amino acid sequences surrounding the key CDR residues; different H1N1 strain could require different combination of the peripheral amino acids to fine-tune the neutralizing potency and binding affinity.

The binding affinities of F10 IgG and H123#437 IgG to both BS/07 H1 HA and CA/09 H1 HA are about the same. Figure 7b shows the EC_50_ measurements for each of the antibody-antigen pairs, for which the EC_50_’s range from 2.5 to 2.9 ng/mL. The dissociation constant (K_D_) measured by BIAcore was 46 pM for F10 IgG binding to CA/09 H1 HA (k_on_ = 2.40 × 10^5^ (1/Ms); k_off_ = 1.11 × 10^−5^ (1/s)), which was slightly better than the K_D_ for H123#437 IgG binding to the same antigen (K_D_ = 91 pM; k_on_ = 3.86 × 10^5^ (1/Ms); k_off_ = 3.52 × 10^−5^ (1/s)). The sensorgrams and associated quality parameters for the K_D_ measurements are shown in Supplementary Figure S4. Although the K_D_ measurements were consistent with the EC_50_ measurements within the range of experimental error, these results did not match with the measurement that the normalized binding affinity of H123#437 scFv was about 5 folds better than that of F10 scFv. Minor structural differences of the VH domains in the scFv and IgG could lead to the k_off_ for the H123#437 IgG a few folds faster than anticipated.

Results in Fig. 7 indicate that a pair of neutralizing antibodies with similar affinity binding to two strains of HA could have substantially different neutralizing potency against the two corresponding strains of influenza virus. The two antibodies (F10 IgG and H123#437 IgG) are highly related in sequence and bind to the same epitope of HA with similar EC_50_ to CA/09 H1 HA and BS/07 H1 HA (Fig. 7b), but the neutralizing potency (IC_50_) for H123#437 IgG is 3 ~ 7 folds more potent than that of F10 IgG against the two strains of influenza virus (Fig. 7a), suggesting that although the general positive linear correlation between the binding affinity and neutralizing potency did exist to an extent (Fig. 6b), the affinity-function correlation was not applicable universally – affinity optimization alone does not necessarily lead to functionally optimized IgG antibodies (Fig. 7).

## Discussion

Synthetic antibody libraries can be designed and constructed to target a specific epitope on an antigen. Systematic exploration of the global sequence spaces of CDR-H1 ~ CDR-H3 showed that the CDR-H2 sequences of IGHV1–69-bnAbs are near the global optimum. These IGHV1–69 encoded CDR-H2 residues interact with the highly conserved membrane-proximal epitope patch in the interface of HA1 and HA2 of the HA stem region, supporting the speculation that the ubiquitous influenza virus infections in humans have exerted evolutionary pressure on the retention of IGHV1–69 germline gene in human genomes. The CDR-H1 sequence preferences were less well-defined, suggesting that the somatic mutations in this region could further optimize the neutralizing potency of the IGHV1–69-derived antibodies according to the specific strain of the HA antigen. The sequence and length of the CDR-H3 loop in the IGHV1–69-bnAbs are not critically conserved except for the two critical residues (Pro96 and Tyr98) in the proximity to the tip of the CDR-H2 loop. Although the key residue positions are highly conserved for the function of the IGHV1–69-bnAbs, the sequence preferences of the peripheral residue positions in the CDR loops can enhance the binding affinity and neutralizing potency by several folds specific to the strain of the HA antigen. These systematically elucidated principles were integrated in an antibody library designed with the aim to target the specific epitope of VH1–69-bnAbs on HA from diverse strains of H1N1 influenza virus. By contrast to the antibody library designs restricted to only one CDR region, the cooperative diversification of residue positions from all three CDR regions substantially improve binding affinity and neutralizing potency. The results suggest that well-designed synthetic antibody libraries could be used to discover functionally effective antibodies for many other strains of H1N1 influenza virus.

Neutralizing antibodies could be derived from a limited set of synthetic antibody libraries for the majority of the influenza virus strains. The sequence similarity between CA/09 H1 HA and BS/07 H1 HA are 72% in the HA1 region and 92% in the HA2 region. The membrane proximal and distal epitope regions of the two HAs are 100% identical in sequence. Hence it is not unexpected to observe the overall correlation of the normalized binding affinity between the two strains of H1 HA (Fig. 4a). Nevertheless, minor differences in sequence and structure of the HAs could lead to unpredictable affinity-neutralization relationships, as shown in Figs 4 and 7. Consequently, affinity maturation of neutralizing antibodies against one strain of HA does not necessarily lead to more potent neutralizing antibodies against another strain of virus, even when the two strains of virus are closely related as in CA/09 and BS/07 H1N1 viruses. That is, neutralizing antibodies need to be optimized against each individual virus – it could be extremely difficult, if not impossible, to find a universally optimized neutralizing antibody even for closely related viruses. The results in this work suggest that an antibody library for which the variants share common key CDR residues but with enough diversity of the peripheral residues could provide near-optimal neutralizing antibodies for each of the viruses in the subgroup. However, the antibody library is increasingly limited in terms of discovering neutralizing antibodies against viruses that are increasingly distant from the effective subgroup. Indeed, antibodies discovered from the F10-CDRH1~3 libraries did not cross-react to H3 HA (Fig. 5), suggesting that the synthetic antibody libraries herein were ineffective against viruses beyond the H1N1 subgroup. Nevertheless, the principles developed in this work could be applicable to develop synthetic antibody libraries to cover other subtypes of influenza virus. It can be envisioned that a limited number of synthetic antibody libraries could be eventually constructed capable of discovering near-optimal neutralizing antibodies against the majority of influenza viruses.

## Methods

### Cell line and viruses

Madin-Darby canine kidney (MDCK) epithelial cell line was cultured in Minimum Essential Medium Eagle (MEM) medium (Gibco/BRL) supplied with non-essential amino acids (NEAA), 2 mM L-glutamine, and 10% fetal bovine serum (FBS) in a 5% CO_2_ humidified atmosphere incubator at 37 °C. Influenza A viruses BS/07 H1N1 (A/Brisbane/59/2007,) and CA/09 H1N1 (a recombinant virus NYMC X-181, HA from A/California/07/2009), supplied from Taiwan’s CDC, were used in this study. Viruses’ stocks were propagated in 10-day-old embryonic eggs’ allantoic cavities, concentrated and resuspended in PBS. TCID_50_ (50% tissue culture infectious dose) was used to determine virus titer in MDCK cells according to Reed and Muench^20^.

### Phage-displayed synthetic antibody library construction

The primers shown in Supplementary Figure S2 were used to construct the antibody scFv libraries based on the well-established oligonucleotide directed mutagenesis protocol^12^,^16^,^21^,^22^. In brief, the parent phagemid templates were first constructed with the stop codon TAA encoded in designated positions as indicated in Supplementary Figure S2. The parent templates were then used to construct the antibody scFv libraries with the degenerated CDR sequences encoded with NNK, the locations of which are also delineated in Supplementary Figure S2. *E. coli* (strain ER2738) harboring the phagemid libraries was cultured to produce recombinant M13 phages expressing the antibody scFv libraries as pIII-fusion proteins on the phage particles, which were rescued with helper phage M13KO7^23^. The recombinant phages expressing the scFv libraries were precipitated by polyethylene glycol/NaCl (PEG/NaCl) and then resuspended in phosphate buffered saline (PBS) for the following selection-amplification cycles. The quality of the phage-displayed scFv libraries were assessed by tittering the colony forming units per mL (CFU/mL) and by random sequencing of the single *E. coli* colonies harboring the phagemids. The LOGOs for random sequences from all the synthetic libraries in this work are shown in Supplementary Figure S3. The tittering results suggested that all the synthetic phage-display libraries had complexity >10^9^ and the sequence LOGOs for the library variants (Supplementary Figure S3) indicated that the expression of the variable sequences were not substantially biased by phage library preparation.

### Phage display panning for anti-HA antibodies

Maxisorp 8-well strip (Nunc) coated with HA proteins (1 μg/100 μL PBS per well) was used for panning anti-HA antibodies based on the protocol described previously^24^. In brief, the wells were coated with HA by shaking the coating solution in the wells for 2 hrs at room temperature. The HA-coated wells were then treated with blocking buffer (5% skim milk in PBST (phosphate buffered saline with 0.1% tween-20)) for 1 hr at room temperature. Recombinant phages in the blocking buffer diluted to 10^12^ CFU/mL was added to the HA-coated wells for 1 hr with gentle shaking. The wells were then washed vigorously 10 times with PBST, followed by 6 times with PBS to remove nonspecific binding phages. The bound phages were eluted (0.1 M HCl/glycine (pH 2.2) for 10 min), and the elution solution was neutralized immediately by 2 M Tris-base buffer (pH9.0). *E. coli* strain ER2738 (OD_600_ = ~0.6) was used for phage infection at 37 °C for 30 min; non-infected *E. coli* was eliminated by treating with ampicillin for 30 min. After ampicillin treatment, helper phage M13KO7 was added for another 1 hr incubation. Selected phages in the *E. coli* culture were amplified with vigorously shaking overnight at 37 °C in the presence of kanamycin. The amplified phages were precipitated in PEG/NaCl, and then resuspended in PBS for the next selection-amplification cycles.

### Binding and concentration characterization of phage-free soluble scFv in culture supernatant

*E. coli* strain ER2738 grown in the mid-log phase in 2YT broth (16 g/L tryptone, 10 g/L yeast extract, 5 g/L NaCl, pH 7.0) in deep well plates was infected with single-clonal phages harboring a selected scFv gene in their phagemids. After one hour incubation at 37 °C with shaking, ampicillin was added to the final concentration of 100 μg/mL. IPTG was added to final concentration of 1 μg/mL until broth OD_600_ reach 1.0–1.2. The plates were incubated at 37 °C overnight with rigorously shaking. After the spin-down of the bacteria in the cell culture solution, the supernatant with phage-free soluble scFv (expressed with E-tag fused to the C-terminus) was filtrated by AcroPrep 96 Filter Plate with 0.45 μm GHP membrane (PN5054, Pall corporation) to remove bacteria from contaminating the following micro-neutralization assays.

For soluble scFv binding test, ELISA assay was carried out following the previous methods^25^ with some modifications. In brief, 96-well Maxisorp microtiter plate (Nunc) was coated with BS/07 or CA/09 H1 HA (0.5 μg/100 μL PBS per well) for 2 hrs with shaking at room temperature. After treated with 300 μL of blocking buffer for 1 hr, 50 μL of secreted scFv in the filtered supernatant was mixed with fresh 50 μL of blocking buffer and then added to the coated microtiter plate for another 1 hr under gently shaking. Goat anti-E-tag antibody (conjugated with HRP, 1:4000, Cat. No. AB19400, Abcam) was added to the microtiter plate for 1 hr. SureBlue TMB microwell peroxidase substrate (100 μL per well) was added to the wells and the absorbance at 450 nm was measured after reactions were stopped by adding 1N HCl (100 μL per well). Each scFv in its respective filtered supernatant was assayed in triplicate.

The concentration of the scFv in its respective filtered supernatant was measured with the dot-blot procedure: PVDF membrane rinsed with methanol and equilibrated with PBS buffer was immobilized on Bio-Dot Apparatus Assembly (Bio-Rad). Samples of secreted scFv in culture broth were added into each well. Serially diluted F10-E-tag scFv of known concentration was also loaded to establish standard controls. The membranes were blocked with 5% skim milk in PBST, and then developed with anti E-tag antibody (conjugated with HRP, Abcam) followed by chromogenic TMB peroxidase substrate treatment.

Normalized binding affinities of scFvs were the ELISA readings divided by concentration quantification, and normalized with that of the F10 control, i.e., (E_S_/Q_S_)/(E_F10_/Q_F10_), where E_S_ and E_F10_ were ELISA readings of the sample and the F10 controls respectively, and Q_S_ and Q_F10_ were concentration quantification of the sample and the F10 control respectively. The averaged values and the standard deviations of the normalized binding affinities were calculated with multiple measurements^26^. Pearson’s correlation coefficient (r) and the p-value (p) of Student’s t-test were calculated with Microsoft Excel.

### Micro-neutralization assay for influenza virus infection

MDCK cells (3 × 10^4^ cell per well) were seeded in 96-well plates and cultured for 16 hrs to confluency. Filtrated scFvs were mixed with 100 TCID_50_ freshly diluted viral solution for 30 min at 37 °C. Virus-scFv mixtures were then added to infect PBS-washed MDCK cells for 1 hr at 37 °C. After absorption, virus-scFv mixtures were removed and MDCK cells were washed with PBS twice. Infected MDCK cells were cultured and fixed with methanol-acetone (1:1 (v/v)) 24 hrs post-infection. After fixation, MDCK cells were treated with 0.5% Triton X-100 in PBS for 5 min, and then with blocking buffer for 1 hr. The mouse anti-influenza A viral nucleoprotein IgG antibody (1:2000, Cat. No. MAB8251, Millipore) was used for detection of viral nucleoprotein production with goat anti-mouse antibody-HRP (Cat. No. 12–349, Millipore) and TMB peroxidase substrate. Each filtrated scFv was assayed in quadruplicates.

The ELISA readings of virus-only control was set as 0%, and that of negative control (no virus and no scFv) was set as 100%. The reduction of virus infection due to scFv addition was calculated as neutralization percentage. Normalized neutralizing potency was the neutralization percentage divided by concentration quantification, and normalized with that of F10., i.e., (N_S_/Q_S_)/(N_F10_/Q_F10_), where N_S_ and N_F10_ were the neutralization percentage of the sample scFv and the F10 scFv control respectively, and Q_S_ and Q_F10_ were the concentration quantification of the sample scFv and the F10 scFv control respectively. The averaged values and the standard deviations of the normalized neutralizing potencies were calculated with multiple measurements^26^. Pearson’s correlation coefficient (r) and the p-value (p) of Student’s t-test were calculated with Microsoft Excel.

### Sequence LOGO

The size *R*_*ji*_ (in half-bit unit) for amino acid type *i* at position *j* in the consensus sequence LOGOs were calculated with the equation:


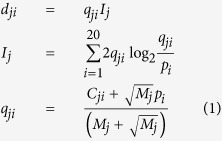


*C*_*ji*_ is the count for amino acid *i* at position *j* in *M*_*j*_ count of HA-binding CDR sequences containing position *j*; *p*_*i*_ is the background probability for amino acid *i* encoded in the NNK degenerate codon^27^; the square root of *M*_*j*_ in the equation is the pseudo count to prevent singularity when *C*_*ji*_ equals to zero. Equation (1) is modified after the original formulation^28^.

### Position specific score matrix (PSSM) and scoring of germline sequences

The position specific score matrix (PSSM) *W*_*ji*_, are expressed in half-bite units calculated with the Bayesian prediction pseudo-count method^27^,^29^:


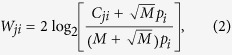


where *W*_*ji*_ is the preference for amino acid *i* at position *j* in the CDR of the antibody; *C*_*ji*_ is the count for amino acid *i* at position *j* in *M* count of HA-binding CDR sequences containing position *j*; *p*_*i*_ is the background probability for amino acid *i* encoded in the NNK degenerate codon^27^; the square root of *M* in the equation is the pseudo count to prevent singularity when *C*_*ij*_ equals to zero.

The matching score of a germline CDR sequence to a set of *M* antibody CDR sequences was calculated with the PSSM described in the previous paragraph. The germline sequences were retrieved from IMGT (http://www.imgt.org/). CDR H1 and CDR H2 of each germline sequence were first identified with the previous published method^30^; the matching score is the sum of *W*_*ji*_ (Equation (2)) over the entire CDR sequence, where *i* is the germline amino acid type at the corresponding position *j* in the CDR sequence.

### HA sequence analysis

All full length HA sequences were downloaded from Influenza Research database (as of Dec 2014)^31^. A total of 28627 sequences of Influenza A from all hosts and subtypes were collected for identifying amino acid conservations of membrane-proximal epitopes and membrane-distal epitopes. The HA sequences were aligned within each subtype using HMMER package^32^ with Hidden Markov model from Pfam protein families database^33^. The statistic details are summarized in Supplementary Table S1.

### Other experimental details

Other experimental details, including expression and purification of hemagglutinin in insect cells, construction and expression of scFv and IgG, antibody-antigen interaction affinity and kinetics measurements by surface plasmon resonance, and IC_50_ measurements are described in details in Supplementary Methods.

## Additional Information

**How to cite this article**: Tung, C.-P. *et al.* Discovering neutralizing antibodies targeting the stem epitope of H1N1 influenza hemagglutinin with synthetic phage-displayed antibody libraries. *Sci. Rep.* **5**, 15053; doi: 10.1038/srep15053 (2015).

## Supplementary Material

Supplementary Information
