# Supplementary material for: Discovering neutralizing antibodies targeting the stem epitope of H1N1 influenza hemagglutinin with synthetic phage-displayed antibody libraries
Source: Sci Rep. 2015 Oct 12;5:15053. doi: 10.1038/srep15053 (PMC4601012; doi:10.1038/srep15053)
Supplement: Supplementary Information [file srep15053-s1.pdf]

Supplementary Information

Discovering neutralizing antibodies targeting the stem epitope of H1N1 influenza hemagglutinin with synthetic phage-displayed antibody libraries

Chao-Ping Tung[a,d], Ing-Chien Chen[a,d], Chung-Ming Yu[a,d], Hung-Pin Peng[a], Jhih-Wei Jian[a,b,c], Shiou-Hwa Ma[a], Yu-Ching Lee[a], Jia-Tsrong Jan[a], An-Suei Yang[a,*]

[a] Genomics Research Center, Academia Sinica, Taipei, Taiwan 115.

[b] Institute of Biomedical Informatics, National Yang-Ming University, Taipei, Taiwan 112.

[c] Bioinformatics Program, Taiwan International Graduate Program, Institute of Information Science, Academia Sinica, Taipei, Taiwan 115.

[d] These authors contribute equally.

[*] Correspondence should be addressed to: An-Suei Yang, Genomics Research Center, Academia Sinica, 128 Academia Rd., Sec.2, Nankang Dist., Taipei, Taiwan 115. Phone: +886-2-2787-1232 email: yangas@gate.sinica.edu.tw

Running title: HA-specific synthetic antibody library

Keywords: influenza virus, phage display, synthetic antibody library, antibody design, hemagglutinin

Contents

Supplementary Methods

Expression and purification of hemagglutinin (HA) in insect cells

Expression and purification of scFv

Construction and expression of IgG

Antibody-antigen interaction affinity and kinetics measurements by surface plasmon resonance

IC50 measurement

Supplementary Figures

Supplementary Figure S1. Structures of membrane-distal and membrane-proximal epitopes and paratopes in the complex structures of HA and stem-specific IGHV1-69-bnAb.

Supplementary Figure S2. Designs and constructions of synthetic antibody libraries.

Supplementary Figure S3. Sequence LOGO of random variants in the phage-displayed scFv libraries: F10-CDRH1, F10-CDRH2, F10-CDRH3, and F10-CDRH123.

Supplementary Figure S4. Sensorgrams and quality parameters for H123#437 IgG and F10 IgG binding to CA/09 H1 HA.

Supplementary Tables

Supplementary Table S1. Amino acid conservations of membrane-proximal epitopes and membrane-distal epitopes from HA sequences of all subtypes of Influenza A viruses in the Influenza Research database.

Supplementary Table S2. Data associated with Figure 1 for the scFv variants selected from the F10-CDRH2 library.

Supplementary Table S3. Data associated with Figure 2 for the scFv variants selected from the F10-CDRH1 library.

Supplementary Table S4. Data associated with Figure 3 for the scFv variants selected from the F10-CDRH3 library.

Supplementary Table S5. Data associated with Figure 6b for the scFv variants selected from the F10-CDRH123 library.

Supplementary Table S6. Data associated with Figure 6c for the purified scFv variants selected from the F10-CDRH123 library.

Supplementary References

Supplementary Methods

Expression and purification of hemagglutinin (HA) in insect cells

cDNAs corresponding to residues 18-528 of the ectodomain of the hemagglutinin (HA) from A/Brisbane/59/2007 (H1N1; Accession No. ACA28844.1, BS/07 H1 HA) and residues 18-529 from the A/California/07/2009 (H1N1; Accession No. ACP41953.1, CA/09 H1 HA) were codon-optimized for eukaryotic cell expression and fused to an N-terminal gp67 signal peptide (MLLVNQSHQGFNKEHTSKMVSAIVLYVLLAAAAHSAFAADLAS) and to a C-terminal thrombin cutting site, trimerization domain and His_6-tag (ASLVPRGSPGSGYIPEAPRDGQAYVRKDGEWVLLSTFLGHHHHHH) by PCR as described previously [1]. These HA expression cassettes were inserted into pFastBac-1 (Invitrogen), a baculoviral transfer vector. HA protein was produced by infecting suspension cultures of Sf9 cells (3 x 10^6 cell/mL) (Invitrogen) with recombinant baculovirus at an MOI of 5 and incubated at 26°C shaking at 110 rpm for 72 hrs. The cultures were clarified by two rounds of centrifugation (1,000 x g and 12,000 x g for 30 min, 4°C). The supernatants containing HA was dialyzed by PBS, pH 7.2 for overnight at 4°C. Before Ni^{2+}-charged IMAC column binding, the dialyzed solution was filtered by 0.8 μm pore size filter. The HA was purified by Ni^{2+}-charged IMAC column chromatography (GE Healthcare Life Sciences) by gradient from 40 mM to 500 mM imidazole in TS solution (Tris-HCl, 10 mM, NaCl 50 mM, pH 8.0). The fractions containing HA were introduced to Q column chromatography (GE Healthcare Life Sciences) and eluted by gradient from 40 mM to 1000 mM NaCl in Tris-HCl, 10 mM, pH 8.0. The fractions containing HA were concentrated and introduced to Superdex200 10/300 GL column (GE Healthcare Life Sciences) for size

exclusion chromatography with TS solution. The fractions containing HA were collected and stored in 4°C or -80°C with proteinase inhibitor cocktail.

Expression and purification of scFv

The expression and purification of the scFv followed the method described previously with minor modifications [2]. In brief, the scFv coding region was subcloned into pET-32 expression vector encoding thioredoxin as a fusion protein N-terminal to the scFv. The fusion protein contains a hexa-His tag followed by a TEV protease cutting site between the thioredoxin and the scFv, which is followed by an Avitag oligopeptide (GLNDIFEAQKIEWHE, Avidity Inc., USA) appending to the C-terminus of the scFv for *in vivo* biotinylation. The scFv gene derived from phage panning was subcloned into the expression vector via the *Sfi*I and *Not*I cutting sites encompassing the scFv coding region. *E. coli* Rosetta-gami B(DE3) (Novagen) strain culture transformed with scFv expression vector was grown in 2X YT medium (Tryptone 16 g/L, Yeast extract 10 g/L, NaCl 5 g/L) with ampicillin (200 μg/L), tetracycline (12.5 μg/L) and chloramphenicol (37.5 μg/L) at 37°C until OD_{600} reached 1.0, and was then incubated at 16°C for another 2hr before adding 0.2 mM IPTG. After overnight protein expression and centrifugation, the cell pellets were resuspended in lysis buffer (Tris-HCl, 50 mM, pH 8.0, 150 mM NaCl, 30 mM imidazole) and the suspended cells were then broken by Microfluidizer (Microfluidics, MA). The recombinant thioredoxin-scFv fusion protein was purified by nickel chelation chromatography with IMAC prepacked column (GE Healthcare Life Sciences) charged by 0.1 M $NiSO_4$ solution. The fractions containing the fusion protein were collected and dialyzed by Tris-HCl, 50 mM, pH 7.5 (the theoretical pI of sc-dsFv was 5.82) overnight at 4°C or desalted by HiPrep 26/10 desalting column (GE

Healthcare Life Sciences) with the same buffer. The protein solution was then introduced to ion-exchanged chromatography (prepacked Q column, GE Life Healthcare Sciences). The fractions containing the thioredoxin-scFv fusion protein were collected and treated with His_6-tagged TEV protease (A_{280} ratio 50:1) at 30°C for at least 5 hr but not longer than 8 hr. The TEV-cleaved fragment containing His_6-tagged thioredoxin and the His_6-tagged TEV protease were removed by nickel chelation chromatography. The fractions containing scFv were further purified with a Superdex75 size-exclusion column (GE Healthcare Life Sciences) in SEC buffer (Tris-HCl, 50 mM, pH 7.5, 400 mM NaCl, 10% glycerol). The soluble scFv protein was prepared with 95% purity. The purified sc-dsFv was stored at 4°C for a least one week without affinity loss.

Construction and expression of IgG

VH and VL DNA fragments were PCR amplified separately. The VH and VL DNA fragments were assembled into the plasmid for IgG1 expression with the pIgG expression system (U.S. patent no. 5736137) with Gibson assembly cloning kit (NEB). IgGs were expressed by 293F cells. 293F cells were subcultured to a final 1-1.5 x 10^6 cells/mL in Gibco® FreeStyle™ 293 Expression Medium (Invitrogen, cat. 12338), and incubated for 2-4 hrs at 37°C (8% CO_2, 110 rpm). Each IgG construction was transfected into 293F cells with PEI (Polysciences Inc., cat. 24765) according to supplier's protocol. Expressed IgG was collected from cell culture supernatant 7-9 days or until cell viability drops below 60% after transfection. Protein A Sepharose (GE Healthcare, cat 17-0780-01) was reconstituted and packed as manufacture's instruction described. The 293F cell cultures were centrifuged and filtrated through

0.22 μm filter. The IgG was purified through protein A column, eluted with 0.2 M glycine/HCl buffer, pH 2.5, and neutralized with 1 M Tris buffer, pH 9.0.

Antibody-antigen interaction affinity and kinetics measurements by surface plasmon resonance

BIAcore T200 (GE Healthcare) instrument was used to determine the binding affinities and kinetic parameters for interactions between IgGs and HA protein. HA in 10 mM acetate buffer (pH 5.0) was immobilized on a CM5 sensor chip to a response unit (RU) of 1000 with an amine coupling kit (GE Healthcare). Association (k_{on}) and dissociation (k_{off}) constants of the interactions between IgGs and HA were measured in HBS-EP+ buffer with a flow rate of 30 μL/min. The sensor surface was regenerated with 50 mM HCl with 0.05% Surfactant P20, prior to a new IgG injection and the signals obtained were subtracted by that obtained from the reference channel that had not been coated with ligands. Binding kinetics was determined by global fitting to 1:1 binding model using the Biaevaluation software (GE Healthcare).

IC_{50} measurement

The half of maximal inhibitory concentration (IC_{50}) was determined to evaluate neutralization ability of IgG. MDCK cells were seeded in 96-well plates and cultured for 16 hrs to confluency. Virus stock was freshly diluted by infection buffer. Purified IgG diluted with PBS were mixed with 100 $TCID_{50}$ viral solution for neutralization. Virus-IgG mixtures were then added to infect PBS-washed MDCK cells. After absorption, virus-scFv mixtures were removed and MDCK cells were washed with PBS and cultured in fresh infection buffer, and fixed with methanol-acetone (1:1 (v/v)) 24 hrs postinfection. After fixation, MDCK cells were treated with 0.5% Triton

X-100 in PBS and then blocking buffer. Mouse anti-influenza A viral nucleoprotein IgG antibody (Cat. No. MAB8251, Millipore) and HRP conjugated goat anti-mouse antibody (Cat. No. 12-349, Millipore) with TMB peroxidase substrate were used to monitor the virus propagation during antibody treatment. Each concentration of diluted IgG was assayed six replicates. The IC_{50} (ng/mL) was calculated according to Stewart and Watson method [3].

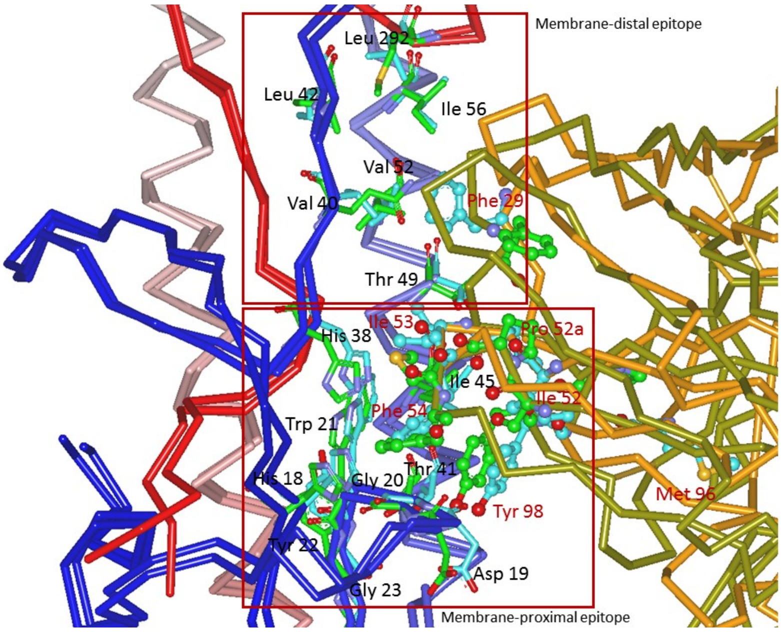

Supplementary Figure S1. Structures of membrane-distal and membrane-proximal epitopes and paratopes in the complex structures of HA and stem-specific IGHV1-69-bnAb. The complex structures are PDB code 3FKU [4] for H5N1 HA (backbone shown in blue-to-red for N-to-C coloring) and F10 antibody (backbone colored in green) and PDB code 3GBN [1] for H1N1 HA (backbone shown in blue-to-red for N-to-C coloring) and CR6261 antibody (backbone colored in orange). The HAs from both complex structures are superimposed with minimal RMSD to show the relative interfaces of the HA-antibody interaction. Residues from HA are shown in stick model; residues from the VH domain of the antibodies are shown in ball-and-stick model (residues from 3FKU are colored in green; residues from 3GBN are colored in cyan). The amino acids are labeled in amino acid type and residue number based on the structures in 3GBN. The residue labels for HA amino acids are colored in black; the residue labels for antibody amino acids are colored in red. The membrane-distal epitope and membrane-proximal epitope residue comparisons for diverse strains of HA are shown in Supplementary Table 1.

```
Kabat No.          1                         10                            20                            30                            40
                   |                          |                             |                             |                             |
                  CAGGTTCAGCTGGTTCAGAGCGGTGCAGAAGTTAAAAAACCGGGTAGCAGCGTTAAAGTTAGCTGTACCAGCAGCGAAGTTACCTTTAGCAGCTTTGCAATTAGCTGGGTTCGTCAGGCA
                   Q  V  Q  L  V  Q  S  G  A  E  V  K  K  P  G  S  S  V  K  V  S  C  T  S  S  E  V  T  F  S  S  F  A  I  S  W  V  R  Q  A
                  <------------------------------------FR1----------------------------------><-------CDRH1-------><----------FR2----------
Template_1                                                                     GCTGTACCAGCAGCGAAGTTTAATAAGAATTCTTTGCAATTAGCTGGGTTCGTCA
F10-CDRH1_1                                                     CAGCGTTAAAGTTAGCTGTACCNNKNNKNNKNNKNNKNNKNNKNNKNNKGCAATTAGCTGGGTTCGTCAG
F10-CDRH123_1                                                       GTTAAAGTTAGCTGTACCAGCAGCGAANNKNNKTTTCCGNNKTTTGCAATTAGCTGGGTTCGTC

Kabat No.         41                         50    52  A                      60                            70
                   |                          |     |  |                       |                             |
                  CCGGGTCAGGGTCTGGAATGGCTGGGTGGTATTAGCCCGATGTTTGGCACCCCGAATTATGCACAGAAATTTCAGGGTCGTGTTACCATTACCGCAGATCAGAGCACCCGTACCGCATAT
                   P  G  Q  G  L  E  W  L  G  G  I  S  P  M  F  G  T  P  N  Y  A  Q  K  F  Q  G  R  V  T  I  T  A  D  Q  S  T  R  T  A  Y
                  --------------FR2---------------><------CDRH2-----><--------------------------------FR3---------------------------------
Template_2                             CTGGGTGGTATTAGCCCGTAATAAGAATTCCCGAATTATGCACAGAAATTTCAG
F10-CDRH2_1                   CTGGAATGGCTGGGTGGTATTNNKNNKNNKNNKNNKNNKCCGAATTATGCACAGAAATTTCAG
F10-CDRH123_2                        GCTGGGTGGTATTAGCCCGATGTTTNNKNNKCCGAATTATGCACAGAAATTTCAG

Kabat No.         80    82  A  B  C                      90                           100  A  B  C  D  E  F  G
                   |     |  |  |  |                       |                             |  |  |  |  |  |  |  |
                  ATGGATCTGCGTAGCCTGCGTAGTGAAGATACCGCAGTGTATTATTGTGCACGTAGCCCGAGCTATATTTGTAGCGGTGGCACCTGTGTTTTTGATCATTGGGGTCAGGGCACCCTGGTG
                   M  D  L  R  S  L  R  S  E  D  T  A  V  Y  Y  C  A  R  S  P  S  Y  I  C  S  G  G  T  C  V  F  D  H  W  G  Q  G  T  L  V
                  ----------------------FR3----------------------------><-------------------CDRH3----------------------><-------FR4----------
Template_3                                                        ACGTAGCCCGAGCTATATTTGTTAATAAGAATTCTGTGTTTTTGATCATTGGGGTCA
F10CDRH3_1                                         CAGTGTATTATTGTGCACGTAGCNNKNNKNNKNNKNNKNNKNNKNNKNNKNNKNNKGTTTTTGATCATTGGGGTCAGG
F10CDRH123_3                                          GTATTATTGTGCACGTAGCCCGNNKTATNNKNNKNNKNNK---------GTTTTTGATCATTGGGGTCAGG
```

Supplementary Figure S2. Designs and constructions of synthetic antibody libraries. The VH domain DNA and amino acid sequences of the parent scFv are shown with Kabat numbering for amino acid residue positions. The primers for constructing each of the antibody libraries are shown below the VH amino acid sequence: for the F10-CDRH1 library, the Template_1 primer was used to construct the template with stop codon (TAA) in the CDR-H1 region, and the library was diversified with the primer F10-CDRH1_1 over the template; for the F10-CDRH2 library, the Template_2 primer was used to construct the template, and the library was diversified with the primer F10-CDRH2_1 over the template; for the F10-CDRH3 library, the Template_3 primer was used to construct the template, and the library was diversified with the primer F10-CDRH3_1 over the template; for the F10-CDRH123 library, the Template_1~3 primers were used simultaneously to construct the template, and the library was diversified with the primer F10-CDRH123_1~3 simultaneously over the template.

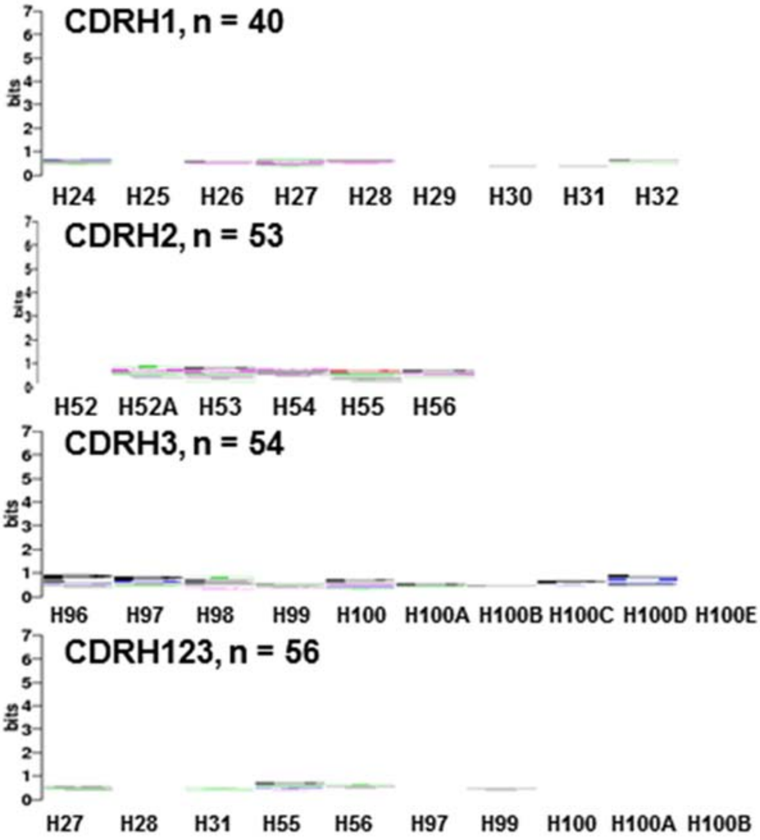

Supplementary Figure S3. Sequence LOGO of random variants in the phage-displayed scFv libraries: F10-CDRH1, F10-CDRH2, F10-CDRH3, and F10-CDRH123. The LOGO was calculated with the background probabilities based on the degenerate codon NNK. The formula for LOGO calculation is shown in Methods.

a

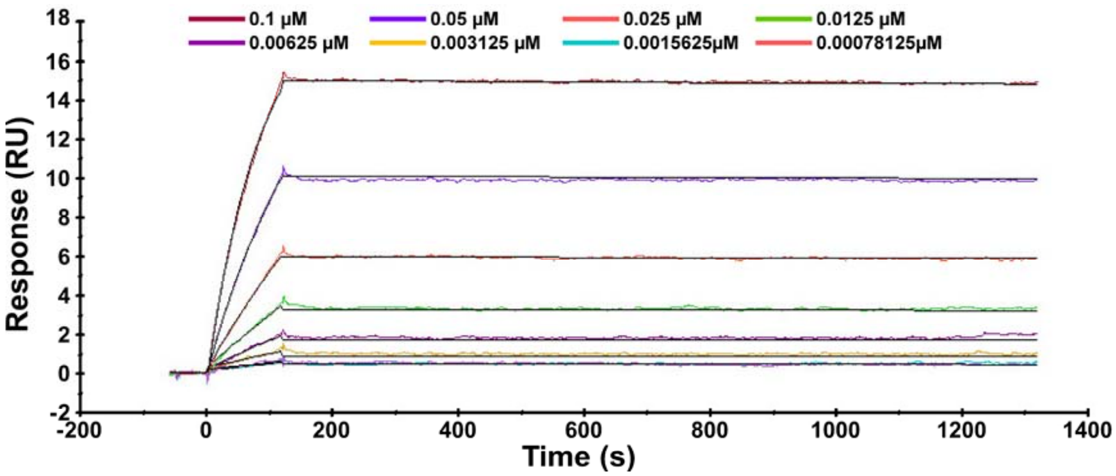

b

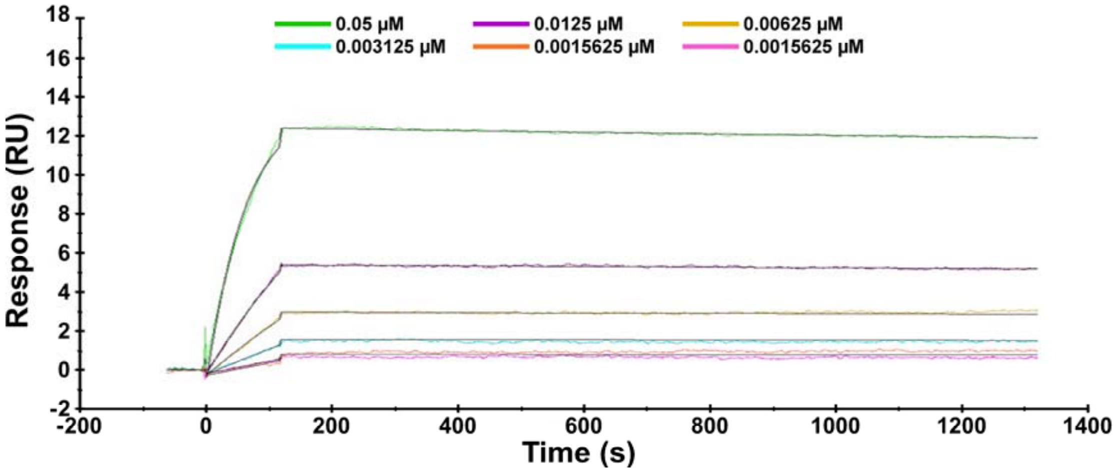

Supplementary Figure S4. Sensorgrams and quality parameters for H123#437 IgG and F10 IgG binding to CA/09 H1 HA. Sensorgrams of F10 (a) and H123#437 IgG (b). The affinity of F10 and H123#437 IgG were measured against CA/09 H1 HA as 46 (k_{on} = 2.40×10^5 (1/Ms); k_{off} = 1.11×10^{-5} (1/s); RU^2=0.0100) and 91 pM (k_{on} = 3.86×10^5 (1/Ms); k_{off} = 3.52×10^{-5} (1/s); RU^2=0.0116) respectively. For H123#437, the kinetic constants were within instrument specifications, and appeared to be uniquely determined. No significant bulk contributions (RI) were found. The kinetic constants were approaching the detection limits in F10 IgG. CA/09 H1 HA in 10 mM acetate

buffer (pH 5.0) was immobilized on a CM5 sensor chip to a response unit (RU) of 1000. Association (k_{on}) and dissociation (k_{off}) constants of the interactions between IgGs and CA/09 H1 HA were measured in HBS-EP+ buffer with a flow rate of 30 μL/min. The sensor surface was regenerated with 50 mM HCl with 0.05% Surfactant P20, prior to a new IgG injection and the signals obtained were subtracted by that obtained from the reference channel that had not been coated with ligands. Binding kinetics was determined by global fitting to 1:1 binding model using the Biaevaluation software (GE Healthcare).

Supplementary Table S1. Amino acid conservations of membrane-proximal epitopes and membrane-distal epitopes from HA sequences of all subtypes of Influenza A viruses in the Influenza Research database. Only amino acid types showing frequencies higher than 1% are shown. The most predominant amino acid types are shown first, followed by other minor amino acid types after slash. The bold character represents the amino acid type with frequency higher than 98%. The third column from left indicates the number of sequences in the Influenza Research database (as of Dec 2014) belonging to the specific subtype. The residue position numbering scheme (third row from top) follows the crystal structure of CR6261-HA complex (PDB ID: 3GBN).

.

			Membrane-distal epitopes						Membrane-proximal epitopes								
			HA1			HA2			HA1		HA2						
	Subtype	Number	40	42	292	49	52	56	18	38	19	20	21	22	23	41	45
Group1	H1	5935	**V**/I	**L**	**L**	T/S	**V**	**I**	**H**	**H**	**D**	**G**	**W**	**Y**	**G**	**T**	**I**
	H2	510	K/Q	**I**	**L**	**T**	**V**	**I**	**H**	**H**	**D**	**G**	**W**	**Y**	**G**	**T**	I/FV
	H5	3614	**Q**	**I**	M/L	**T**	**V**	**I**	**H**	**H**	**D**	**G**	**W**	**Y**	**G**	**T**	**I**/V
	H6	1298	V/I	**L**	**L**	**T**	**V**	**I**	**H**	**H**	**D**	**G**	**W**	**Y**	**G**	**T**	I/V
	H8	115	**M**	**L**	K/R	**T**/S	**V**/I	V	**Q**	**Q**	**D**	**G**	**W**	**Y**	**G**	**T**	**I**
	H9	1525	**K**	**L**	**L**	**T**	**V**	V/I	**Q**	**H**	**A**	**G**	**W**	**Y**	**G**	**T**	I/V
	H11	492	V/I	**L**	R/K	**T**	**V**	**V**	**L**	**S**	**N**	**G**	**W**	**Y**	**G**	**T**	I/V
	H12	148	**E**	**L**	**K**	**Q**	**L**	**I**	**Q**	**Q**	**A**	**G**	**W**	**Y**	**G**	**T**	I/M
	H13	82	V/I	**L**	K/R	**T**	**I**	**I**	**L**	**S**	**N**	**G**	**W**	**Y**	**G**	**T**	**I**
	H16	40	V/I	**L**	K/R	**T**	**I**	**I**	**L**	**S**	**N**	**G**	**W**	**Y**	**G**	**T**	I/L
	H17	3	**Q**	**I**	**L**	**T**	**V**	**I**	**Q**	**G**	**D**	**G**	**W**	**Y**	**G**	**T**	**V**
Group2	H3	11651	**T**	**L**	**K**/R	N/T	**L**	**I**	**H**	**N**	**D**	**G**	**W**	**Y**	**G**	**T**	**I**
	H4	1217	**Q**	**L**	**K**	**N**	**L**	**I**/V	**H**	T/A	**D**	**G**	**W**	**Y**	**G**	**T**	**I**
	H7	1404	**T**	**T**	**L**	**T**	**L**	**I**	**H**	**N**	D/N	**G**	**W**	**Y**	**G**	**T**	**I**/V
	H10	570	**T**	**T**	**L**	**T**	**L**	**I**/V	**H**	**N**	**D**	**G**	**W**	**Y**	**G**	**T**	**I**/V
	H14	10	**K**	**L**	**K**	**N**	**L**	I/V	**H**	**S**	**D**	**G**	**W**	**Y**	**G**	**T**	**I**
	H15	13	**T**	**T**	**L**	**T**	**L**	**I**	**H**	**N**	**D**	**G**	**W**	**Y**	**G**	**T**	**I**

Supplementary Table S2. Data associated with Figure 1 for the scFv variants selected from the F10-CDRH2 library. The 1st column from left shows the name assigned to the scFv variants; the 2nd column shows the sequence, where 'q' represents expressed 'Gln' encoded by the TAG amber codon; the 3rd column shows the concentration of phage-free soluble scFv in culture supernatant; 4th~5th columns show the normalized neutralizing potency against BS/07 H1N1 and CA/09 H1N1 virus respectively; the 6th column shows the normalized binding affinity to BS/07 H1 HA. Three independent measurements were carried out to derive each averaged measurement and standard deviation for each of the scFv variants.

scFv	sequence	concentration (μg/ml)			normalized neutralizing potency against BS/07 H1N1			normalized neutralizing potency against CA/09 H1N1			normalized binding affinity to BS/07 H1N1		
F10	SPMFGT	1.347	±	0.109	1.000			1.000			1.000		
H2-75	SPLFqT	0.137	±	0.051	2.734	±	1.175	-15.935	±	20.688	0.506	±	0.195
H2-76	SPLFNT	0.434	±	0.071	2.166	±	0.722	-4.995	±	5.808	0.958	±	0.166
H2-73	APLFGQ	0.166	±	0.036	1.994	±	1.650	-6.553	±	13.715	0.827	±	0.185
H2-143	SPMFHq	0.061	±	0.001	1.819	±	2.246	1.626	±	3.953	0.718	±	0.068
H2-165	SPAFNH	0.163	±	0.011	1.812	±	2.106	1.446	±	0.972	1.057	±	0.116
H3-143	SPMLGq	0.318	±	0.004	1.744	±	0.652	1.211	±	0.464	1.470	±	0.507
H2-26	SPMFQM	0.171	±	0.004	1.705	±	1.064	0.002	±	0.458	1.646	±	0.229
H2-55	SPMFqI	0.186	±	0.012	1.549	±	0.659	1.539	±	0.351	1.463	±	0.210
H2-181	SPLFQI	0.129	±	0.003	1.426	±	1.218	2.402	±	1.318	0.820	±	0.085
H2-167	APAFNL	0.119	±	0.010	1.399	±	1.340	1.479	±	2.434	1.704	±	0.206
H2-63	SMMFNq	0.094	±	0.005	1.304	±	0.718	0.996	±	0.302	0.851	±	0.119
H3-142	SPqFPR	0.451	±	0.036	1.287	±	0.471	0.411	±	0.492	1.145	±	0.404
H2-64	SMMFNq	0.115	±	0.011	1.268	±	0.356	0.218	±	1.157	1.485	±	0.241
H2-81	SPMFNT	0.194	±	0.021	1.256	±	0.817	1.169	±	1.499	2.689	±	0.982
H2-23	SPqFPR	0.083	±	0.003	1.244	±	0.947	1.866	±	0.732	2.052	±	0.275
H2-43	SPMFNT	0.133	±	0.006	1.239	±	0.571	1.929	±	0.561	2.397	±	0.339
H2-39	SPLFHq	0.132	±	0.005	1.198	±	0.498	0.752	±	0.356	0.588	±	0.191
H2-168	SPLFSq	0.082	±	0.005	1.182	±	2.566	0.745	±	1.983	0.485	±	0.049
H2-51	SPLFNT	0.243	±	0.010	1.151	±	0.371	0.820	±	0.362	1.345	±	0.187
H2-48	TPMFEH	0.069	±	0.005	1.129	±	0.519	-0.032	±	0.873	0.824	±	0.121
H2-174	TPMFQR	0.096	±	0.002	0.956	±	3.322	0.681	±	1.120	0.440	±	0.043
H2-178	SPLFNI	0.167	±	0.008	0.939	±	1.259	1.612	±	1.144	1.657	±	0.157
H2-31	SPLFNI	0.179	±	0.004	0.893	±	0.459	0.907	±	0.211	1.302	±	0.183
H2-22	SPMLQI	0.145	±	0.006	0.881	±	0.290	1.162	±	0.782	1.265	±	0.407
H2-24	SPMFNY	0.132	±	0.001	0.827	±	0.346	1.788	±	0.525	1.626	±	0.220
H2-53	SPMLGq	0.102	±	0.011	0.808	±	0.928	1.461	±	0.460	0.693	±	0.126
H2-173	SPLFNS	0.198	±	0.006	0.777	±	1.812	0.862	±	0.613	1.234	±	0.111

H2-50	SPMFEN	0.072	±	0.001	0.769	±	0.560	-1.182	±	1.016	1.911	±	0.252
H2-166	SPLFNN	0.102	±	0.011	0.752	±	1.574	0.527	±	1.164	1.407	±	0.201
H2-34	SPLFHq	0.145	±	0.001	0.730	±	1.105	-0.176	±	0.487	1.129	±	0.177
H2-38	SPLFHq	0.153	±	0.001	0.680	±	0.364	1.125	±	0.530	1.182	±	0.156
H2-54	SPqFQI	0.082	±	0.001	0.545	±	0.806	1.684	±	0.718	0.858	±	0.122
H2-41	SPAFGF	0.137	±	0.006	0.539	±	0.197	0.416	±	0.417	0.954	±	0.150
H2-21	SPLLqY	0.108	±	0.002	0.500	±	0.220	1.136	±	0.744	0.430	±	0.114
H2-183	SPLFqN	0.121	±	0.007	0.457	±	1.594	-0.190	±	0.381	0.840	±	0.089
H2-146	SPMLNY	0.259	±	0.025	0.454	±	0.693	-0.148	±	1.050	1.093	±	0.143
H2-175	STMFNI	0.116	±	0.010	0.446	±	0.809	1.356	±	1.555	0.679	±	0.081
H2-32	SPQFQI	0.127	±	0.003	0.424	±	0.568	0.560	±	0.663	0.828	±	0.111
H2-47	SPMMNL	0.094	±	0.003	0.375	±	0.800	0.977	±	0.682	1.766	±	0.240
H2-169	SPAFSA	0.102	±	0.003	0.324	±	1.020	-0.029	±	0.885	0.253	±	0.022
H2-79	SPLFGH	0.172	±	0.004	0.302	±	0.412	0.486	±	1.805	1.992	±	0.688
H2-44	SPLFHq	0.091	±	0.005	0.238	±	0.220	1.456	±	0.808	1.247	±	0.201
H2-82	SPTFNI	0.219	±	0.013	0.237	±	0.436	-0.054	±	1.310	1.132	±	0.453
H2-74	SPLYNV	0.991	±	0.137	0.225	±	0.123	-1.242	±	2.404	0.289	±	0.043
H2-30	SPLTGF	0.160	±	0.006	0.192	±	0.368	0.769	±	0.870	0.400	±	0.082
H2-78	SPIFNS	0.268	±	0.017	0.173	±	1.242	-12.073	±	10.255	0.437	±	0.038
H2-184	SPLYqI	0.153	±	0.014	0.114	±	1.088	-0.781	±	0.932	0.511	±	0.064
H2-16	TPMFNS	0.229	±	0.002	0.085	±	0.190	0.277	±	0.500	0.573	±	0.143
H2-60	SPMYHL	0.057	±	0.005	0.083	±	0.815	0.714	±	0.890	0.890	±	0.144
H2-2	SPLFNN	0.252	±	0.006	0.019	±	0.136	0.381	±	0.330	1.127	±	0.261
H2-4	SPMFGF	0.183	±	0.001	-0.022	±	0.092	0.284	±	0.435	0.713	±	0.169
H2-182	SPMFQS	0.131	±	0.011	-0.066	±	1.286	0.924	±	0.862	0.966	±	0.113
H2-121	SPLFNI	0.261	±	0.002	-0.092	±	0.941	1.284	±	1.749	1.366	±	0.067
H2-17	SPSFQR	0.197	±	0.003	-0.104	±	0.084	-0.422	±	0.572	0.137	±	0.031
H2-58	SPLFDY	0.084	±	0.011	-0.108	±	0.460	-1.258	±	1.950	1.986	±	0.402
H2-12	SPLLqY	0.161	±	0.001	-0.157	±	0.219	-0.765	±	0.522	0.196	±	0.052
H2-13	SPYFSL	0.177	±	0.002	-0.170	±	0.143	-0.543	±	0.680	0.195	±	0.043
H2-179	SPLFNI	0.133	±	0.003	-0.211	±	0.473	1.381	±	1.139	1.909	±	0.162
H2-80	SPMFAq	0.142	±	0.003	-0.212	±	0.446	0.691	±	1.625	0.666	±	0.242
H2-185	SMPFNI	0.263	±	0.053	-0.238	±	0.624	-0.747	±	0.805	0.429	±	0.094
H2-176	SLMFNS	0.098	±	0.002	-0.356	±	1.123	0.172	±	1.482	0.226	±	0.019
H2-177	TPMFKq	0.082	±	0.003	-0.525	±	1.705	-0.650	±	2.217	0.240	±	0.022
H2-180	SPLLNY	0.225	±	0.019	-0.545	±	0.894	-0.144	±	0.371	0.676	±	0.116
H2-72	SPAFGQ	0.135	±	0.001	-0.636	±	1.512	-7.094	±	5.400	1.589	±	0.294
H2-186	SPLFRV	0.079	±	0.004	-0.643	±	2.342	-2.191	±	2.216	0.194	±	0.018
H2-170	APMFQL	0.073	±	0.002	-0.764	±	1.767	0.147	±	1.764	0.435	±	0.039
H2-133	SPLFNA	0.604	±	0.031	-0.810	±	0.626	0.403	±	0.572	0.357	±	0.031
H2-171	SPLLGE	0.159	±	0.018	-0.823	±	0.956	-0.465	±	0.942	0.086	±	0.012
H2-125	SPLFNL	0.224	±	0.004	-0.861	±	1.303	1.752	±	2.298	1.449	±	0.046
H2-187	SPMFNS	0.124	±	0.018	-0.942	±	1.396	0.851	±	1.455	1.558	±	0.265
H2-132	SPAFNI	0.511	±	0.044	-1.042	±	0.827	0.089	±	0.181	0.328	±	0.034
H2-123	SPLFNI	0.126	±	0.027	-1.091	±	3.540	1.263	±	1.840	2.384	±	0.508
H2-172	SPMFMF	0.101	±	0.004	-1.234	±	1.529	0.253	±	2.524	0.410	±	0.039
H2-90	SPIFGI	0.129	±	0.008	-1.715	±	1.777	-7.885	±	10.835	1.136	±	0.123
H2-105	SPYFNL	0.171	±	0.016	-1.812	±	1.937	-0.001	±	1.361	1.090	±	0.218
H2-120	SPLFNV	0.165	±	0.004	-1.830	±	1.919	1.628	±	2.166	1.823	±	0.152

H2-127	NTMFNV	0.240	±	0.005	-2.149	±	1.806	0.053	±	0.623	1.069	±	0.036
H2-100	SPMFNL	0.120	±	0.007	-2.181	±	2.197	2.426	±	3.204	2.158	±	0.551
H2-85	SPMYHL	0.320	±	0.029	-2.329	±	1.811	-0.695	±	0.942	0.225	±	0.093
H2-110	SPLFDQ	0.183	±	0.020	-2.378	±	2.106	0.113	±	0.947	1.387	±	0.165
H2-128	SPLFNT	0.138	±	0.008	-2.753	±	2.784	0.894	±	1.419	1.984	±	0.141
H2-119	SPMFNT	0.126	±	0.007	-2.884	±	2.655	1.575	±	2.160	2.417	±	0.187
H2-98	SPLLNL	0.103	±	0.008	-3.100	±	4.969	-1.872	±	2.946	0.714	±	0.083
H2-124	SPMFNN	0.119	±	0.002	-3.109	±	2.718	2.087	±	2.779	2.446	±	0.078
H2-83	SPMFNF	0.092	±	0.008	-3.154	±	4.603	1.080	±	2.031	2.055	±	0.228
H2-106	SPSFGF	0.091	±	0.005	-3.201	±	6.161	-1.062	±	2.031	0.608	±	0.131
H2-91	SPMYNV	0.106	±	0.010	-3.292	±	4.108	-1.165	±	3.508	1.282	±	0.215
H2-111	SPLFQI	0.110	±	0.004	-3.342	±	2.891	1.854	±	2.534	2.252	±	0.111
H2-84	SPLFHN	0.212	±	0.017	-3.360	±	2.596	-1.000	±	1.573	0.597	±	0.157
H2-108	SPAFGF	0.090	±	0.005	-4.013	±	3.952	0.342	±	2.220	1.303	±	0.099
H2-122	TPLFNq	0.070	±	0.000	-4.596	±	7.489	-2.750	±	4.135	0.961	±	0.093
H2-89	APLYNL	0.086	±	0.002	-4.820	±	4.102	-13.941	±	18.973	0.526	±	0.021
H2-118	SPMFqQ	0.088	±	0.001	-5.237	±	4.504	1.725	±	2.472	2.324	±	0.128
H2-130	APAFNV	0.110	±	0.005	-5.264	±	4.432	-1.916	±	3.230	1.730	±	0.115
H2-112	SPMLNY	0.087	±	0.010	-6.055	±	4.866	-0.562	±	1.895	1.789	±	0.218
H2-126	SPMFAF	0.095	±	0.006	-6.337	±	5.253	0.714	±	1.460	0.892	±	0.068
H2-131	APAFML	0.116	±	0.015	-6.341	±	4.943	-0.039	±	1.050	0.150	±	0.024
H2-109	TPMYQq	0.082	±	0.005	-6.440	±	5.585	-1.845	±	2.859	0.822	±	0.060
H2-95	APAFQq	0.072	±	0.001	-6.569	±	5.197	0.025	±	1.952	0.436	±	0.073
H2-115	SPMFMQ	0.069	±	0.001	-6.643	±	7.052	-0.437	±	1.156	1.311	±	0.045
H2-103	SPLFqQ	0.066	±	0.003	-6.733	±	5.241	1.245	±	4.539	1.229	±	0.093
H2-96	SPMLEL	0.068	±	0.001	-6.741	±	5.562	0.977	±	2.446	0.460	±	0.111
H2-94	SPIFqH	0.079	±	0.001	-6.790	±	5.483	-0.037	±	1.978	0.522	±	0.090
H2-129	SPLFNW	0.079	±	0.004	-7.063	±	5.630	-0.250	±	1.363	0.781	±	0.066
H2-101	TPLFqN	0.080	±	0.003	-7.347	±	6.134	0.079	±	1.102	0.583	±	0.070
H2-93	SPqFqT	0.068	±	0.002	-7.480	±	6.063	-0.740	±	2.630	0.394	±	0.046
H2-116	TTMFNT	0.073	±	0.000	-8.113	±	6.619	-0.922	±	1.471	0.285	±	0.014
H2-88	SPMYQV	0.092	±	0.013	-9.705	±	7.650	-5.664	±	7.483	0.278	±	0.080

Supplementary Table S3. Data associated with Figure 2 for the scFv variants selected from the F10-CDRH1 library. The 1st column from left shows the name assigned to the scFv variants; the 2nd column shows the sequence, where 'q' represents expressed 'Gln' encoded by the TAG amber codon; the 3rd column shows the concentration of phage-free soluble scFv in culture supernatant; 4th~5th columns show the normalized neutralizing potency against BS/07 H1N1 and CA/09 H1N1 virus respectively; the 6th column shows the normalized binding affinity to BS/07 H1 HA. Three independent measurements were carried out to derive each averaged measurement and standard deviation for each of the scFv variants.

scFv	sequence	concentration (μg/ml)			normalized neutralizing potency against BS/07 H1N1			normalized neutralizing potency against CA/09 H1N1			normalized binding affinity to BS/07 H1N1		
F10	SSEVTFSSF	1.347	±	0.109	1.000			1.000			1.000		
H1-62	YNTAMFPFY	0.112	±	0.008	7.476	±	6.435	3.365	±	2.008	2.628	±	0.378
H3-141	YDTMSFMFY	0.190	±	0.001	2.782	±	1.039	0.742	±	1.201	2.728	±	0.941
H3-132	SNEQLFqHF	0.238	±	0.052	2.172	±	0.967	1.701	±	0.731	1.701	±	0.704
H1-74	WSGLFFSAN	0.098	±	0.020	2.111	±	1.859	1.274	±	1.219	1.047	±	0.394
H1-82	NDRIDFPLT	0.089	±	0.009	1.380	±	1.559	1.494	±	1.345	2.662	±	0.365
H1-81	NTTNILMSD	0.080	±	0.002	1.337	±	1.975	2.470	±	1.794	0.951	±	0.087
H1-80	VIDVMWqSF	0.088	±	0.003	1.234	±	1.535	0.768	±	2.258	1.092	±	0.173
H1-61	VqDLTFQSK	0.192	±	0.007	1.143	±	1.464	-0.597	±	1.056	0.741	±	0.378
H1-83	TRNDFLYWq	0.164	±	0.013	1.088	±	1.090	0.380	±	1.816	1.302	±	0.223
H3-136	SNqISFTSH	0.275	±	0.007	1.087	±	0.467	0.397	±	0.814	1.289	±	0.464
H3-139	DYSYSYGqF	0.288	±	0.028	1.075	±	0.467	0.615	±	0.728	1.373	±	0.492
H1-16	VDDILFPLR	0.228	±	0.010	0.966	±	0.217	-0.024	±	0.658	0.842	±	0.170
H1-5	NNVWEPIPH	0.135	±	0.031	0.904	±	0.318	-0.636	±	0.935	1.262	±	0.383
H1-52	STGqLVYSN	0.232	±	0.013	0.836	±	0.950	0.653	±	0.631	1.491	±	0.180
H3-131	NLqDYVGAN	0.171	±	0.009	0.791	±	0.390	0.897	±	1.335	1.093	±	0.386
H1-2	NKEALILTN	0.113	±	0.010	0.787	±	0.289	-1.339	±	1.849	0.698	±	0.149
H1-98	STDIqFPYH	0.236	±	0.015	0.723	±	0.432	0.318	±	0.418	0.274	±	0.044
H3-130	NPHITWqSH	0.163	±	0.002	0.668	±	0.572	1.126	±	1.115	1.346	±	0.467
H1-86	QYTPVFPLN	0.211	±	0.015	0.663	±	0.876	-0.513	±	1.347	1.216	±	0.133
H3-135	NLqDYVGAN	0.172	±	0.002	0.644	±	0.721	0.736	±	1.409	0.942	±	0.348
H1-67	STKNDFPYY	0.111	±	0.008	0.636	±	1.067	-0.993	±	2.450	0.178	±	0.111
H1-23	NDNITWMLN	0.370	±	0.007	0.533	±	0.143	0.115	±	0.286	0.529	±	0.107
H1-24	SHYPLWMYY	0.392	±	0.005	0.530	±	0.126	0.844	±	0.401	0.513	±	0.101
H1-7	TLNVNLYSH	0.078	±	0.000	0.518	±	0.372	-2.227	±	2.643	0.596	±	0.124
H1-53	qPNEIILSN	0.166	±	0.007	0.509	±	0.593	0.568	±	1.479	2.055	±	0.198
H2-154	SLYPFSqFY	0.146	±	0.001	0.489	±	0.998	1.485	±	1.073	1.175	±	0.101
H1-18	TWGQLFHFG	0.258	±	0.013	0.445	±	0.168	0.259	±	0.482	0.563	±	0.115

H1-33	WqKLAWSMS	0.266	±	0.040	0.438	±	0.205	-0.324	±	0.355	0.504	±	0.124
H1-15	TTEGPLWTQ	0.271	±	0.006	0.436	±	0.109	-0.499	±	0.615	0.424	±	0.084
H1-59	LNGDYLFFq	0.204	±	0.009	0.433	±	0.635	-0.303	±	0.464	0.282	±	0.160
H1-113	DPSqTISYG	0.207	±	0.002	0.423	±	0.363	0.257	±	0.333	0.462	±	0.080
H1-66	HENTYIFAN	0.086	±	0.002	0.420	±	1.730	1.029	±	2.141	0.129	±	0.016
H3-129	NYNTSWWAN	0.177	±	0.005	0.372	±	0.519	0.695	±	1.309	0.316	±	0.110
H1-84	NYqDLLFTN	0.150	±	0.026	0.365	±	0.495	-0.566	±	0.945	1.224	±	0.253
H1-29	DPHTLMMTN	0.225	±	0.004	0.365	±	0.110	-0.380	±	0.456	0.642	±	0.127
H1-60	VVHDIAFFN	0.108	±	0.004	0.360	±	0.927	-0.090	±	1.734	0.752	±	0.356
H1-79	SNETQWYAR	0.069	±	0.003	0.360	±	1.223	-0.874	±	4.190	1.435	±	0.145
H1-68	FNNTHLLTH	0.111	±	0.013	0.291	±	0.978	-0.166	±	1.103	0.207	±	0.117
H1-25	EDSLYFPYY	0.173	±	0.009	0.275	±	0.204	-0.137	±	0.744	0.907	±	0.187
H1-14	EPNTLWFWA	0.204	±	0.004	0.263	±	0.124	-0.289	±	0.336	0.079	±	0.016
H1-20	MSNDLFMHN	0.361	±	0.011	0.260	±	0.079	-0.286	±	0.459	0.331	±	0.066
H1-65	SDSYDWSFR	0.182	±	0.003	0.255	±	0.653	0.471	±	0.566	0.308	±	0.328
H1-63	IDTNDWPFH	0.222	±	0.005	0.253	±	0.772	-0.248	±	0.684	0.585	±	0.196
H2-149	qTKFPLHFG	0.069	±	0.001	0.246	±	0.911	0.939	±	3.516	0.731	±	0.063
H1-28	VqDITMPLR	0.419	±	0.032	0.233	±	0.075	-0.349	±	0.406	0.206	±	0.044
H1-9	WDNWRWIMN	0.189	±	0.022	0.226	±	0.117	-0.587	±	0.911	0.666	±	0.154
L2-109	DqQNLLYSH	0.196	±	0.006	0.200	±	0.297	-0.354	±	1.022	2.002	±	0.694
H1-10	TTDPLLNWN	0.243	±	0.008	0.194	±	0.075	-0.373	±	0.814	0.218	±	0.044
H1-26	NDEILNTYH	0.233	±	0.011	0.182	±	0.123	-0.874	±	0.965	0.218	±	0.046
H1-31	TTHSFFYAN	0.254	±	0.011	0.179	±	0.124	-0.826	±	0.586	0.360	±	0.075
L2-125	ANYTqYYAY	0.573	±	0.013	0.145	±	0.163	-0.174	±	0.315	0.516	±	0.181
H3-140	EYTTMYMSH	0.180	±	0.012	0.114	±	0.108	0.577	±	1.141	0.188	±	0.070
H1-58	TTGLTFYHN	0.110	±	0.002	0.109	±	0.654	-1.830	±	1.798	0.253	±	0.228
H1-17	VSGSTWYFN	0.424	±	0.024	0.043	±	0.070	-0.388	±	0.387	0.180	±	0.037
H1-64	QRNFDMFWN	0.096	±	0.002	0.030	±	0.578	0.458	±	0.694	0.346	±	0.174
H1-99	SNqPTMMSY	0.184	±	0.004	0.005	±	0.631	0.093	±	0.415	0.584	±	0.101
H2-164	VASPNFqFY	0.300	±	0.011	0.002	±	0.725	0.438	±	0.390	0.875	±	0.079
H3-133	EYTTMYMSH	0.157	±	0.007	-0.034	±	0.383	0.441	±	1.323	0.194	±	0.069
H1-88	TLNqLVYTN	0.117	±	0.001	-0.035	±	0.774	-0.618	±	0.669	0.521	±	0.071
H1-1	MDLNTIMST	0.088	±	0.007	-0.050	±	0.129	-0.823	±	1.447	0.090	±	0.019
L1-33	IRqRRIMGq	0.149	±	0.003	-0.052	±	0.416	0.398	±	1.160	0.409	±	0.141
H1-37	HDSVDFASY	0.170	±	0.002	-0.130	±	1.346	0.657	±	3.780	4.617	±	0.866
H3-128	NYNTSWWAN	0.171	±	0.013	-0.193	±	0.782	0.353	±	1.461	0.367	±	0.131
H1-104	VAKYqYqPI	0.155	±	0.002	-0.233	±	0.703	-0.042	±	0.460	0.550	±	0.126
H3-134	LADAqMMSN	0.236	±	0.003	-0.251	±	0.388	0.269	±	1.063	0.401	±	0.139
H1-46	DqQNLLYSH	0.130	±	0.013	-0.261	±	0.918	-0.311	±	1.267	0.796	±	0.107
H1-111	AHWTFWPqF	0.139	±	0.003	-0.277	±	0.661	-0.012	±	0.512	0.418	±	0.055
H2-160	qVSDILYYN	0.083	±	0.009	-0.288	±	1.485	1.681	±	1.876	0.726	±	0.101
H1-102	SFEGPFMSK	0.121	±	0.001	-0.292	±	0.799	-0.063	±	0.948	0.452	±	0.058
H1-109	YYqDALGAH	0.164	±	0.011	-0.414	±	0.442	-0.152	±	0.233	0.470	±	0.084
H1-103	QLqRLTTKP	0.117	±	0.000	-0.451	±	0.844	-0.246	±	0.891	0.249	±	0.038
H1-108	qFDNDWqNW	0.175	±	0.004	-0.481	±	0.659	0.049	±	0.808	0.739	±	0.107
H1-110	LMqPAFDTG	0.125	±	0.003	-0.526	±	0.755	-0.110	±	0.573	0.199	±	0.032
H1-100	VNNLLWSqN	0.146	±	0.001	-0.621	±	0.604	-0.313	±	0.572	0.359	±	0.055
H2-156	SSqLIWDVY	0.065	±	0.004	-0.785	±	3.061	-0.141	±	2.684	0.566	±	0.069
H1-36	SHYLNYqHF	0.249	±	0.003	-0.799	±	0.407	-1.263	±	2.663	1.803	±	0.337

H1-38	qVWGqqNVI	0.134	±	0.000	-0.945	±	0.442	0.456	±	5.289	0.901	±	0.172
H2-71	TRLqCPVMD	0.137	±	0.000	-1.913	±	1.558	-2.997	±	3.493	0.672	±	0.125

Supplementary Table S4. Data associated with Figure 3 for the scFv variants selected from the F10-CDRH3 library. The 1st column from left shows the name assigned to the scFv variants; the 2nd column shows the sequence, where 'q' represents expressed 'Gln' encoded by the TAG amber codon; the 3rd column shows the concentration of phage-free soluble scFv in culture supernatant; 4th~5th columns show the normalized neutralizing potency against BS/07 H1N1 and CA/09 H1N1 virus respectively; the 6th column shows the normalized binding affinity to BS/07 H1 HA. Three independent measurements were carried out to derive each averaged measurement and standard deviation for each of the scFv variants.

scFv	sequence	concentration (μg/ml)			normalized neutralizing potency against BS/07 H1N1			normalized neutralizing potency against CA/09 H1N1			normalized binding affinity to BS/07 H1N1		
F10	PSYICSGGTC	1.347	±	0.109	1.000			1.000			1.000		
H3-268	PQYAIPLAGG	0.075	±	0.004	8.021	±	6.038	8.040	±	10.606	2.694	±	0.392
H3-48	PSYNATISKG	0.093	±	0.004	7.815	±	7.027	6.129	±	2.363	0.339	±	0.040
H3-228	PSYIANN---	0.207	±	0.006	2.716	±	0.204	1.121	±	0.834	2.453	±	0.090
H3-188	PqYQTIKGLG	0.033	±	0.004	2.193	±	2.729	5.330	±	3.142	12.603	±	1.531
H3-172	NTAFIAHMNS	0.164	±	0.044	2.153	±	1.121	-0.088	±	1.533	1.782	±	0.479
H3-49	PWYTPANSLS	0.151	±	0.005	1.966	±	1.815	1.471	±	1.449	0.129	±	0.014
H3-206	PSYISSq---	0.148	±	0.008	1.875	±	0.525	0.337	±	0.820	2.778	±	0.166
H3-61	PTYNTTMSNG	0.132	±	0.013	1.708	±	0.397	1.549	±	0.944	1.811	±	0.399
H3-205	PTFSPTNADA	0.223	±	0.030	1.691	±	0.503	-0.216	±	1.284	2.367	±	0.324
H3-182	PTYHVMPHVG	0.334	±	0.008	1.654	±	0.170	1.468	±	0.221	1.882	±	0.061
H3-219	LSFTCSTACH	0.301	±	0.041	1.480	±	0.283	1.783	±	0.351	1.679	±	0.234
H3-186	PTYITGSDGA	0.417	±	0.031	1.460	±	0.135	0.664	±	0.229	1.520	±	0.120
H3-187	PGYKMGTLSG	0.175	±	0.010	1.431	±	0.391	0.547	±	1.387	3.200	±	0.215
H3-50	PTYLVQPSLL	0.329	±	0.009	1.425	±	1.270	0.804	±	0.784	0.340	±	0.042
H3-106	PNFSPLSPQL	0.314	±	0.071	1.407	±	1.397	2.358	±	1.049	0.365	±	0.088
H3-229	NTYNLQMPKQ	0.318	±	0.013	1.315	±	0.222	1.203	±	0.194	1.608	±	0.083
H3-177	PLYNTALAQG	0.256	±	0.009	1.313	±	0.354	0.051	±	0.749	2.153	±	0.088
H3-181	PGYAIGNTNH	0.437	±	0.008	1.234	±	0.218	0.551	±	0.375	1.368	±	0.042
H3-204	NTALVSNRGA	0.138	±	0.001	1.149	±	1.001	-0.834	±	1.758	0.764	±	0.015
H3-165	PTYSQNA---	1.304	±	0.117	1.123	±	0.222	-1.350	±	1.916	0.621	±	0.074
H1-40	PGYLAHSRDL	0.155	±	0.023	1.052	±	0.663	-5.030	±	5.572	7.465	±	1.756
H3-47	PSYTINKNTF	1.090	±	0.066	1.017	±	0.856	0.628	±	0.281	0.382	±	0.040
H3-213	NTYNSMGENT	0.376	±	0.026	0.973	±	0.165	-0.315	±	0.499	1.125	±	0.082
H3-194	PTYNCANTSC	0.622	±	0.055	0.969	±	0.102	0.846	±	0.143	0.790	±	0.074
H3-223	NLYLISLATA	0.188	±	0.006	0.956	±	0.624	-1.215	±	1.163	0.871	±	0.052
H3-231	PSYVVGVKEN	0.494	±	0.023	0.955	±	0.078	0.158	±	0.113	0.984	±	0.049
H3-3	PTYNFGSDSF	0.155	±	0.004	0.934	±	0.345	0.593	±	0.712	1.441	±	0.190
H3-173	PTYTMSSTNS	0.657	±	0.029	0.902	±	0.067	0.364	±	0.219	0.914	±	0.046
H3-10	TGYTPFGPRM	0.135	±	0.004	0.883	±	0.472	1.419	±	0.821	1.507	±	0.199

H3-208	qMSSVSYKGA	0.164	±	0.016	0.851	±	0.452	-0.678	±	1.449	0.703	±	0.070
H3-105	NCLYMNTNLC	0.433	±	0.115	0.850	±	0.771	1.038	±	0.695	0.090	±	0.025
H3-180	PNYSAINASQ	0.397	±	0.005	0.839	±	0.393	-0.164	±	0.319	1.295	±	0.031
H3-35	PYFSMTNHQP	0.247	±	0.007	0.825	±	1.179	0.416	±	0.204	0.201	±	0.027
H3-214	RGYNVGPLSS	0.422	±	0.035	0.825	±	0.283	0.521	±	0.274	1.201	±	0.103
H3-32	PGYTLSNYSN	0.182	±	0.023	0.803	±	0.569	1.333	±	0.345	1.961	±	0.352
H3-248	PSYAVNKEGY	0.485	±	0.095	0.795	±	0.737	0.695	±	0.935	0.698	±	0.138
H3-220	qLIqqMFNRS	0.082	±	0.001	0.792	±	1.344	-2.125	±	3.704	0.141	±	0.008
H3-51	NTTYLAHLMS	0.151	±	0.012	0.742	±	1.151	0.901	±	0.831	0.179	±	0.022
H3-221	PSYAIMNDSL	0.465	±	0.013	0.739	±	0.152	-0.508	±	0.374	0.909	±	0.031
H3-199	QTYNIqTSKS	0.176	±	0.008	0.676	±	0.616	-1.394	±	3.016	1.709	±	0.087
H3-202	PTYAVSTQPI	0.349	±	0.030	0.653	±	0.343	-0.087	±	0.669	0.977	±	0.088
H3-65	SqSYHPGPDL	0.139	±	0.031	0.651	±	0.374	-0.704	±	0.781	1.297	±	0.385
H3-210	QNYLPGAGLH	0.768	±	0.049	0.621	±	0.078	-0.075	±	0.161	0.548	±	0.037
H3-183	PGYNVKIGTA	0.951	±	0.035	0.572	±	0.054	0.462	±	0.081	0.647	±	0.027
H3-59	PTYNLTAPGN	0.360	±	0.039	0.554	±	0.137	-0.063	±	0.287	0.647	±	0.145
H3-207	PGYSPSGLTT	0.476	±	0.017	0.533	±	0.147	-0.159	±	0.400	0.826	±	0.038
H3-19	PNFLPASDEI	0.197	±	0.003	0.524	±	0.392	-0.116	±	0.841	0.663	±	0.086
H3-234	PHYNPADERI	0.186	±	0.037	0.500	±	0.247	-1.015	±	0.477	0.815	±	0.163
H3-70	PHYRCNNKDC	0.477	±	0.014	0.495	±	0.103	0.559	±	0.264	0.563	±	0.112
H3-95	NTYVLSANNQ	0.439	±	0.109	0.494	±	0.162	0.481	±	0.291	0.482	±	0.153
H3-99	PTYRTDHEDA	0.389	±	0.024	0.490	±	0.110	0.615	±	0.310	0.623	±	0.128
H3-200	TTAYTGTRDS	0.301	±	0.045	0.473	±	0.288	-0.209	±	0.417	0.076	±	0.012
H3-29	PDYASTMTGG	0.242	±	0.029	0.462	±	0.177	0.264	±	0.385	1.279	±	0.227
H3-79	PSYICSKNC-	0.499	±	0.061	0.457	±	0.111	0.726	±	0.353	0.513	±	0.119
H3-54	PNYIPALPQV	0.100	±	0.000	0.445	±	0.387	1.740	±	0.825	1.706	±	0.336
H3-189	PSFNVRSSDV	0.576	±	0.037	0.444	±	0.349	-0.093	±	0.247	0.687	±	0.048
H3-83	qEYIPTASNG	0.154	±	0.028	0.422	±	0.227	-0.825	±	1.409	0.682	±	0.185
H3-57	HGQYYSITND	0.178	±	0.001	0.418	±	0.232	-1.162	±	1.341	0.153	±	0.030
H3-211	RSYYGTDTSI	0.961	±	0.093	0.405	±	0.063	0.424	±	0.101	0.501	±	0.049
H3-163	PHYNPADERI	0.222	±	0.009	0.404	±	0.430	0.490	±	0.723	1.149	±	0.398
H3-243	PGYNLRTSSA	1.249	±	0.151	0.395	±	0.298	0.519	±	0.680	0.235	±	0.030
H3-196	PSYSVALESM	0.891	±	0.055	0.393	±	0.145	0.154	±	0.176	0.513	±	0.033
H3-55	PAYNTDSGSS	0.243	±	0.023	0.388	±	0.329	-0.423	±	0.268	0.748	±	0.163
H3-192	PAYKINSTTM	1.579	±	0.000	0.387	±	0.021	0.187	±	0.038	0.338	±	0.010
H3-102	RYYSAASRDD	0.342	±	0.021	0.383	±	0.538	-0.041	±	0.487	0.878	±	0.093
H3-216	PYYLIRNDST	0.407	±	0.004	0.379	±	0.044	-0.332	±	0.426	0.570	±	0.022
H3-118	YSYSPVYNPS	0.851	±	0.150	0.375	±	1.081	0.088	±	0.733	0.621	±	0.161
H3-168	qTYHCKERCS	0.531	±	0.044	0.366	±	0.749	-5.048	±	6.026	0.301	±	0.031
H3-8	PTFYIGPTTA	0.079	±	0.002	0.351	±	0.728	2.226	±	0.564	2.274	±	0.304
H3-225	PSYNILDRML	0.675	±	0.022	0.347	±	0.137	-0.560	±	0.389	0.540	±	0.020
H3-201	PTFTPSIHMS	0.259	±	0.005	0.345	±	0.352	-0.220	±	1.115	0.702	±	0.024
H3-233	PSYFIqPMNP	0.357	±	0.035	0.331	±	0.165	-0.624	±	0.612	0.726	±	0.074
H3-111	LRYNTSIYED	0.937	±	0.060	0.330	±	0.615	0.331	±	0.398	1.300	±	0.255
H3-226	PYYTAWHDHS	1.056	±	0.074	0.329	±	0.056	-0.150	±	0.252	0.417	±	0.035
H3-191	PGYIGPYNGS	1.497	±	0.081	0.329	±	0.046	0.168	±	0.127	0.326	±	0.019
H3-197	PTYNLFSINS	1.523	±	0.035	0.327	±	0.032	0.099	±	0.168	0.343	±	0.011
H3-69	PNYSASRHIG	0.450	±	0.061	0.318	±	0.130	-0.045	±	0.229	0.444	±	0.106
H3-185	PEYTPTVTEL	0.444	±	0.045	0.315	±	0.131	-0.009	±	0.497	0.871	±	0.096
H3-110	NRDYNISNTI	1.762	±	0.061	0.303	±	0.572	-0.358	±	0.500	0.153	±	0.038
H3-161	NVYKPGTPDM	0.335	±	0.012	0.302	±	0.264	0.457	±	0.563	1.227	±	0.425
H3-16	PTYSSNPGES	0.302	±	0.029	0.302	±	0.285	0.136	±	0.172	0.960	±	0.155
H3-162	PSYFIqPMNP	0.377	±	0.018	0.293	±	0.161	0.207	±	0.525	0.949	±	0.344

H3-184	PYYLPWDVPV	1.539	±	0.009	0.291	±	0.042	0.016	±	0.137	0.367	±	0.008
H3-256	PSYLINSNTM	2.461	±	0.139	0.285	±	0.212	0.133	±	0.176	0.137	±	0.009
H3-90	NSYSVLHRSA	0.819	±	0.024	0.280	±	0.061	0.437	±	0.205	0.252	±	0.050
H3-215	DQYTLWDTGS	0.350	±	0.025	0.266	±	0.158	-0.443	±	0.537	0.037	±	0.003
H3-63	PTYYTVPDQI	0.328	±	0.046	0.261	±	0.200	0.069	±	0.280	0.247	±	0.060
H3-190	PGYLITSHFD	1.421	±	0.084	0.260	±	0.083	-0.071	±	0.171	0.319	±	0.020
H3-107	NQQYMNGTNN	0.384	±	0.013	0.258	±	0.360	0.082	±	0.716	0.973	±	0.087
H3-13	PTYYqTYGGS	0.087	±	0.004	0.254	±	0.493	-0.279	±	1.223	0.665	±	0.096
H3-115	PTYRIISRQI	0.272	±	0.021	0.249	±	0.610	2.015	±	1.570	0.328	±	0.067
H3-227	DTQYVSNATR	0.222	±	0.006	0.244	±	0.334	-1.305	±	0.795	1.222	±	0.045
H3-170	PTYMIGRDTN	9.019	±	0.180	0.240	±	0.026	-0.236	±	0.376	0.185	±	0.011
H3-230	SGYNLVHPKS	0.597	±	0.053	0.231	±	0.164	-0.379	±	0.194	0.482	±	0.044
H3-108	IGGYLPSLNP	3.132	±	0.121	0.231	±	0.087	0.013	±	0.144	0.282	±	0.054
H3-58	PYYTPLILGS	0.536	±	0.040	0.219	±	0.117	0.125	±	0.210	0.370	±	0.078
H3-85	NNTYFTLDNS	0.498	±	0.050	0.215	±	0.094	-0.369	±	0.451	0.371	±	0.086
H3-6	PDYTPIYNNV	0.208	±	0.016	0.215	±	0.205	-0.261	±	0.821	0.869	±	0.130
H3-193	PSYDIKPRTD	1.071	±	0.063	0.213	±	0.092	0.014	±	0.192	0.324	±	0.022
H3-96	NTFALPLGSM	0.938	±	0.112	0.211	±	0.056	0.383	±	0.184	0.217	±	0.050
H3-179	PYYLLQDSNS	0.661	±	0.015	0.186	±	0.147	-0.244	±	0.561	0.266	±	0.012
H3-175	PTFTINTPQQ	0.973	±	0.033	0.182	±	0.067	0.049	±	0.159	0.453	±	0.018
H3-21	PNYYTNNGPD	0.215	±	0.040	0.181	±	0.350	-0.084	±	0.839	0.170	±	0.039
H3-112	NAYSTLIPTS	1.513	±	0.091	0.172	±	0.148	0.063	±	0.280	0.542	±	0.115
H3-89	QGFSPFSSHY	0.794	±	0.026	0.171	±	0.048	0.335	±	0.164	0.209	±	0.042
H3-195	SNYSPLMSNP	1.561	±	0.007	0.159	±	0.074	0.075	±	0.090	0.245	±	0.005
H3-167	PYAFANTADT	6.559	±	0.155	0.159	±	0.047	-0.368	±	0.599	0.106	±	0.007
H3-109	PSYWVEPNDF	7.310	±	0.432	0.141	±	0.037	-0.047	±	0.140	0.108	±	0.021
H3-60	PMALITSPDT	0.501	±	0.049	0.139	±	0.066	-0.314	±	0.311	0.160	±	0.038
H3-87	NEYLSISTSQ	0.309	±	0.052	0.139	±	0.097	-0.727	±	0.683	0.309	±	0.080
H3-174	PTLYLRSAHS	0.815	±	0.040	0.136	±	0.159	-0.035	±	0.182	0.192	±	0.012
H3-77	PSWRCKSSTC	0.264	±	0.031	0.136	±	0.111	-0.661	±	0.690	0.256	±	0.059
H3-53	PAYAVFPDIL	0.606	±	0.062	0.127	±	0.057	0.020	±	0.120	0.263	±	0.058
H3-82	PDYIANSLSM	0.492	±	0.032	0.127	±	0.046	-0.348	±	0.377	0.160	±	0.034
H3-122	PqYTPIDTMV	0.297	±	0.024	0.124	±	0.532	2.801	±	1.476	2.924	±	0.716
H3-113	QNYNANWPSN	1.680	±	0.060	0.118	±	0.206	0.166	±	0.230	0.521	±	0.125
H3-125	NTYTAAQPNq	0.392	±	0.016	0.118	±	0.615	1.221	±	1.267	3.537	±	0.680
H3-64	PFYKLTNNIS	0.783	±	0.059	0.113	±	0.056	-0.181	±	0.218	0.176	±	0.037
H3-42	PHYTVQVGNP	0.176	±	0.007	0.112	±	0.183	-0.201	±	0.449	1.173	±	0.159
H3-92	NKYNTRTSGM	0.710	±	0.032	0.111	±	0.035	0.319	±	0.175	0.199	±	0.040
H3-62	PYYTLSHKMH	0.528	±	0.022	0.100	±	0.122	-0.064	±	0.335	0.204	±	0.041
H3-41	PAYIANHLVA	0.345	±	0.044	0.099	±	0.153	0.052	±	0.219	0.636	±	0.115
H3-88	NHQYLTHNDD	0.631	±	0.074	0.099	±	0.041	-0.201	±	0.345	0.192	±	0.045
H3-71	PNYYANDAMT	0.586	±	0.079	0.098	±	0.059	-0.287	±	0.296	0.208	±	0.050
H3-37	PYHSPHSPNT	0.337	±	0.012	0.086	±	0.203	0.008	±	0.123	0.945	±	0.127
H3-160	PSYFIqPMNP	0.596	±	0.048	0.079	±	0.112	-0.019	±	0.344	0.606	±	0.215
H3-98	PYYMPNVASS	0.448	±	0.016	0.077	±	0.044	-0.644	±	0.549	0.320	±	0.064
H3-158	NVYKPGTPDM	0.256	±	0.035	0.072	±	0.433	0.378	±	0.789	1.625	±	0.601
H3-80	TSYNLNSNTT	0.808	±	0.016	0.065	±	0.030	-0.167	±	0.183	0.095	±	0.019
H3-52	PGFSPSATTL	0.413	±	0.023	0.063	±	0.287	-0.540	±	0.740	0.711	±	0.099
H3-72	TGYSFRNPAS	0.495	±	0.050	0.063	±	0.065	-0.481	±	0.401	0.034	±	0.008
H3-78	PMYSFYGLQA	0.531	±	0.066	0.056	±	0.042	-0.208	±	0.188	0.234	±	0.055
H3-45	PYFDAGNHDT	0.745	±	0.095	0.053	±	0.044	0.046	±	0.149	0.477	±	0.087
H3-103	SNSYHPNDNF	0.347	±	0.026	0.045	±	0.368	0.854	±	0.517	0.388	±	0.043
H3-116	TGYILTDHSS	3.234	±	0.256	0.043	±	0.103	0.030	±	0.093	0.154	±	0.033

H3-176	PRAVLGPHNA	0.773	±	0.054	0.040	±	0.027	-0.183	±	0.262	0.141	±	0.014
H3-86	PELHLGPWNY	0.241	±	0.040	0.031	±	0.072	-0.214	±	0.429	0.095	±	0.025
H3-169	PHYLPSKGLM	14.923	±	0.215	0.008	±	0.024	-0.116	±	0.116	0.099	±	0.007
H3-100	SSYNFNSVHS	1.171	±	0.050	0.007	±	0.012	-0.237	±	0.199	0.017	±	0.003
H3-9	PTYTLNMSKV	0.206	±	0.013	0.006	±	0.075	0.571	±	0.247	0.559	±	0.080
H3-40	PNYIPALPQV	0.095	±	0.004	0.003	±	0.536	-0.211	±	0.325	0.924	±	0.126
H3-124	RAYRLGTPKT	0.291	±	0.025	0.000	±	0.809	0.240	±	1.623	0.274	±	0.182
H3-101	NTYIPGWTTS	0.310	±	0.007	-0.001	±	0.036	0.025	±	0.137	0.466	±	0.092
H3-93	HMYNIENPDS	0.537	±	0.051	-0.001	±	0.037	-0.492	±	0.368	0.135	±	0.030
H3-123	NKFLPESAGS	3.657	±	0.086	-0.004	±	0.022	0.120	±	0.196	0.056	±	0.011
H3-159	PTQWFNQDNQ	0.579	±	0.024	-0.004	±	0.176	0.018	±	0.298	0.445	±	0.158
H3-94	PTYTSSPTSS	0.256	±	0.020	-0.005	±	0.065	0.212	±	0.282	0.526	±	0.111
H3-119	VGYNVMASLS	3.799	±	0.310	-0.012	±	0.109	0.132	±	0.165	0.058	±	0.012
H3-117	PSYLIAYRAN	1.011	±	0.049	-0.025	±	0.395	-0.213	±	0.568	0.464	±	0.089
H3-44	PFSYMPTLEY	0.543	±	0.052	-0.030	±	0.047	-0.250	±	0.152	0.180	±	0.029
H3-24	PSYGPQPTSM	0.301	±	0.046	-0.031	±	0.141	0.081	±	0.165	0.360	±	0.072
H3-120	DHSYLVDPNI	1.937	±	0.265	-0.040	±	0.145	0.153	±	0.330	0.370	±	0.086
H3-238	PTQWFNQDNQ	0.619	±	0.036	-0.053	±	0.211	-0.388	±	0.403	0.341	±	0.026
H3-121	PNYTIDIPSM	1.225	±	0.126	-0.072	±	0.221	0.293	±	0.360	0.528	±	0.112
H3-46	PTYqLTSSNM	0.368	±	0.019	-0.081	±	0.133	-0.317	±	0.521	0.708	±	0.102
H3-150	PGYHTSLASS	0.451	±	0.016	-0.082	±	0.161	0.059	±	0.497	0.630	±	0.218
H1-42	RTYNLGLNND	0.571	±	0.078	-0.091	±	0.359	0.103	±	1.078	2.471	±	0.569
H3-126	NTFNTQNANT	0.720	±	0.077	-0.093	±	0.641	0.232	±	0.607	1.285	±	0.276
H3-36	PKYLASSTAS	0.211	±	0.011	-0.100	±	0.067	0.185	±	0.153	0.557	±	0.081
H3-23	PTYNTSPDNP	0.199	±	0.022	-0.121	±	0.161	-0.207	±	0.300	0.693	±	0.118
H3-7	PAYLSTASSL	0.126	±	0.016	-0.156	±	0.300	-0.200	±	1.063	0.938	±	0.168
H3-104	PGYVTAPTTS	0.159	±	0.015	-0.182	±	0.502	2.073	±	1.297	0.084	±	0.012
H3-39	RSIYPSGPDL	0.245	±	0.027	-0.223	±	0.150	-0.319	±	0.207	0.474	±	0.080
L1-1	RTYDIAGVDL	0.517	±	0.015	-0.240	±	0.489	0.858	±	1.010	0.521	±	0.103
H3-257	PVQHVKNQES	2.069	±	0.255	-0.246	±	0.197	0.015	±	0.051	0.100	±	0.013
H3-43	PSALASTAAG	0.095	±	0.000	-0.256	±	0.119	-0.098	±	1.111	0.699	±	0.095
H3-14	PTFTPIPSNS	0.194	±	0.000	-0.272	±	0.254	-0.544	±	0.399	0.332	±	0.046
H3-15	PTYELRTPAq	0.084	±	0.010	-0.347	±	0.304	0.419	±	0.643	1.201	±	0.214
H3-247	PTFTSFSSMQ	1.199	±	0.179	-0.436	±	0.356	-0.009	±	0.137	0.135	±	0.021
H1-44	KSYNPIANLE	0.232	±	0.014	-0.501	±	0.833	-1.688	±	3.654	3.826	±	0.749
H3-246	NTFSNSDQSM	0.941	±	0.220	-0.516	±	0.434	-0.017	±	0.193	0.169	±	0.040
H3-242	PTYMLVPAMT	1.076	±	0.062	-0.551	±	0.451	0.067	±	0.143	0.189	±	0.018
H3-260	PTYSSSLNDL	1.251	±	0.179	-0.579	±	0.455	-0.199	±	0.373	0.131	±	0.022
H1-45	KSYNPIANLE	0.228	±	0.012	-0.630	±	1.355	-1.970	±	3.513	4.074	±	0.785
H1-41	PTYTSSPTSS	0.319	±	0.028	-0.681	±	0.800	-1.459	±	2.805	3.316	±	0.681
H3-253	PTYSVYTKGH	0.643	±	0.291	-0.745	±	0.733	0.277	±	0.401	0.383	±	0.174
H1-39	PSFILSSAGN	0.178	±	0.002	-0.813	±	0.564	-1.970	±	3.329	4.005	±	0.748
H3-272	PSYTSTASDA	0.457	±	0.018	-1.120	±	0.936	-0.051	±	0.195	0.470	±	0.022
H3-251	NRYTNNLPGA	0.439	±	0.045	-1.182	±	1.111	0.242	±	0.374	0.452	±	0.060
H3-266	PGYALTAINN	0.261	±	0.011	-1.457	±	1.487	0.046	±	0.385	0.705	±	0.072
H3-254	PTAFATYKGL	0.412	±	0.080	-1.611	±	1.348	-0.323	±	0.545	0.189	±	0.039
H1-43	PPYPLPSPRR	0.131	±	0.000	-1.636	±	1.468	-3.414	±	5.126	0.691	±	0.168
H3-245	PQHTAQAVDI	0.426	±	0.070	-1.676	±	1.329	-0.401	±	0.738	0.160	±	0.027
H3-274	PDFMPNSKTI	0.283	±	0.003	-2.011	±	1.503	-0.103	±	0.476	0.798	±	0.162
H3-281	PNYHSTNSEA	0.322	±	0.017	-2.130	±	1.632	-0.226	±	0.524	0.647	±	0.065
H3-275	PYYKGGDWSS	0.271	±	0.009	-2.202	±	1.749	-0.345	±	0.628	0.346	±	0.120
H3-273	PTYISDSSAY	0.313	±	0.002	-2.354	±	1.834	-0.360	±	0.581	0.389	±	0.017
H3-267	PTYTFQRPHS	0.291	±	0.022	-2.400	±	2.093	-0.891	±	1.216	0.321	±	0.061

H3-255	PIYTPIKGGA	0.232	±	0.026	-2.950	±	2.315	-0.232	±	0.830	0.700	±	0.082
H3-250	PEYDINANSY	0.218	±	0.001	-2.965	±	2.442	-0.748	±	1.269	0.388	±	0.022
H3-270	PSFVVNELRP	0.241	±	0.008	-3.024	±	2.310	-0.213	±	0.751	0.140	±	0.017
H3-278	HSGYSPPADP	0.234	±	0.022	-3.516	±	2.735	-1.316	±	1.782	0.640	±	0.067
H3-265	PYAAPGGPDL	0.168	±	0.001	-4.170	±	3.274	-1.190	±	1.603	0.098	±	0.005
H3-282	PTHLVGIPPT	0.192	±	0.004	-4.321	±	3.303	-1.307	±	1.762	0.114	±	0.005
H3-263	PAYNLTGPLS	0.137	±	0.005	-4.692	±	3.664	0.416	±	1.314	1.116	±	0.118
H3-271	PQYTPPLSNS	0.158	±	0.008	-4.805	±	3.660	-0.574	±	1.239	0.497	±	0.044
H3-279	PAYqISGSKS	0.116	±	0.003	-6.822	±	5.158	-1.230	±	1.746	0.858	±	0.061
H3-262	PTFSAQTSDL	0.064	±	0.002	-11.659	±	9.223	-0.598	±	1.706	1.750	±	0.116
H3-277	RTYILTHPLN	0.070	±	0.020	-13.981	±	11.286	-3.164	±	4.431	1.311	±	0.392
H3-283	PSYIVSTRQT	0.062	±	0.002	-14.300	±	10.866	-5.267	±	7.075	0.434	±	0.024
H3-269	MRNYSPNSSG	0.048	±	0.004	-17.961	±	13.563	-8.123	±	10.931	0.247	±	0.086

Supplementary Table S5. Data associated with Figure 6b for the scFv variants selected from the F10-CDRH123 library. The 1st column from left shows the name assigned to the scFv variants; the 2nd~4th columns show respectively the sequences of CDR-H1~H3, where 'q' represents expressed 'Gln' encoded by the TAG amber codon; the 5th column shows the concentration of phage-free soluble scFv in culture supernatant; 6th~7th columns show the normalized neutralizing potency and normalized binding affinity against CA/09 H1N1 virus and CA/09 H1 HA respectively. Three independent measurements were carried out to derive each averaged measurement and standard deviation for each of the scFv variants.

scFv	CDRH1	CDRH2	CDRH3	concentration (μg/ml)			normalized neutralizing potency against CA/09 H1N1			normalized binding affinity against CA/09 H1 HA		
F10	SSEVTFSSF	SPMFGT	PSYICSGGTC	1.174	±	0.043	1.000			1.00		
H123#390	SSEVMFPSF	SPMFqH	PQYMVSG	0.166	±	0.010	3.950	±	0.475	4.344	±	0.409
H123#437	SSEFMFPAF	SPMFDH	PTYLPSG	0.183	±	0.006	3.487	±	0.893	5.158	±	0.475
1101-139	SSEFLFPFF	SPMFSH	PSYRPNA	0.298	±	0.001	3.419	±	1.019	3.290	±	0.494
3rd_1-4-B-02	SSELMFPYF	SPMFNQ	PSYKPIG	0.167	±	0.003	3.350	±	0.862	4.568	±	0.420
1101-150	SSELFFPYF	SPMFSL	PSYRPSH	0.310	±	0.002	3.318	±	1.006	3.559	±	0.764
1026B62	SSELLFPMF	SPMFQV	PSYITQG	0.234	±	0.002	3.316	±	0.484	5.043	±	0.433
H123#456	SSEPqFPFF	SPMFPS	PTYYPSH	0.197	±	0.013	3.134	±	0.827	2.059	±	0.248
3rd_1-4-E-01	SSEYAFPYF	SPMFqY	PSYVPSG	0.179	±	0.004	3.039	±	0.812	1.133	±	0.108
1101-152	SSELAFPFF	SPMFqN	PTYLPRH	0.297	±	0.008	3.025	±	0.976	2.808	±	0.545
1026B52	SSELFFPYF	SPMFSL	PSYRPSH	0.277	±	0.005	2.976	±	0.484	3.765	±	0.327
H123#418	SSEFLFPYF	SPMFqR	PSYLTRS	0.208	±	0.000	2.855	±	0.535	3.392	±	0.267
1101-178	SSELLFPYF	SPMFNQ	PTYSTKG	0.313	±	0.029	2.804	±	1.170	3.903	±	0.736
H123#75	SSEITFPFF	SPMFSq	PTYQPSA	0.446	±	0.007	2.764	±	0.329	3.670	±	0.303
3rd_0.5-0.25-07G	SSELVFPFF	SPMFGq	PTYHPTG	0.374	±	0.006	2.746	±	0.517	2.760	±	0.163
3rd_0.5-1-12B	SSELTFPMF	SPMFEq	PLYVTRG	0.393	±	0.015	2.721	±	0.539	2.479	±	0.366
1101-170	SSELGFPYF	SPMFDF	PTYLPGA	0.350	±	0.019	2.706	±	0.836	2.788	±	0.519
1101-161	SSELNFPFF	SPMFqW	PSYLASA	0.311	±	0.018	2.683	±	0.933	2.605	±	0.487
3rd_0.5-4-09H	SSELVFPLF	SPMFNK	PTYLPRH	0.441	±	0.016	2.664	±	0.561	2.459	±	0.387
1101-168	SSELqFPSF	SPMFSW	PTYVPRG	0.290	±	0.003	2.656	±	1.008	2.875	±	0.490
3rd_0.5-1-11C	SSELqFPHF	SPMFSY	PTYTPRG	0.407	±	0.001	2.651	±	0.467	2.513	±	0.365
2D-0.5-9E	SSELMFPSF	SPMFqA	PTYTPSH	0.239	±	0.032	2.642	±	0.760	3.597	±	0.574
H123#20	SSEqLFPYF	SPMFGL	PEYRPSG	0.441	±	0.008	2.626	±	0.336	2.382	±	0.178
1101-166	SSEMqFPYF	SPMFQH	PRYLPSN	0.342	±	0.002	2.615	±	0.817	0.383	±	0.093
3rd_0.5-4-09E	SSELMFPSF	SPMFRH	PLYMTRG	0.439	±	0.018	2.612	±	0.569	2.391	±	0.361
3rd_0.5-0.5-6C	SSEVMFPFF	SPMFqL	PSYIARG	0.455	±	0.014	2.563	±	0.479	2.121	±	0.193
1026R55	SSEVTFPLF	SPMFqF	PTYITQG	0.292	±	0.003	2.554	±	0.783	2.986	±	0.201
1101-159	SSELLFPYF	SPMFDq	PTYRPSF	0.290	±	0.010	2.549	±	1.150	3.783	±	0.663
1026R54	SSEFLFPFF	SPMFSH	PSYRPNA	0.292	±	0.002	2.546	±	0.796	3.235	±	0.215

3rd_0.5-4-08C	SSELSFPFF	SPMFqH	PEYVYSG	0.398	±	0.016	2.532	±	0.457	2.434	±	0.387
3rd_0.5-0.125-6A	SSEILFPFF	SPMFNq	PTYTPRD	0.461	±	0.009	2.509	±	0.527	2.471	±	0.340
3rd_0.5-0.125-2B	SSEWLFPMF	SPMFqL	PTYRPSL	0.338	±	0.006	2.507	±	0.522	2.500	±	0.236
1101-188	SSELVFPHF	SPMFqL	PWYMPRG	0.352	±	0.038	2.487	±	0.868	2.261	±	0.428
3rd_0.5-4-09G	SSELTFPLF	SPMFqL	PGYIASH	0.375	±	0.019	2.485	±	0.593	2.271	±	0.335
1026B77	SSEMLFPYF	SPMFAT	PTYRPSG	0.327	±	0.013	2.472	±	0.374	3.993	±	0.392
1101-154	SSEIqFPYF	SPMFHL	PTYLASH	0.318	±	0.002	2.470	±	0.887	2.270	±	0.403
3rd_0.5-2-2A	SSELLFPFF	SPMFQS	PSYLPGG	0.451	±	0.019	2.462	±	0.476	2.762	±	0.420
H123#37	SSELTFPSF	SPMFNT	PSYLPSY	0.304	±	0.002	2.454	±	0.485	2.646	±	0.184
3rd_0.5-0.0625-11D	SSEILFPYF	SPMFqA	PTYYANA	0.465	±	0.005	2.454	±	0.455	2.825	±	0.178
1101-122	SSEVTFPLF	SPMFqN	PTYYTTN	0.334	±	0.014	2.443	±	0.793	2.072	±	0.460
3rd_0.5-0-6G	SSEVTFPFF	SPMFqH	PSYSSTG	0.416	±	0.01	2.429	±	0.474	2.056	±	0.117
1026R71	SSELTFPYF	SPMFqR	PTYLATG	0.321	±	0.006	2.411	±	0.748	2.696	±	0.191
H123#414	SSELMFPMF	SPMFGQ	PRYTSRG	0.194	±	0.004	2.404	±	1.013	4.516	±	0.408
3rd_0.5-2-4E	SSELTFPFF	SPMFqS	PTYAPAP	0.362	±	0.013	2.394	±	0.670	1.875	±	0.277
3rd_0.5-0.125-4B	SSEITFPFF	SPMFqR	PVYWPAG	0.454	±	0.017	2.356	±	0.471	2.255	±	0.227
1101-181	SSEVTFPMF	SPMFTM	PTYLPSY	0.333	±	0.018	2.338	±	1.096	2.750	±	0.472
3rd_0.5-0.25-08A	SSEILFPYF	SPMFqH	PEYKPTH	0.336	±	0.004	2.332	±	0.719	2.386	±	0.128
3rd_0.5-0.125-3G	SSELVFPFF	SPMFGq	PTYRPQA	0.378	±	0.002	2.331	±	0.628	1.967	±	0.111
1011-46	SSETLFPFF	SPMFqF	PAYLVNG	0.320	±	0.004	2.329	±	0.327	2.363	±	0.334
H123#425	SSEVLFPFF	SPMFQR	PTYAPSH	0.215	±	0.008	2.322	±	0.832	3.094	±	0.292
1026B74	SSEVLFPFF	SPMFSL	PSYRPSN	0.302	±	0.020	2.321	±	0.618	3.018	±	0.312
3rd_0.5-0.125-5A	SSELMFPLF	SPMFqL	PTYLAGA	0.375	±	0.013	2.317	±	0.544	2.562	±	0.246
2D-0.5-12B	SSELTFPFF	SPMFLq	PTYFAGF	0.146	±	0.012	2.312	±	0.930	3.067	±	0.362
H123#91	SSELMFPSF	SPMFSN	PTYAPTA	0.375	±	0.071	2.311	±	0.563	2.332	±	0.462
1st-F10-0.1-1A	SSELTFPYF	SPMFKq	PTYVPRG	0.408	±	0.003	2.301	±	0.787	2.495	±	0.226
1026B90	SSELTFPMF	SPMFPA	PTYRAGP	0.297	±	0.026	2.294	±	0.668	3.444	±	0.410
1026B118	SSELLFPYF	SPMFGL	PEYTPRG	0.338	±	0.003	2.269	±	0.725	2.258	±	0.156
3rd_0.5-0.5-3D	SSELLFPLF	SPMFAI	PTYLPTq	0.437	±	0.008	2.258	±	0.438	2.509	±	0.142
3rd_0.5-0-5C	SSETLFPVF	SPMFSY	PAVLCFA	0.366	±	0.006	2.221	±	0.487	1.931	±	0.161
1026B11	SSEILFPYF	SPMFqI	PSYFVSA	0.317	±	0.003	2.202	±	0.349	2.411	±	0.218
3rd_0.5-0.5-2E	SSEVTFSSF	SPMFqF	PTYRPAA	0.463	±	0.040	2.194	±	0.465	1.797	±	0.188
3rd_0.5-0.25-12A	SSELLFPYF	SPMFqH	PAYRVTG	0.540	±	0.018	2.172	±	0.407	2.134	±	0.139
1101-123	SSELMFPYF	SPMFHH	PSYYTTH	0.489	±	0.053	2.153	±	0.682	2.285	±	0.481
1026R82	SSELTFPYF	SPMFqT	PTYYPNG	0.349	±	0.012	2.147	±	0.676	2.371	±	0.175
3rd_1-4-E-06	SSEVTFPFF	SPMFqV	PSYIPSq	0.246	±	0.012	2.141	±	0.612	2.455	±	0.240
1026B64	SSELqFPFF	SPMFKV	PTYLPRH	0.298	±	0.040	2.136	±	0.426	2.804	±	0.485
1101-132	SSELMFPSF	SPMFqI	PTYYASS	0.430	±	0.002	2.135	±	0.689	2.024	±	0.324
1026B9	SSEWNFPYF	SPMFNW	PTYTPSE	0.324	±	0.015	2.134	±	0.386	2.716	±	0.286
1st-F10-0.1-10A	SSEVTFSSF	SPMFPq	PTYRPSA	0.422	±	0.014	2.130	±	0.733	1.366	±	0.094
1101-142	SSELTFPYF	SPMFSL	PTYMPqH	0.304	±	0.004	2.120	±	0.717	2.261	±	0.431
1026B27	SSEITFPYF	SPMFGq	PQYYATF	0.336	±	0.01	2.111	±	0.389	2.636	±	0.224
3rd_1-4-A-03	SSEVMFPYF	SPMFNM	PTYIVGA	0.286	±	0.017	2.104	±	0.553	1.530	±	0.166
3RD-1-3G	SSEIYFPFF	SPMFNq	PTYISNG	0.373	±	0.006	2.079	±	0.489	1.390	±	0.162
1026B117	SSEVMFPAF	SPMFqW	PSYMASG	0.356	±	0.003	2.059	±	0.668	1.227	±	0.091
1011-4	SSEATFPFF	SPMFMq	PTYYPSE	0.406	±	0.004	2.054	±	0.295	2.027	±	0.288
1026R68	SSELLFPVF	SPMFqL	PTYKPSS	0.361	±	0.003	2.046	±	0.627	1.824	±	0.131
3rd_0.5-0.5-3E	SSEVLFPHF	SPMFqL	PTYRPVG	0.541	±	0.046	2.044	±	0.415	1.897	±	0.207
H123#47	SSEVLFPYF	SPMFHT	PSYYPTQ	0.315	±	0.002	2.042	±	0.603	2.924	±	0.233

3rd_0.5-0.0625-11E	SSEFLFPYF	SPMFqT	PSYLPLG	0.542	±	0.034	2.039	±	0.405	2.305	±	0.217
1026R67	SSELqFPFF	SPMFNT	PTYYPTA	0.342	±	0.024	2.022	±	0.712	2.345	±	0.243
1101-8	SSELTFPFF	SPMFAq	PTYRPGH	0.358	±	0.002	2.012	±	0.336	2.329	±	0.296
H123#404	SSELAFPFF	SPMFPN	PQYIVTG	0.180	±	0.004	2.010	±	0.777	3.370	±	0.265
H123#259	SSELLFPYF	SPMFGF	PRYRPSG	0.328	±	0.004	2.009	±	0.888	2.144	±	0.186
1101-96	SSELLFPFF	SPMFGF	PQYYVTE	0.501	±	0.018	2.005	±	0.619	2.335	±	0.393
1101-173	SSEYLFPYF	SPMFYT	PTYYPRD	0.325	±	0.002	2.001	±	0.723	2.115	±	0.470
1026B8	SSELTFPFF	SPMFLV	PSYRPDS	0.316	±	0.003	1.982	±	0.439	2.450	±	0.319
H123#266	SSEITFPFF	SPMFqL	PHYVHTK	0.389	±	0.005	1.980	±	0.804	1.648	±	0.156
3rd_0.5-0.0625-09E	SSELKFPFF	SPMFPq	PTYYPSS	0.403	±	0.005	1.975	±	0.432	2.566	±	0.363
0.25-D1	SSELTFPLF	SPMFPq	PTYLPRG	0.428	±	0.004	1.974	±	0.431	1.874	±	0.256
1026R59	SSEVTFPYF	SPMFqW	PTYVPSH	0.384	±	0.006	1.952	±	0.612	2.301	±	0.164
1101-148	SSELLFPYF	SPMFqK	PYYRVSS	0.416	±	0.003	1.948	±	0.628	1.690	±	0.275
H123#335	SSEYLFPFF	SPMFqI	PRYMTTG	0.255	±	0.009	1.938	±	0.696	2.982	±	0.243
3rd_0.5-1-11H	SSELLFPFF	SPMFqS	PDYYKSP	0.612	±	0.035	1.934	±	0.444	1.735	±	0.261
1101-126	SSELLFPHF	SPMFqH	PNYLPSN	0.425	±	0.009	1.931	±	0.653	1.888	±	0.359
3rd_0.5-1-12A	SSEFSFPYF	SPMFqY	PGYLPSH	0.502	±	0.024	1.924	±	0.443	1.912	±	0.285
3rd_0.5-0.25-07H	SSEYMFPHF	SPMFqL	PRYRPSP	0.375	±	0.017	1.924	±	0.519	1.180	±	0.092
1026B68	SSEVLFPFF	SPMFqQ	PHYRPDH	0.353	±	0.013	1.923	±	0.424	1.987	±	0.176
2nd-0.5-F10-0.1-10D	SSETSFPFF	SPMFqL	PTYLTRG	0.490	±	0.022	1.909	±	0.659	1.580	±	0.160
3rd_0.5-2-3C	SSEVTFSSF	SPMFQq	PTYMNGH	0.339	±	0.010	1.904	±	0.679	1.770	±	0.342
H123#288	SSELTFPFF	SPMFAL	PSYTASP	0.398	±	0.030	1.904	±	0.807	1.704	±	0.192
1101-153	SSELMFPFF	SPMFqL	PSYVPDH	0.287	±	0.008	1.896	±	0.866	2.115	±	0.387
3rd_0.5-0.0625-08G	SSELTFPFF	SPMFYq	PSYYSTY	0.406	±	0.002	1.882	±	0.782	1.998	±	0.313
H123#62	SSELMFPNF	SPMFqH	PQYLPSP	0.622	±	0.039	1.882	±	0.316	2.032	±	0.167
H123#76	SSELLFPYF	SPMFNT	PSYIqSA	0.570	±	0.008	1.879	±	0.259	2.761	±	0.234
3rd_0.5-1-12C	SSEWqFPYF	SPMFTI	PTYLPSS	0.500	±	0.031	1.877	±	0.433	1.696	±	0.255
H123#237	SSEFLFPFF	SPMFqR	PTYAPSS	0.417	±	0.002	1.877	±	0.773	1.856	±	0.161
1026B57	SSELLFPFF	SPMFNq	PTYAPSS	0.314	±	0.012	1.867	±	0.347	2.757	±	0.250
3rd_0.5-0.25-11E	SSELFFPMF	SPMFqH	PRYLPTG	0.394	±	0.011	1.866	±	0.593	1.762	±	0.106
1026B73	SSEIFFPAF	SPMFPq	PSYYPTN	0.343	±	0.037	1.862	±	0.498	2.162	±	0.313
1101-20	SSEVNFPYF	SPMFqL	PEYVPRH	0.345	±	0.011	1.855	±	0.278	1.327	±	0.104
3RD-1-3D	SSEVqFPFF	SPMFAH	PHYLPSS	0.262	±	0.033	1.848	±	0.635	1.426	±	0.218
2ND-1-E3	SSEVTFSSF	SPMFAq	PTYYPDF	0.453	±	0.007	1.835	±	0.368	2.058	±	0.437
1101-43	SSEIMFPYF	SPMFqS	PTYWPTY	0.355	±	0.019	1.830	±	0.310	1.519	±	0.175
1011-6	SSELTFPFF	SPMFqQ	PTYSPSP	0.428	±	0.000	1.822	±	0.298	2.279	±	0.324
3rd_0.5-2-1G	SSEVqFPMF	SPMFMR	PHYIIPY	0.370	±	0.012	1.816	±	0.729	1.423	±	0.221
3rd-1-G1	SSELNFPYF	SPMFqF	PRYYQNG	0.533	±	0.009	1.809	±	0.623	0.918	±	0.076
H123#315	SSEVTFPYF	SPMFqM	PGYRTQG	0.303	±	0.007	1.804	±	0.765	1.550	±	0.135
1011-21	SSEMTFPFF	SPMFqS	PTYLPTS	0.453	±	0.078	1.795	±	0.397	2.054	±	0.464
1101-118	SSEMLFPYF	SPMFqH	PSYLPAE	0.347	±	0.008	1.788	±	0.653	2.071	±	0.315
3rd_0.5-0.125-4C	SSEITFPLF	SPMFqF	PSYMTNA	0.429	±	0.010	1.785	±	0.458	3.446	±	0.474
1101-128	SSEATFPMF	SPMFGq	PTYFPSP	0.338	±	0.005	1.779	±	0.992	1.544	±	0.379
3rd_0.5-4-12D	SSEVqFPYF	SPMFLF	PTYYPGG	0.526	±	0.021	1.777	±	0.375	1.132	±	0.103
3rd_0.5-2-3E	SSEVqFPFF	SPMFqM	PHYIMPL	0.363	±	0.014	1.769	±	0.316	1.098	±	0.208
3rd_0.5-2-3A	SSEYTFPYF	SPMFQq	PTYYPST	0.603	±	0.02	1.768	±	0.327	1.184	±	0.172
1101-36	SSELTFPFF	SPMFqS	PYYFPSQ	0.392	±	0.005	1.767	±	0.279	1.482	±	0.128
3rd_0.5-1-07G	SSEVTFPFF	SPMFqS	PAYTPTH	0.579	±	0.012	1.757	±	0.351	1.068	±	0.072
1026B15	SSEYLFPFF	SPMFqY	PTYRTSA	0.459	±	0.054	1.757	±	0.349	1.491	±	0.213

1026B7	SSELqFPFF	SPMFTS	PTYYPSA	0.448	±	0.008	1.744	±	0.228	1.629	±	0.139
3rd_0.5-1-10B	SSELLFPYF	SPMFqL	PTYNIIP	0.632	±	0.016	1.743	±	0.308	1.560	±	0.240
1101-83	SSEINFPYF	SPMFAq	PTYYPNH	0.392	±	0.011	1.736	±	0.243	1.950	±	0.205
1011-8	SSELTFPFF	SPMFPq	PAYRPTH	0.434	±	0.017	1.734	±	0.323	2.513	±	0.372
1026B59	SSEVLFPYF	SPMFHq	PTYRPTS	0.467	±	0.095	1.732	±	0.426	1.523	±	0.335
1101-95	SSEFqFPSF	SPMFHW	PTYLPRG	0.386	±	0.009	1.731	±	0.308	1.903	±	0.172
1026B31	SSEIqFPYF	SPMFNL	PTYISSG	0.306	±	0.001	1.730	±	0.617	1.989	±	0.160
1101-100	SSEqLFPYF	SPMFNY	PDYLGHD	0.336	±	0.015	1.725	±	0.843	2.299	±	0.359
3rd_0.5-0.0625-12A	SSELqFPFF	SPMFPS	PTYYPNS	0.672	±	0.017	1.725	±	0.366	1.927	±	0.282
1st-F10-0.1-5D	SSELMFPSF	SPMFqI	PTYITRG	0.561	±	0.006	1.715	±	0.589	1.274	±	0.071
1101-12	SSEALFPFF	SPMFqH	PTYTPSA	0.343	±	0.007	1.710	±	0.402	1.656	±	0.200
2D-0.5-7B	SSEVTFPLF	SPMFPq	PSYMPNG	0.176	±	0.013	1.694	±	0.987	3.541	±	0.397
1011-7	SSELLFPMF	SPMFqL	PFYRPTH	0.419	±	0.026	1.691	±	0.387	1.792	±	0.277
3rd_0.5-1-07C	SSEVqFPYF	SPMFHY	PSYMPRD	0.440	±	0.013	1.689	±	0.342	1.377	±	0.127
1101-112	SSEINFPFF	SPMFqS	PTYFVAP	0.315	±	0.026	1.688	±	0.717	2.477	±	0.483
1011-44	SSEMqFPFF	SPMFPR	PTYYTGG	0.361	±	0.009	1.686	±	0.384	1.881	±	0.270
1011-50	SSELNFPYF	SPMFLq	PGYRDDF	0.465	±	0.023	1.684	±	0.219	3.604	±	0.533
1026B5	SSEVPFPFF	SPMFAL	PTYYAGG	0.312	±	0.002	1.683	±	0.426	1.108	±	0.099
1026B85	SSELqFPFF	SPMFNF	PTYRPNq	0.278	±	0.008	1.671	±	0.714	2.641	±	0.264
1101-1	SSEFMFPMF	SPMFqA	PQYVPTP	0.370	±	0.005	1.669	±	0.269	1.966	±	0.216
1101-155	SSEMTFPFF	SPMFGY	PAYLAGP	0.516	±	0.005	1.665	±	0.533	1.766	±	0.319
H123#407	SSESLFPFF	SPMFqY	PTYYPTN	0.373	±	0.028	1.657	±	0.246	2.074	±	0.230
H123#16	SSEFMFPMF	SPMFGR	PqYAQPS	0.505	±	0.012	1.657	±	0.494	2.013	±	0.114
3rd_0.5-0.125-4F	SSEITFPYF	SPMFHq	PTYNTTA	0.691	±	0.015	1.655	±	0.265	1.744	±	0.247
3rd_0.5-0.25-07D	SSEWYFPFF	SPMFQM	PHYLPSN	0.365	±	0.001	1.650	±	0.559	1.732	±	0.092
1011-42	SSELMFPSF	SPMFHR	PHYLRPA	0.386	±	0.006	1.644	±	0.285	1.759	±	0.273
1026B128	SSEWSFPYF	SPMFqF	PDYITHG	0.419	±	0.011	1.644	±	0.507	1.431	±	0.103
1026B38	SSEITFPHF	SPMFqR	PSYVPNA	0.311	±	0.002	1.642	±	0.618	2.320	±	0.198
1101-85	SSEIGFPFF	SPMFqH	PEYINSH	0.380	±	0.001	1.642	±	0.290	1.765	±	0.173
1011-403	SSEVTFPMF	SPMFqS	PDYMRSP	0.369	±	0.001	1.630	±	0.510	1.379	±	0.136
3rd_0.5-4-09B	SSELqFPHF	SPMFSY	PTYLPRS	0.612	±	0.023	1.628	±	0.314	1.593	±	0.224
1026B23	SSEFMFPYF	SPMFAN	PTYRPTP	0.514	±	0.028	1.623	±	0.240	1.748	±	0.188
1026B89	SSEVMFPHF	SPMFQY	PSYVPGH	0.390	±	0.037	1.621	±	0.390	2.205	±	0.278
3rd-0.25-9F	SSEVSFPSF	SPMFqT	PTYLPSP	0.535	±	0.001	1.619	±	0.557	0.803	±	0.059
H123#204	SSEITFPMF	SPMFHL	PVYITNG	0.324	±	0.003	1.617	±	0.374	2.733	±	0.327
1101-9	SSEVLFPHF	SPMFNq	PQYMPTG	0.329	±	0.010	1.610	±	0.410	1.828	±	0.173
1101-27	SSEVSFPFF	SPMFqH	PTYMPHD	0.413	±	0.016	1.607	±	0.265	1.281	±	0.106
3rd_0.5-0.5-1H	SSEFqFPFF	SPMFAL	PDYLPTH	0.355	±	0.005	1.596	±	0.673	2.454	±	0.151
1101-14	SSELMFPYF	SPMFqM	PTYNVRA	0.415	±	0.014	1.592	±	0.246	1.764	±	0.159
1026R85	SSELLFPYF	SPMFqR	PGYRPAG	0.477	±	0.015	1.591	±	0.533	1.513	±	0.260
1st-F10-0.1-12F	SSEVTFSSF	SPMFqT	PTYNPSY	0.487	±	0.016	1.590	±	0.584	1.220	±	0.239
1026B6	SSELTFPFF	SPMFSI	PRYFPSA	0.520	±	0.057	1.588	±	0.284	1.530	±	0.213
1011-29	SSELqFPFF	SPMFPW	PSYRPSP	0.433	±	0.014	1.584	±	0.239	1.668	±	0.258
1101-63	SSEFTFPFF	SPMFqL	PSYLPTN	0.441	±	0.033	1.580	±	0.241	1.813	±	0.301
1101-4	SSEVTFSSF	SPMFGH	PMYYPVP	0.407	±	0.025	1.579	±	0.270	1.842	±	0.210
1026R78	SSEMTFPFF	SPMFAq	PSYITRG	0.451	±	0.005	1.578	±	0.511	1.753	±	0.137
1026B58	SSEVTFPFF	SPMFqN	PTYNKPV	0.474	±	0.009	1.570	±	0.279	1.398	±	0.117
H123#80	SSEWAFPHF	SPMFqF	PTYHPGH	0.505	±	0.034	1.570	±	0.315	1.785	±	0.221
1101-185	SSEVqFPFF	SPMFQL	PTYFIRG	0.309	±	0.028	1.567	±	1.194	0.714	±	0.208

H123#212	SSELTFPMF	SPMFPq	PEYKPSH	0.346	±	0.001	1.556	±	0.212	2.635	±	0.254
H123#305	SSEVTFPFF	SPMFPS	PTYRTMG	0.492	±	0.030	1.556	±	0.640	2.000	±	0.217
1101-22	SSELTFPYF	SPMFAq	PFYYVTQ	0.374	±	0.016	1.547	±	0.498	1.796	±	0.155
1026B86	SSEMTFPYF	SPMFqH	PGYLPEP	0.500	±	0.071	1.544	±	0.311	1.777	±	0.303
3rd_0.5-0-5A	SSEIGFPYF	SPMFNL	PSYIANA	0.597	±	0.025	1.540	±	0.304	1.345	±	0.204
1101-140	SSEFqFPFF	SPMFNH	PTYLTHG	0.325	±	0.004	1.534	±	0.623	1.833	±	0.284
1101-190	SSEITFPMF	SPMFqL	PAYRPSL	0.415	±	0.018	1.524	±	0.741	1.576	±	0.263
1101-117	SSELLFPMF	SPMFqV	PTYAPSL	0.436	±	0.023	1.522	±	0.619	1.569	±	0.258
1026B61	SSEVAFPFF	SPMFPq	PTYYSTA	0.501	±	0.008	1.521	±	0.215	1.400	±	0.118
H123#135	SSEVLFPFF	SPMFGP	PTYYPTS	0.380	±	0.013	1.517	±	0.286	2.085	±	0.214
1026B40	SSELAFPHF	SPMFqQ	PTYLPRA	0.489	±	0.014	1.517	±	0.207	1.408	±	0.153
1026R62	SSELVFPFF	SPMFTT	PTYYVNS	0.460	±	0.003	1.513	±	0.526	1.799	±	0.119
3RD-1-2D	SSEVMFPAF	SPMFqT	PSYHPSq	0.546	±	0.017	1.510	±	0.286	0.707	±	0.105
3rd_0.5-0-4E	SSELFFPFF	SPMFQL	PAYKTNA	0.589	±	0.019	1.503	±	0.302	1.405	±	0.202
1st-F10-0.1-7C	SSEVTFPLF	SPMFqR	PQYHVSG	0.393	±	0.003	1.500	±	0.547	0.922	±	0.052
1101-46	SSEMTFPYF	SPMFYq	PRYMNDF	0.400	±	0.008	1.498	±	0.227	1.441	±	0.135
H123#176	SSEVLFPMF	SPMFqQ	PTYISKG	0.358	±	0.014	1.497	±	0.271	2.059	±	0.223
H123#207	SSEVqFPFF	SPMFQI	PEYLRGT	0.348	±	0.006	1.495	±	0.311	2.119	±	0.224
3rd_0.5-1-09C	SSELTFPYF	SPMFDI	PTYKSKG	0.687	±	0.013	1.491	±	0.231	1.519	±	0.223
1101-5	SSELLFPYF	SPMFAq	PNYHPSN	0.376	±	0.006	1.488	±	0.265	1.422	±	0.117
1026B33	SSEYLFPFF	SPMFGq	PTYWPPT	0.307	±	0.003	1.480	±	0.613	1.987	±	0.160
H123#465	SSELTFPFF	SPMFAQ	PAYVPSF	0.438	±	0.006	1.479	±	0.206	1.892	±	0.274
1101-60	SSEVTFPYF	SPMFQT	PSYLHPK	0.417	±	0.012	1.479	±	0.251	1.340	±	0.173
2nd-0.5-F10-0.1-11A	SSELTFPLF	SPMFqF	PTYFPSE	0.614	±	0.025	1.475	±	0.510	1.045	±	0.123
1101-50	SSEFMFPMF	SPMFNq	PTYYPSK	0.480	±	0.011	1.475	±	0.197	1.291	±	0.170
H123#419	SSELqFPFF	SPMFGW	PTYKAWG	0.324	±	0.049	1.472	±	0.450	1.841	±	0.324
2ND-1-H3	SSELLFPFF	SPMFqY	PRYLISP	0.427	±	0.020	1.471	±	0.607	1.319	±	0.178
1026R86	SSELLFPFF	SPMFNq	PSYRPSF	0.515	±	0.015	1.469	±	0.470	1.461	±	0.104
1026R57	SSENLFPYF	SPMFTV	PTYFPNH	0.369	±	0.005	1.469	±	0.562	1.768	±	0.134
1011-36	SSEMLFPFF	SPMFGF	PSYNTSG	0.389	±	0.030	1.466	±	0.254	2.247	±	0.406
H123#160	SSELHFPYF	SPMFqY	PSYQTRG	0.398	±	0.005	1.463	±	0.308	2.321	±	0.228
H123#352	SSELqFPFF	SPMFPR	PRYYPSG	0.251	±	0.010	1.461	±	0.868	2.612	±	0.247
H123#252	SSEITFPSF	SPMFSH	PSYIPSq	0.443	±	0.006	1.461	±	0.697	1.492	±	0.129
1101-82	SSELIFPFF	SPMFqT	PTYTPNA	0.386	±	0.010	1.456	±	0.270	1.469	±	0.156
1101-75	SSELTFPFF	SPMFVq	PTYMPSS	0.412	±	0.001	1.451	±	0.246	1.597	±	0.194
1011-17	SSEFMFPYF	SPMFPq	PTYLPTP	0.468	±	0.008	1.448	±	0.190	1.531	±	0.221
3rd_0.5-0-6C	SSELqFPFF	SPMFAS	PQYYPTN	0.709	±	0.030	1.442	±	0.293	1.464	±	0.216
H123#38	SSELQFPYF	SPMFAL	PTYYVSq	0.518	±	0.006	1.440	±	0.443	2.280	±	0.172
1026B30	SSEVTFPFF	SPMFPq	PRYGVTP	0.491	±	0.016	1.437	±	0.217	1.010	±	0.094
1101-144	SSEFqFPVF	SPMFGY	PTYYVSQ	0.334	±	0.032	1.428	±	0.742	1.076	±	0.270
1101-51	SSEVTFPHF	SPMFSH	PTYTPSP	0.494	±	0.026	1.426	±	0.205	1.304	±	0.149
1026B108	SSEVVFPLF	SPMFqQ	PTYLPSN	0.546	±	0.022	1.411	±	0.436	1.108	±	0.097
1101-37	SSEFLFPFF	SPMFHS	PSYLPGG	0.501	±	0.016	1.411	±	0.212	1.788	±	0.163
H123#308	SSEWMFPYF	SPMFNA	PTYLAQG	0.513	±	0.006	1.408	±	0.590	2.310	±	0.196
1026R70	SSEWqFPYF	SPMFDI	PTYLASE	0.462	±	0.011	1.402	±	0.533	1.291	±	0.092
1026B76	SSEVAFPFF	SPMFSL	PQYLPVP	0.433	±	0.005	1.395	±	0.291	1.274	±	0.108
3rd_0.5-0.25-11C	SSEMTFPFF	SPMFHq	PSYMPSP	0.774	±	0.030	1.389	±	0.267	1.132	±	0.081
1101-81	SSELTFPYF	SPMFPS	PTYLPRA	0.416	±	0.006	1.389	±	0.400	1.363	±	0.156
H123#13	SSEINFPFF	SPMFqI	PTYSSYN	0.574	±	0.010	1.386	±	0.277	2.293	±	0.145

1101-79	SSEIAFPFF	SPMFqL	PTYLPQD	0.491	±	0.072	1.385	±	0.278	1.311	±	0.236
1101-116	SSELTFPMF	SPMFGq	PRYTVRG	0.298	±	0.003	1.385	±	0.515	1.793	±	0.312
3rd_0.5-2-4A	SSEMqFPSF	SPMFQH	PTYYPTE	0.582	±	0.010	1.385	±	0.241	1.517	±	0.211
1026B44	SSEFLFPMF	SPMFPH	PQYTPSP	0.315	±	0.008	1.384	±	0.541	2.195	±	0.187
3rd_0.5-0.125-3B	SSELqFPFF	SPMFPN	PSYWPTE	0.429	±	0.005	1.384	±	0.586	2.027	±	0.308
1011-9	SSELNFPHF	SPMFqH	PHYIPSG	0.415	±	0.011	1.376	±	0.412	2.339	±	0.366
1026B110	SSEFMFPMF	SPMFqW	PAYWPPS	0.513	±	0.010	1.372	±	0.448	1.125	±	0.080
3rd_1-0-C-12	SSELTFPMF	SPMFqN	PTYHATH	0.655	±	0.011	1.372	±	0.224	1.446	±	0.258
3rd_0.5-0.5-6A	SSELLFPFF	SPMFSH	PYYLPSE	0.830	±	0.057	1.371	±	0.275	1.378	±	0.124
H123#83	SSEWQFPFF	SPMFQN	PTYSPSS	0.835	±	0.040	1.369	±	0.227	1.983	±	0.149
1101-133	SSEVSFPFF	SPMFPq	PYYYATG	0.288	±	0.001	1.368	±	0.782	2.080	±	0.436
1026B28	SSELLFPYF	SPMFqK	PTYFTTN	0.618	±	0.043	1.360	±	0.209	1.454	±	0.159
H123#12	SSEWSFPYF	SPMFYQ	PSYLPSL	0.853	±	0.034	1.356	±	0.152	1.680	±	0.111
H123#224	SSEFLFPYF	SPMFqY	PSYSVTq	0.486	±	0.011	1.352	±	0.558	1.362	±	0.121
1101-134	SSEFqFPYF	SPMFQF	PSYYATY	0.327	±	0.001	1.352	±	0.450	1.056	±	0.247
1st-F10-0.1-2H	SSELLFPFF	SPMFDS	PTYRTSP	0.588	±	0.029	1.341	±	0.498	1.556	±	0.118
H123#159	SSELTFPYF	SPMFqL	PRYYPSH	0.433	±	0.010	1.339	±	0.387	2.148	±	0.212
1026B124	SSELMFPMF	SPMFSq	PTYYPSN	0.591	±	0.057	1.337	±	0.429	1.186	±	0.140
1101-192	SSEFqFPAF	SPMFQL	PSYLPAP	0.389	±	0.030	1.332	±	1.112	1.418	±	0.270
H123#107	SSESAFPYF	SPMFqF	PTYITGS	0.579	±	0.069	1.327	±	0.241	1.377	±	0.255
3rd-1-B2	SSELFFPFF	SPMFqS	PTYLPSE	0.621	±	0.011	1.324	±	0.453	0.890	±	0.053
1026B4	SSEITFPFF	SPMFSq	PAYRATA	0.366	±	0.015	1.318	±	0.249	1.589	±	0.167
3rd_1-0.0625-D-4	SSETLFPFF	SPMFqL	PTYVPGP	0.750	±	0.009	1.317	±	0.211	1.451	±	0.191
1101-7	SSEIqFPFF	SPMFSA	PEYIRGT	0.347	±	0.008	1.313	±	0.396	1.645	±	0.209
3rd_0.5-0.25-12C	SSELTFPNF	SPMFPS	PGYHVSH	0.670	±	0.024	1.307	±	0.272	1.241	±	0.081
3rd_1-0.0625-B-4	SSEVPFPYF	SPMFqF	PTYLPGG	0.716	±	0.004	1.301	±	0.210	0.870	±	0.094
1026B69	SSEFNFPYF	SPMFqF	PAYIPRG	0.627	±	0.008	1.301	±	0.204	1.424	±	0.117
1101-182	SSEWqFPYF	SPMFQH	PEYAPSE	0.317	±	0.003	1.299	±	0.500	1.691	±	0.334
1026B94	SSEVVFPSF	SPMFGq	PTYQPTP	0.480	±	0.019	1.298	±	0.296	1.173	±	0.126
1026B67	SSEFLFPMF	SPMFqR	PQYVPSF	0.369	±	0.026	1.298	±	0.372	1.692	±	0.213
1026B3	SSEVqFPFF	SPMFPT	PTYLPTA	0.639	±	0.044	1.297	±	0.199	1.120	±	0.118
3rd_0.5-4-12F	SSELTFPLF	SPMFQq	PTYYPIN	0.599	±	0.009	1.291	±	0.320	1.767	±	0.247
3rd_0.5-0.0625-12G	SSELSFPFF	SPMFYK	PLYITRG	0.636	±	0.005	1.278	±	0.309	1.528	±	0.218
3rd_0.5-4-07A	SSEFqFPYF	SPMFHA	PQYTRPS	0.458	±	0.056	1.275	±	0.543	1.238	±	0.232
3rd_0.5-0.125-1G	SSELTFPFF	SPMFQR	PTYqVGM	0.380	±	0.007	1.274	±	0.401	2.133	±	0.329
1101-156	SSEVLFPFF	SPMFqT	PGYMVQS	0.425	±	0.006	1.274	±	0.650	0.749	±	0.129
1101-180	SSELTFPYF	SPMFNQ	PqYLTTA	0.335	±	0.006	1.273	±	0.536	1.807	±	0.346
3RD-1-2G	SSERMFPSF	SPMFqF	PSYLPNN	0.665	±	0.005	1.270	±	0.219	0.808	±	0.100
1101-84	SSEIqFPFF	SPMFPH	PSYYASH	0.561	±	0.004	1.267	±	0.162	1.219	±	0.115
1011-405	SSEMLFPFF	SPMFGF	PSYNTSG	0.319	±	0.005	1.264	±	0.716	2.399	±	0.175
1011-47	SSELMFPFF	SPMFQK	PTYLARA	0.617	±	0.011	1.263	±	0.203	1.553	±	0.220
1101-187	SSEVTFSSF	SPMFqW	PAYRPNS	0.284	±	0.012	1.258	±	1.658	0.921	±	0.193
1st-F10-0.1-2C	SSEFqFPHF	SPMFQT	PTYLPSF	0.619	±	0.018	1.254	±	0.473	0.941	±	0.096
1026R53	SSELLFPHF	SPMFqI	PHYLPTN	0.630	±	0.024	1.252	±	0.386	1.464	±	0.131
H123#98	SSEWLFPFF	SPMFNq	PTYQPGH	0.435	±	0.042	1.251	±	0.342	1.777	±	0.349
H123#121	SSEILFPMF	SPMFqN	PTYYVDH	0.446	±	0.005	1.251	±	0.176	1.756	±	0.193
1026R96	SSEVTFSSF	SPMFQY	PTYRPGG	0.532	±	0.008	1.250	±	0.427	1.510	±	0.123
1101-177	SSELqFPYF	SPMFPY	PTYYPNN	0.534	±	0.117	1.246	±	0.564	1.296	±	0.361
H123#36	SSELqFPMF	SPMFGY	PRYQTAG	0.331	±	0.008	1.245	±	0.655	1.719	±	0.160

2nd-11D	SSELMFPSF	SPMFGQ	PHYLPKG	0.711	±	0.041	1.245	±	0.434	1.025	±	0.136
3rd_0.5-0.125-4E	SSELTFPYF	SPMFHq	PVYHVSH	0.816	±	0.022	1.243	±	0.200	1.367	±	0.206
1101-39	SSEVTFPFF	SPMFqN	PFYNRYS	0.520	±	0.001	1.243	±	0.175	1.044	±	0.105
H123#205	SSELLFPMF	SPMFGL	PHYLPSq	0.528	±	0.010	1.239	±	0.191	2.155	±	0.215
3rd_0.5-0.5-4H	SSELLFPFF	SPMFSI	PYYRPTN	0.349	±	0.009	1.232	±	0.607	2.004	±	0.169
3rd_0.5-4-12C	SSELTFPYF	SPMFNQ	PTYFTSq	0.934	±	0.082	1.230	±	0.251	1.131	±	0.119
3rd_0.5-4-10C	SSEVTFPFF	SPMFNq	PDYLKSP	0.893	±	0.017	1.228	±	0.211	1.002	±	0.146
H123#15	SSEVMFPAF	SPMFTT	PTYKPNG	0.732	±	0.007	1.227	±	0.281	1.594	±	0.085
1026R95	SSELMFPHF	SPMFqS	PSYIPRG	0.625	±	0.012	1.226	±	0.452	1.383	±	0.108
2D-0.5-9G	SSELTFPHF	SPMFqT	PAYLPSE	0.359	±	0.011	1.224	±	0.527	1.413	±	0.132
1101-163	SSEFLFPMF	SPMFqH	PGYTTTG	0.296	±	0.005	1.220	±	0.793	1.715	±	0.276
H123#188	SSELLFPFF	SPMFGq	PSYVPAH	0.468	±	0.022	1.216	±	0.223	1.935	±	0.208
1101-49	SSELMFPYF	SPMFqV	PSYFPGQ	0.558	±	0.005	1.215	±	0.158	1.020	±	0.137
3rd_0.5-4-11D	SSEVTFSSF	SPMFHQ	PSYMNPK	0.355	±	0.001	1.210	±	0.953	0.908	±	0.146
1026B43	SSEWqFPYF	SPMFHA	PRYRTEG	0.326	±	0.011	1.209	±	0.626	2.130	±	0.188
3RD-1-3H	SSEqMFPYF	SPMFHI	PGYLPRG	0.502	±	0.006	1.208	±	0.668	0.884	±	0.096
1101-54	SSELTFPYF	SPMFTY	PTYHPTP	0.585	±	0.011	1.207	±	0.187	1.208	±	0.178
1101-13	SSEILFPYF	SPMFqV	PYYMPQS	0.472	±	0.015	1.199	±	0.324	1.258	±	0.116
2ND-1-H4	SSELTFPFF	SPMFTq	PMYYVVP	0.599	±	0.027	1.194	±	0.518	0.958	±	0.112
1101-26	SSEMQFPYF	SPMFqF	PSYLPSS	0.397	±	0.011	1.178	±	0.604	1.239	±	0.128
3rd_0.5-1-08F	SSEFLFPYF	SPMFqA	PLYATRG	0.913	±	0.138	1.177	±	0.303	1.036	±	0.170
1st-F10-0.1-8A	SSELLFPSF	SPMFAK	PTYLPRG	0.615	±	0.027	1.176	±	0.438	1.098	±	0.200
1101-146	SSEYLFPYF	SPMFGR	PTYLPRE	0.883	±	0.023	1.172	±	0.353	1.284	±	0.210
H123#341	SSELqFPFF	SPMFDF	PSYVPSH	0.453	±	0.086	1.166	±	0.266	1.223	±	0.250
3rd_0.5-2-4C	SSELqFPYF	SPMFSL	PAYLASP	0.468	±	0.006	1.164	±	0.563	1.104	±	0.156
1101-23	SSEITFPFF	SPMFqT	PHYTPSA	0.573	±	0.086	1.164	±	0.246	1.463	±	0.267
1101-91	SSEFTFPSF	SPMFNK	PSYLIPT	0.537	±	0.009	1.162	±	0.152	0.933	±	0.096
H123#250	SSEMLFPYF	SPMFSS	PGYRPEA	0.639	±	0.029	1.157	±	0.479	1.188	±	0.116
1026B18	SSELLFPFF	SPMFqQ	PTYQRPA	0.332	±	0.004	1.156	±	0.445	1.906	±	0.153
1011-20	SSELTFPFF	SPMFSq	PGYFASH	0.364	±	0.014	1.155	±	0.219	1.595	±	0.246
3rd-0.25-11C	SSELLFPYF	SPMFGL	PQYYPSq	0.804	±	0.032	1.146	±	0.395	0.781	±	0.070
2ND-1-A2	SSELVFPFF	SPMFqY	PSYRAAG	0.367	±	0.002	1.146	±	0.530	1.529	±	0.185
H123#3	SSEGTFPFF	SPMFGH	PTYYVSH	0.626	±	0.007	1.143	±	0.259	1.572	±	0.183
H123#276	SSEVVFPSF	SPMFLT	PTYLPND	0.648	±	0.070	1.132	±	0.477	0.907	±	0.129
H123#10	SSEWTFPLF	SPMFPY	PTYYLNP	0.565	±	0.031	1.123	±	0.460	1.052	±	0.081
H123#427	SSELAFPAF	SPMFPR	PSYQPSP	0.418	±	0.020	1.123	±	0.381	1.395	±	0.144
2nd-0.5-F10-0.1-11E	SSEWVFPLF	SPMFNH	PTYVPRG	0.566	±	0.008	1.123	±	0.400	1.417	±	0.148
H123#44	SSELTFPAF	SPMFHH	PqYWqSP	0.355	±	0.003	1.123	±	0.685	0.765	±	0.082
H123#69	SSEMLFPLF	SPMFqW	PTYSPSS	0.369	±	0.006	1.121	±	0.576	1.934	±	0.113
1011-26	SSELMFPMF	SPMFqN	PAYRPTL	0.657	±	0.091	1.115	±	0.207	0.938	±	0.190
1011-23	SSELLFPLF	SPMFqS	PTYRPSE	0.325	±	0.004	1.111	±	0.580	2.844	±	0.410
H123#179	SSEITFPYF	SPMFRq	PTYLPTP	0.608	±	0.023	1.111	±	0.170	1.487	±	0.161
3rd_0.5-0-2F	SSELqFPFF	SPMFLT	PQYIPSG	0.389	±	0.006	1.109	±	0.831	1.598	±	0.244
H123#58	SSEVTFPSF	SPMFqR	PTYIRTK	0.359	±	0.004	1.109	±	0.419	0.565	±	0.046
1101-80	SSEYMFPYF	SPMFHS	PTYRPSL	0.643	±	0.050	1.108	±	0.161	0.945	±	0.160
3rd_0.5-0-6D	SSELLFPFF	SPMFGR	PTYRPQA	1.020	±	0.008	1.106	±	0.211	1.110	±	0.059
H123#294	SSELMFPYF	SPMFHQ	PTYSPTD	0.495	±	0.062	1.106	±	0.622	1.217	±	0.183
1011-34	SSELTFPFF	SPMFAq	PSYKVSH	0.386	±	0.022	1.105	±	0.233	1.534	±	0.247
H123#128	SSEFTFPFF	SPMFNT	PSYQPSS	0.602	±	0.027	1.104	±	0.177	1.682	±	0.180

H123#115	SSERqFPIF	SPMFGL	PVYRPTH	0.546	±	0.062	1.102	±	0.212	1.221	±	0.232
H123#246	SSELTFPMF	SPMFqQ	PKYLTTG	0.476	±	0.001	1.101	±	0.502	1.078	±	0.096
1st-F10-0.1-8F	SSELLFPMF	SPMFNL	PTYMSHG	0.683	±	0.114	1.100	±	0.425	1.711	±	0.302
H123#469	SSEFLFPMF	SPMFNT	PTYTPNA	0.763	±	0.043	1.099	±	0.154	1.385	±	0.209
3rd_1-0-D-8	SSEITFPFF	SPMFqF	PGYLRAH	0.775	±	0.016	1.096	±	0.200	1.060	±	0.126
1101-151	SSELFFPYF	SPMFSq	PTYNTTP	0.385	±	0.009	1.095	±	0.500	1.376	±	0.335
1101-121	SSELNFPFF	SPMFAL	PSYKPNY	0.440	±	0.032	1.095	±	0.839	1.515	±	0.350
1st-F10-0.1-5C	SSEVTFPFF	SPMFqS	PTYLVGH	0.762	±	0.022	1.093	±	0.384	0.837	±	0.110
H123#123	SSEVTFPLF	SPMFqN	PHYNPRH	0.502	±	0.013	1.088	±	0.156	0.795	±	0.090
H123#475	SSEFqFPFF	SPMFMH	PSYLPTY	0.547	±	0.005	1.087	±	0.350	1.300	±	0.199
1026B95	SSEVTFPFF	SPMFGq	PTYSSGK	0.382	±	0.022	1.084	±	0.341	1.507	±	0.148
H123#202	SSEFTFPNF	SPMFqY	PTYVPSP	0.452	±	0.034	1.081	±	0.186	1.651	±	0.214
H123#245	SSEFLFPYF	SPMFqA	PSYYPNS	0.769	±	0.065	1.080	±	0.449	0.998	±	0.128
H123#172	SSELTFPLF	SPMFLY	PLYLPSP	0.396	±	0.005	1.077	±	0.340	1.197	±	0.141
3rd_1-0-A-10	SSEFSFPMF	SPMFqL	PTYLPTN	0.877	±	0.019	1.073	±	0.185	1.284	±	0.172
1101-147	SSEFSFPYF	SPMFqH	PTYVPNL	0.406	±	0.012	1.068	±	0.486	0.998	±	0.229
1011-27	SSELMFPYF	SPMFPq	PTYRPSS	0.565	±	0.025	1.068	±	0.228	1.154	±	0.175
H123#28	SSELTFPNF	SPMFqQ	PGYVPEA	0.425	±	0.016	1.064	±	0.771	0.765	±	0.055
1026R80	SSEIVFPYF	SPMFHN	PSYRPNH	0.564	±	0.014	1.063	±	0.385	1.301	±	0.095
3RD-1-1D	SSEVTFPFF	SPMFAq	PSYVTSP	0.860	±	0.044	1.063	±	0.202	0.588	±	0.075
H123#86	SSEVMFPAF	SPMFGV	PQYYTDH	0.901	±	0.026	1.058	±	0.163	0.899	±	0.063
3rd_0.5-1-09G	SSEGqFPFF	SPMFTW	PTYYVAP	0.633	±	0.036	1.058	±	0.390	0.658	±	0.117
3rd_0.5-2-6C	SSELLFPYF	SPMFHL	PTYLPTN	0.982	±	0.028	1.057	±	0.249	0.943	±	0.130
H123#120	SSEITFPYF	SPMFAH	PTYVPKD	0.685	±	0.030	1.057	±	0.162	1.358	±	0.206
H123#148	SSEWTFPMF	SPMFQR	PTYFHGK	0.561	±	0.009	1.056	±	0.165	1.715	±	0.215
H123#381	SSEVNFPYF	SPMFqT	PGYRTGG	0.291	±	0.026	1.054	±	0.686	1.077	±	0.126
H123#63	SSEFLFPFF	SPMFNV	PHYFSKA	0.287	±	0.002	1.053	±	1.413	0.747	±	0.072
1026R61	SSELTFPYF	SPMFNq	PTYAKPM	0.606	±	0.003	1.050	±	0.334	1.061	±	0.073
H123#203	SSELMFPSF	SPMFqL	PTYSADS	0.491	±	0.045	1.040	±	0.228	1.234	±	0.171
1011-56	SSELLFPYF	SPMFqF	PGYTPSQ	0.699	±	0.034	1.037	±	0.136	1.254	±	0.189
3rd_0.5-0.0625-10H	SSEVLFPLF	SPMFqR	PSYTPRG	0.425	±	0.010	1.034	±	0.678	1.856	±	0.268
3RD-1-5A	SSEVMFPVF	SPMFHK	PTYLPSF	0.813	±	0.035	1.032	±	0.188	0.858	±	0.109
3rd_0.5-0-6E	SSELLFPFF	SPMFqN	PDYRAWG	0.499	±	0.004	1.030	±	0.464	1.480	±	0.108
3rd_0.5-0.125-6F	SSELLFPFF	SPMFqN	PTYAPID	1.042	±	0.032	1.029	±	0.173	1.010	±	0.149
3rd_1-0-E-10	SSEVTFSSF	SPMFqT	PKYMPSS	0.787	±	0.031	1.026	±	0.186	0.520	±	0.094
H123#468	SSELTFPFF	SPMFHT	PSYRPSA	0.841	±	0.016	1.020	±	0.139	1.468	±	0.209
3rd_0.5-0.25-10A	SSEAAFPFF	SPMFqL	PTYMQPK	1.105	±	0.028	1.019	±	0.198	0.568	±	0.048
1026B26	SSEVTFPYF	SPMFSL	PTYKSGF	0.581	±	0.079	1.009	±	0.533	1.030	±	0.163
2D-0.5-11D	SSEFLFPFF	SPMFPq	PTYQPRH	0.908	±	0.042	1.004	±	0.182	0.735	±	0.086
3rd_1-4-F-02	SSELMFPFF	SPMFqH	PFYHPTN	0.486	±	0.017	1.001	±	0.318	1.126	±	0.108
H123#417	SSELAFPFF	SPMFqT	PSYNISK	0.333	±	0.005	0.998	±	0.721	2.391	±	0.175
2nd-0.5-F10-0.1-7E	SSELYFPVF	SPMFAQ	PAYVPSF	0.840	±	0.051	0.996	±	0.348	0.754	±	0.099
H123#284	SSEVTFPSF	SPMFqL	PYYLTSS	0.552	±	0.003	0.995	±	0.426	0.763	±	0.073
3rd-0.5-H10	SSELqFPYF	SPMFNH	PTYHVSA	0.656	±	0.016	0.990	±	0.349	0.682	±	0.043
3rd_0.5-1-11A	SSELTFPHF	SPMFGI	PTYFGTq	1.060	±	0.119	0.985	±	0.201	0.981	±	0.172
1101-53	SSELqFPFF	SPMFPT	PSYIPRH	0.666	±	0.149	0.984	±	0.261	0.780	±	0.186
H123#124	SSEFMFPSF	SPMFGq	PTYRVDG	0.415	±	0.009	0.984	±	0.342	1.541	±	0.157
1011-18	SSEFqFPFF	SPMFQV	PAYLPTE	0.481	±	0.015	0.984	±	0.338	1.339	±	0.198
1011-2	SSELTFPFF	SPMFSS	PHYTTSG	0.487	±	0.036	0.983	±	0.262	1.648	±	0.264

1st-F10-0.1-4B	SSELTFPLF	SPMFNq	PTYLVSH	0.492	±	0.005	0.982	±	0.356	0.817	±	0.046
H123#127	SSEYLFPYF	SPMFSQ	PVYYTDG	0.616	±	0.011	0.982	±	0.147	1.535	±	0.155
1011-5	SSEMTFPFF	SPMFSN	PAYRPSM	0.663	±	0.007	0.981	±	0.189	1.119	±	0.160
1st-F10-0.1-10G	SSEIVFPHF	SPMFNq	PAYTPRG	0.730	±	0.041	0.979	±	0.371	0.502	±	0.068
H123#133	SSEHVFPYF	SPMFSH	PTYYASF	0.390	±	0.014	0.976	±	0.285	1.255	±	0.131
H123#14	SSETIFPFF	SPMFAL	PTYMPSP	0.484	±	0.017	0.976	±	0.538	1.831	±	0.121
H123#66	SSELTFPLF	SPMFAS	PTYLEPT	0.531	±	0.031	0.975	±	0.418	0.782	±	0.096
H123#311	SSELqFPYF	SPMFQA	PSYTPRG	0.349	±	0.006	0.970	±	0.496	1.559	±	0.135
H123#324	SSEVNFPLF	SPMFPN	PTYYVTq	0.126	±	0.004	0.968	±	3.551	2.601	±	0.214
1st-F10-0.1-5F	SSEVLFPYF	SPMFqL	PSYRPSS	0.905	±	0.041	0.968	±	0.335	0.646	±	0.045
1026B105	SSEIMFPYF	SPMFFq	PSYLPSQ	0.685	±	0.055	0.967	±	0.321	0.883	±	0.095
2ND-0.5-F10-0-E5	SSEFLFPYF	SPMFqL	PRYLTPA	0.706	±	0.031	0.964	±	0.347	1.052	±	0.070
3rd_0.5-0.25-08E	SSELTFPLF	SPMFHI	PTYVRPS	0.479	±	0.003	0.962	±	0.893	1.601	±	0.090
2ND-1-A5	SSEWTFPYF	SPMFqY	PQYFRPS	0.176	±	0.006	0.953	±	0.764	1.724	±	0.177
H123#273	SSELHFPHF	SPMFMH	PTYLNSP	0.268	±	0.002	0.952	±	0.950	0.756	±	0.078
1011-22	SSELLFPFF	SPMFNR	PHYRPIE	0.449	±	0.029	0.951	±	0.395	1.748	±	0.277
3rd_0.5-0.5-1A	SSELTFPYF	SPMFqN	PTYFRSG	0.339	±	0.002	0.949	±	0.992	1.861	±	0.175
1101-160	SSELAFPFF	SPMFMV	PQYRPSG	0.466	±	0.046	0.944	±	0.692	1.712	±	0.314
1011-39	SSEMMFPSF	SPMFqA	PEYRPSS	0.871	±	0.071	0.942	±	0.157	0.944	±	0.155
1101-92	SSEIPFPFF	SPMFqR	PTYLPSL	0.590	±	0.020	0.939	±	0.213	0.721	±	0.080
3RD-0.25-7F	SSEMSFPFF	SPMFPq	PTYMPSL	0.337	±	0.009	0.936	±	0.507	1.486	±	0.131
3rd-0.25-11E	SSELVFPMF	SPMFqT	PTYQPNS	0.849	±	0.067	0.934	±	0.355	0.637	±	0.060
3rd_0.5-0-2C	SSELqFPFF	SPMFPL	PGYNPSA	0.428	±	0.003	0.931	±	0.792	1.580	±	0.226
1026B13	SSEWRFPYF	SPMFLq	PTYYPDS	0.512	±	0.008	0.930	±	0.320	1.074	±	0.089
1101-72	SSEFLFPHF	SPMFGq	PTYQPTQ	0.685	±	0.016	0.930	±	0.160	0.918	±	0.136
1101-90	SSEWqFPFF	SPMFVA	PTYYPSL	0.641	±	0.024	0.926	±	0.149	0.636	±	0.088
H123#312	SSEITFPFF	SPMFAq	PSYLPSL	0.825	±	0.043	0.926	±	0.384	0.977	±	0.101
H123#228	SSELGFPMF	SPMFqH	PSYLPSG	0.483	±	0.006	0.925	±	0.379	1.109	±	0.116
3rd_0.5-2-1A	SSEVqFPFF	SPMFNH	PTYYSDM	0.641	±	0.021	0.924	±	0.386	0.933	±	0.130
1026R69	SSELqFPFF	SPMFAW	PSYLPSQ	0.815	±	0.048	0.923	±	0.292	0.825	±	0.075
H123#171	SSEITFPqF	SPMFSL	PSYLPRH	0.503	±	0.012	0.920	±	0.260	1.025	±	0.136
H123#221	SSETMFPYF	SPMFEF	PSYRPNS	0.766	±	0.050	0.920	±	0.381	0.647	±	0.072
1026B106	SSEHLFPYF	SPMFYW	PTYAPSq	0.392	±	0.020	0.919	±	0.427	1.228	±	0.112
1st-F10-0.1-10B	SSELMFPMF	SPMFQq	PSYLPSN	0.970	±	0.059	0.917	±	0.321	0.706	±	0.190
1026B16	SSEFqFPYF	SPMFLL	PTYLPSQ	0.791	±	0.034	0.917	±	0.154	0.787	±	0.085
3rd_0.5-1-09E	SSEVVFPLF	SPMFAq	PSYWPSA	0.362	±	0.006	0.916	±	1.018	1.039	±	0.151
1011-55	SSELSFPYF	SPMFSH	PTYYPSE	0.862	±	0.048	0.915	±	0.133	1.869	±	0.286
H123#262	SSELMFPYF	SPMFTq	PSYLASP	0.706	±	0.013	0.914	±	0.387	0.744	±	0.066
3rd_0.5-0.25-12D	SSELqFPFF	SPMFPR	PGYKPSA	0.586	±	0.005	0.912	±	0.267	0.890	±	0.066
H123#206	SSELLFPFF	SPMFVq	PTYQPSM	0.598	±	0.004	0.911	±	0.255	1.261	±	0.145
1101-73	SSELTFPFF	SPMFAS	PYYVPSY	0.659	±	0.011	0.908	±	0.186	1.101	±	0.090
H123#2	SSELMFPHF	SPMFSY	PSYNVTq	0.591	±	0.037	0.907	±	0.315	0.241	±	0.020
1101-28	SSELMFPMF	SPMFGY	PHYRNPV	0.700	±	0.018	0.905	±	0.177	0.948	±	0.082
1101-124	SSELLFPNF	SPMFqF	PAYTTRG	0.561	±	0.028	0.903	±	0.425	1.045	±	0.240
3RD-1-6B	SSEVqFPFF	SPMFHL	PHYLPSE	0.928	±	0.029	0.903	±	0.175	0.549	±	0.061
1026B103	SSELqFPFF	SPMFAK	PTYYVTP	0.408	±	0.016	0.903	±	0.591	1.265	±	0.101
H123#60	SSELqFPSF	SPMFNQ	PGYLPRD	0.366	±	0.022	0.895	±	1.088	0.602	±	0.057
1026R63	SSEWLFPYF	SPMFSL	PTYSPSS	0.822	±	0.005	0.894	±	0.299	0.986	±	0.067
3rd_0.5-0.25-09F	SSELqFPFF	SPMFNV	PAYVPRF	0.772	±	0.050	0.891	±	0.280	0.999	±	0.154

H123#157	SSEKLFPYF	SPMFTL	PTYLPSA	0.713	±	0.018	0.889	±	0.134	1.583	±	0.171
1101-189	SSEIHFPAF	SPMFAq	PTYYPMP	0.365	±	0.019	0.889	±	0.965	0.868	±	0.137
3rd_0.5-0.0625-07F	SSELTFPGF	SPMFqR	PVYFNQG	0.420	±	0.003	0.887	±	0.756	0.863	±	0.173
3RD-1-1A	SSELMFPYF	SPMFAq	PSYNVQG	0.523	±	0.003	0.887	±	0.551	1.701	±	0.221
3rd_1-0.0625-G-1	SSELTFPFF	SPMFSR	PRYLPSH	1.060	±	0.048	0.882	±	0.161	0.741	±	0.089
1101-19	SSELLFPFF	SPMFQq	PTYYFSF	0.327	±	0.005	0.882	±	0.740	1.505	±	0.128
1026B37	SSEITFPYF	SPMFqY	PTYWTSN	0.713	±	0.011	0.878	±	0.286	0.617	±	0.054
3rd_1-2-E-07	SSEYMFPYF	SPMFqH	PLYSSRG	0.565	±	0.020	0.875	±	0.230	0.828	±	0.075
3rd-0.5-A9	SSELTFPFF	SPMFRq	PTYYTTY	1.023	±	0.024	0.873	±	0.301	0.511	±	0.084
H123#298	SSEMVFPYF	SPMFNL	PSYKASA	0.554	±	0.005	0.871	±	0.450	1.088	±	0.097
3rd_0.5-2-5G	SSELTFPSF	SPMFSK	PTYRPSF	0.319	±	0.002	0.871	±	1.260	1.196	±	0.198
H123#141	SSELNFPFF	SPMFQH	PSYNVTP	0.641	±	0.005	0.870	±	0.164	1.649	±	0.163
H123#272	SSELMFPSF	SPMFqM	PGYIIGS	0.393	±	0.010	0.869	±	0.580	0.601	±	0.059
1026R58	SSEWTFPYF	SPMFLK	PQYVPSP	0.523	±	0.013	0.867	±	0.602	1.292	±	0.094
H123#319	SSEVLFPFF	SPMFQV	PWYFNHG	0.332	±	0.006	0.863	±	0.781	1.598	±	0.146
3rd-0.25-10A	SSEVTFPFF	SPMFQq	PSYLPQN	1.035	±	0.043	0.863	±	0.300	0.448	±	0.038
1101-40	SSEWqFPAF	SPMFQL	PSYLPRG	0.371	±	0.002	0.856	±	0.577	1.384	±	0.122
3rd_1-2-D-09	SSEFLFPFF	SPMFqF	PFYKEAG	0.629	±	0.025	0.852	±	0.235	1.050	±	0.099
H123#93	SSEVLFPAF	SPMFAT	PTYYPSS	1.283	±	0.022	0.851	±	0.101	0.868	±	0.060
1026R46	SSELTFPYF	SPMFGL	PTYITSq	0.824	±	0.033	0.850	±	0.268	0.653	±	0.050
3rd_0.5-0.25-09D	SSELSFPSF	SPMFNL	PTYLPTS	1.241	±	0.099	0.849	±	0.171	0.862	±	0.090
2D-0.5-8F	SSEHVFPLF	SPMFqF	PTYLASP	0.617	±	0.014	0.847	±	0.371	0.754	±	0.089
H123#152	SSELWFPFF	SPMFqL	PRYKTTG	0.441	±	0.020	0.842	±	0.263	1.533	±	0.173
H123#472	SSEVTFPMF	SPMFSN	PSYFASP	0.285	±	0.000	0.841	±	0.571	1.855	±	0.333
3rd_0.5-0.0625-11A	SSELqFPSF	SPMFEY	PTYRPSY	0.538	±	0.021	0.837	±	0.340	0.865	±	0.063
1011-15	SSEVNFPqF	SPMFSY	PTYLARD	0.775	±	0.041	0.836	±	0.227	0.719	±	0.112
3rd_1-0.0625-B-5	SSEITFPYF	SPMFqY	PLYLPSP	1.152	±	0.044	0.834	±	0.137	0.928	±	0.183
H123#81	SSEFTFPFF	SPMFDq	PRYYPTS	0.993	±	0.009	0.831	±	0.189	0.985	±	0.085
3rd-0.25-12D	SSEITFPFF	SPMFNT	PTYTNSY	0.835	±	0.033	0.830	±	0.286	0.553	±	0.047
1101-145	SSELMFPMF	SPMFqT	PVYKVSA	0.331	±	0.019	0.830	±	1.138	1.005	±	0.278
3rd_1-0.0625-H-2	SSEVTFPHF	SPMFAq	PTYLTSM	0.895	±	0.027	0.827	±	0.246	0.436	±	0.049
3rd_0.5-0.0625-08E	SSEMTFPFF	SPMFHY	PTYYTVD	0.405	±	0.006	0.826	±	0.696	1.070	±	0.188
1101-29	SSELqFPYF	SPMFNI	PTYYASN	0.657	±	0.061	0.825	±	0.270	0.912	±	0.108
H123#290	SSELTFPYF	SPMFHL	PDYKAHG	0.259	±	0.005	0.823	±	1.697	1.842	±	0.176
1011-45	SSEVTFSSF	SPMFPN	PSYHPTN	0.964	±	0.007	0.821	±	0.099	0.915	±	0.132
3rd_0.5-4-07C	SSELMFPSF	SPMFqQ	PQYLMGP	0.720	±	0.029	0.820	±	0.245	0.478	±	0.092
3rd_0.5-0.25-11D	SSEVLFPFF	SPMFNR	PSYYPSK	1.430	±	0.015	0.815	±	0.150	0.628	±	0.039
H123#424	SSEVNFPYF	SPMFNL	PTYRPGE	0.683	±	0.045	0.815	±	0.216	0.688	±	0.078
1st-F10-0.1-9G	SSELTFPYF	SPMFYS	PSYRESG	0.655	±	0.023	0.813	±	0.359	0.562	±	0.034
3RD-1-5B	SSELqFPYF	SPMFNR	PLYMTRG	0.950	±	0.102	0.810	±	0.194	0.638	±	0.098
3rd_1-0-G-9	SSELMFPYF	SPMFNS	PTYHTKN	0.991	±	0.030	0.809	±	0.145	0.605	±	0.068
H123#140	SSEWMFPYF	SPMFNA	PSYVPIP	0.658	±	0.038	0.808	±	0.180	1.302	±	0.148
3rd_0.5-1-08D	SSEFLFPqF	SPMFAH	PTYFPSG	0.751	±	0.030	0.807	±	0.246	1.014	±	0.086
3rd_0.5-0.0625-12C	SSEWqFPYF	SPMFQL	PTYMQSF	0.668	±	0.064	0.807	±	0.328	1.157	±	0.127
3rd_0.5-0.0625-10E	SSEFLFPFF	SPMFqR	PSYSRDS	0.639	±	0.026	0.806	±	0.387	0.945	±	0.139
3rd_0.5-0.125-6B	SSELLFPFF	SPMFQT	PHYHLSS	1.167	±	0.017	0.806	±	0.178	0.757	±	0.047
3rd_0.5-0.25-08C	SSELqFPHF	SPMFEL	PQYRPTP	0.364	±	0.008	0.806	±	0.204	1.302	±	0.125
3RD-1-4F	SSEYLFPFF	SPMFNL	PSYKPNL	0.665	±	0.11	0.805	±	0.254	0.790	±	0.149
3rd_1-2-G-09	SSELLFPFF	SPMFRI	PTYAPVG	0.759	±	0.013	0.802	±	0.350	1.312	±	0.102

2D-0.5-9H	SSEqLFPYF	SPMFSW	PQYTSSG	1.123	±	0.023	0.801	±	0.144	0.551	±	0.104
H123#454	SSEMLFPMF	SPMFGH	PQYAPRG	0.395	±	0.029	0.799	±	0.237	1.457	±	0.163
3rd_0.5-4-12E	SSEVTFPLF	SPMFPq	PLYRVRG	0.525	±	0.005	0.799	±	0.603	0.984	±	0.140
H123#196	SSEVTFPFF	SPMFqY	PTYKPGL	0.823	±	0.037	0.793	±	0.124	1.095	±	0.122
1101-102	SSEWKFPYF	SPMFqL	PTYTSKG	0.310	±	0.008	0.793	±	1.118	1.241	±	0.314
3rd-0.25-10G	SSELTFPHF	SPMFVL	PSYLPGH	0.965	±	0.090	0.792	±	0.298	0.501	±	0.053
H123#191	SSEVTFPFF	SPMFqL	PSYPPSF	0.669	±	0.032	0.792	±	0.144	0.862	±	0.093
H123#326	SSEMTFPSF	SPMFNq	PSYTRPN	0.365	±	0.002	0.791	±	0.159	0.395	±	0.029
1st-F10-0.1-4E	SSELIFPVF	SPMFPP	PTYYDPP	0.457	±	0.020	0.787	±	0.360	0.854	±	0.057
3rd_0.5-0.125-4H	SSELqFPAF	SPMFPR	PTYRPSP	0.527	±	0.023	0.787	±	0.229	1.185	±	0.087
3rd_1-2-A-07	SSEMLFPAF	SPMFRV	PqYYPSP	0.190	±	0.015	0.786	±	1.062	2.467	±	0.290
1st-F10-0.1-11E	SSELLFPYF	SPMFAq	PSYNTTA	0.724	±	0.062	0.784	±	0.320	0.646	±	0.081
1011-3	SSELVFPLF	SPMFqT	PEYRPMP	0.531	±	0.041	0.778	±	0.255	1.154	±	0.188
3rd_0.5-2-4D	SSEFNFPMF	SPMFQq	PHYMTPI	0.360	±	0.013	0.776	±	1.251	1.425	±	0.216
H123#292	SSEVqFPFF	SPMFGN	PLYYPME	1.057	±	0.110	0.775	±	0.325	0.688	±	0.093
3rd_1-0-B-8	SSEITFPHF	SPMFqL	PMYYPTQ	1.250	±	0.061	0.775	±	0.129	0.829	±	0.099
1011-52	SSEHLFPYF	SPMFSR	PTYLPSE	1.077	±	0.062	0.773	±	0.113	1.319	±	0.203
3rd_0.5-0.0625-07E	SSELWFPFF	SPMFPq	PTYRSTP	0.422	±	0.003	0.773	±	0.344	0.488	±	0.068
H123#214	SSEVTFPMF	SPMFqT	PRYTPDS	0.757	±	0.038	0.772	±	0.143	0.790	±	0.086
1101-135	SSELLFPFF	SPMFHP	PFYLPSP	0.731	±	0.062	0.771	±	0.259	1.104	±	0.205
1101-76	SSEFMFPAF	SPMFQq	PAYVPSG	0.359	±	0.002	0.770	±	0.326	1.358	±	0.148
H123#108	SSEIMFPLF	SPMFQH	PTYISqG	0.333	±	0.004	0.768	±	1.008	2.203	±	0.318
H123#348	SSELTFPYF	SPMFMN	PMYLPRG	0.227	±	0.003	0.767	±	1.532	1.544	±	0.149
1026B116	SSELTFPLF	SPMFqV	PTYYHTS	0.573	±	0.014	0.767	±	0.273	0.777	±	0.082
H123#200	SSELLFPYF	SPMFWS	PSYRATP	0.537	±	0.016	0.761	±	0.186	1.134	±	0.123
2D-0.5-12C	SSELWFPAF	SPMFqR	PTYLPTH	0.157	±	0.004	0.758	±	1.416	2.281	±	0.250
3rd-0.25-8H	SSELSFPFF	SPMFQq	PTYQPGM	0.610	±	0.102	0.757	±	0.332	1.013	±	0.180
1026B129	SSELMFPSF	SPMFqI	PTYYASS	0.951	±	0.018	0.752	±	0.247	0.676	±	0.051
1011-28	SSELTFPFF	SPMFqR	PLYIANE	0.398	±	0.014	0.750	±	0.263	1.349	±	0.220
1st-F10-0.1-5B	SSELMFPYF	SPMFSL	PNYLTSP	1.242	±	0.057	0.748	±	0.259	0.510	±	0.039
2nd-10E	SSEVTFPMF	SPMFGL	PVYMPME	1.155	±	0.073	0.747	±	0.265	0.686	±	0.072
3rd_0.5-0.0625-07G	SSEFMFPSF	SPMFAq	PTYFPST	0.507	±	0.014	0.746	±	0.509	1.203	±	0.072
2ND-0.5-F10-0-A5	SSELVFPFF	SPMFqQ	PTYNISN	1.058	±	0.024	0.745	±	0.261	0.623	±	0.072
2ND-0.5-F10-0-C1	SSEMYFPRF	SPMFYH	PTYIPSL	0.532	±	0.049	0.745	±	0.275	0.739	±	0.098
3rd-0.5-A8	SSELLFPFF	SPMFNq	PRYYTTH	1.222	±	0.027	0.744	±	0.256	0.442	±	0.092
H123#85	SSEWMFPLF	SPMFPS	PTYYTSN	0.591	±	0.005	0.744	±	0.705	1.059	±	0.069
1101-17	SSELLFPFF	SPMFqK	PQYQKTG	0.320	±	0.005	0.743	±	0.338	1.367	±	0.130
3rd_0.5-1-10D	SSETLFPLF	SPMFHH	PEYLPTD	0.536	±	0.116	0.743	±	0.537	0.880	±	0.227
H123#114	SSELTFPFF	SPMFqN	PSYSVKP	0.918	±	0.076	0.742	±	0.117	0.741	±	0.122
H123#102	SSELLFPYF	SPMFqA	PSYKPTM	1.121	±	0.048	0.740	±	0.114	0.995	±	0.147
H123#213	SSELSFPAF	SPMFPT	PQYRPTQ	0.821	±	0.027	0.739	±	0.121	0.980	±	0.111
1101-165	SSELTFPFF	SPMFPT	PTYLSRS	0.461	±	0.011	0.737	±	0.483	1.009	±	0.203
1101-56	SSELNFPSF	SPMFqL	PTYRPSN	0.497	±	0.006	0.737	±	0.545	1.066	±	0.182
1026B102	SSELLFPFF	SPMFSS	PSYSPSN	0.746	±	0.005	0.732	±	0.235	0.866	±	0.056
3rd_0.5-1-07H	SSEVTFPLF	SPMFqT	PTYSPSL	1.106	±	0.05	0.730	±	0.186	0.545	±	0.040
H123#151	SSELVFPSF	SPMFSR	PQYYPSH	0.675	±	0.036	0.729	±	0.109	1.206	±	0.142
1026R76	SSELLFPMF	SPMFNV	PRYFPDS	0.835	±	0.004	0.726	±	0.244	0.904	±	0.062
H123#178	SSEIHFPqF	SPMFQF	PTYLPTF	0.371	±	0.018	0.721	±	0.680	1.290	±	0.139
3rd-0.25-10E	SSEILFPYF	SPMFGT	PTYYLqP	1.278	±	0.029	0.720	±	0.247	0.530	±	0.033

3rd_0.5-4-11E	SSELTFPYF	SPMFGR	PTYTPAA	1.294	±	0.019	0.718	±	0.146	0.743	±	0.104
H123#285	SSEVTFSSF	SPMFqV	PAYLRPT	0.415	±	0.042	0.717	±	0.405	1.096	±	0.163
H123#131	SSESTFPMF	SPMFqV	PSYIRGS	0.875	±	0.063	0.716	±	0.119	1.334	±	0.160
1026B46	SSELMFPYF	SPMFqQ	PTYVPTG	0.302	±	0.001	0.714	±	0.432	2.701	±	0.220
H123#336	SSEITFPGF	SPMFPP	PSYLPRG	0.224	±	0.003	0.714	±	0.921	2.192	±	0.202
H123#136	SSELTFPMF	SPMFqW	PVYTPTF	0.755	±	0.103	0.712	±	0.190	0.962	±	0.162
3rd_1-4-C-05	SSEIVFPFF	SPMFqI	PQYRPTP	0.885	±	0.097	0.712	±	0.197	0.792	±	0.114
H123#244	SSEIAFPFF	SPMFST	PAYRASG	0.385	±	0.006	0.711	±	0.413	0.594	±	0.054
1101-191	SSELAFPFF	SPMFqW	PQYVPRA	0.305	±	0.008	0.709	±	0.930	1.535	±	0.385
H123#70	SSELTFPYF	SPMFSN	PNYTVDS	0.337	±	0.003	0.708	±	1.049	0.982	±	0.073
1011-54	SSEIGFPSF	SPMFNH	PTYLPSS	1.157	±	0.039	0.704	±	0.090	1.103	±	0.165
H123#466	SSELLFPFF	SPMFSN	PLYRTAG	1.103	±	0.086	0.703	±	0.104	0.922	±	0.150
3rd_0.5-1-10F	SSELLFPYF	SPMFNT	PSYSVSN	1.509	±	0.061	0.701	±	0.122	0.700	±	0.099
H123#173	SSEVTFPYF	SPMFqS	PTYNPAA	0.504	±	0.018	0.698	±	0.291	0.710	±	0.081
1011-41	SSELqFPAF	SPMFPN	PGYQPSH	0.398	±	0.008	0.696	±	0.508	1.457	±	0.215
3rd_0.5-0.125-1H	SSELTFPSF	SPMFqI	PSYLVPY	0.314	±	0.005	0.696	±	0.763	0.996	±	0.088
3rd_0.5-0.125-3H	SSEYHFPFF	SPMFHH	PTYVTKG	0.413	±	0.009	0.695	±	0.744	1.257	±	0.111
3rd-0.5-B10	SSEWSFPYF	SPMFqQ	PTYRASP	1.241	±	0.058	0.692	±	0.239	0.436	±	0.034
1026B56	SSELMFPSF	SPMFQq	PTYIQNA	0.307	±	0.032	0.691	±	1.411	2.364	±	0.339
H123#347	SSELTFPHF	SPMFNq	PTYVATP	0.600	±	0.027	0.687	±	0.361	1.023	±	0.086
3rd_0.5-1-08E	SSELSFPMF	SPMFQI	PQYVqAS	0.439	±	0.005	0.686	±	0.450	0.955	±	0.098
H123#161	SSEATFPLF	SPMFNK	PVYYPSG	0.720	±	0.060	0.686	±	0.223	0.650	±	0.084
1101-171	SSELSFPYF	SPMFqA	PSYMPRP	0.342	±	0.007	0.686	±	0.494	0.327	±	0.061
H123#255	SSEVTFSSF	SPMFGT	PTYLRSH	0.781	±	0.012	0.683	±	0.386	0.569	±	0.054
H123#409	SSEVLFPFF	SPMFqR	PSYMLPK	0.481	±	0.033	0.683	±	0.492	1.467	±	0.148
1101-97	SSELLFPMF	SPMFqR	PSYLTTM	0.335	±	0.012	0.683	±	0.632	0.748	±	0.150
3rd-1-G2	SSELLFPFF	SPMFqS	PSYLRAS	1.069	±	0.041	0.683	±	0.242	0.404	±	0.035
H123#227	SSELLFPFF	SPMFPS	PRYSTRG	0.316	±	0.000	0.680	±	0.485	1.714	±	0.158
3rd-0.5-D11	SSELLFPYF	SPMFqL	PSYMADN	1.293	±	0.031	0.678	±	0.232	0.337	±	0.022
H123#53	SSEIqFPSF	SPMFNF	PEYRTSA	0.478	±	0.009	0.677	±	0.666	0.429	±	0.029
H123#34	SSEEQFPVF	SPMFSS	PTYTPRG	0.323	±	0.001	0.676	±	1.330	0.541	±	0.038
H123#321	SSELVFPFF	SPMFAq	PTYRASA	0.502	±	0.005	0.674	±	0.408	1.168	±	0.111
1026R47	SSEVTFPFF	SPMFPL	PRYHITP	1.125	±	0.026	0.667	±	0.206	0.527	±	0.038
3rd_0.5-0.5-6G	SSEFFFPYF	SPMFKq	PSYVPSS	0.489	±	0.018	0.666	±	0.264	1.359	±	0.091
H123#258	SSELTFPYF	SPMFqL	PTYLPSD	1.174	±	0.049	0.665	±	0.272	0.647	±	0.063
3rd_0.5-1-11D	SSEYLFPLF	SPMFSH	PAYLPSP	0.667	±	0.037	0.664	±	0.531	0.941	±	0.138
3rd_0.5-0.5-4E	SSEVNFPYF	SPMFMq	PTYKPTY	0.721	±	0.035	0.663	±	0.242	0.560	±	0.085
3rd-1-G6	SSELVFPYF	SPMFFq	PSYFPSA	0.870	±	0.029	0.657	±	0.286	0.435	±	0.030
H123#4	SSEFMFPSF	SPMFSD	PLYITRG	1.493	±	0.25	0.650	±	0.176	0.309	±	0.055
H123#370	SSELTFPYF	SPMFTH	PYYVPTA	0.716	±	0.083	0.649	±	0.161	0.726	±	0.099
1st-F10-0.1-4B	SSELSFPSF	SPMFDT	PTYYATN	0.491	±	0.021	0.649	±	0.303	0.682	±	0.045
3rd_0.5-0.0625-07C	SSELqFPFF	SPMFQL	PGYLRAK	0.410	±	0.003	0.647	±	0.628	0.710	±	0.144
1101-52	SSELqFPFF	SPMFPQ	PLYFPME	0.626	±	0.022	0.645	±	0.292	0.588	±	0.147
H123#415	SSELSFPFF	SPMFWH	PYYVPRE	0.381	±	0.028	0.645	±	0.550	1.667	±	0.175
H123#177	SSEVTFPNF	SPMFNL	PTYISSH	0.947	±	0.121	0.643	±	0.157	0.821	±	0.133
3rd_1-0-C-8	SSEMSFPFF	SPMFPq	PTYMPAL	1.257	±	0.011	0.641	±	0.104	0.765	±	0.085
3rd_0.5-0.0625-08F	SSEVqFPFF	SPMFPI	PRYRNGS	0.402	±	0.004	0.640	±	1.011	0.501	±	0.110
H123#236	SSEWqFPFF	SPMFNF	PSYNIRG	0.379	±	0.005	0.639	±	0.640	0.498	±	0.045
3RD-1-3F	SSENVFPFF	SPMFqF	PTYTPSP	1.295	±	0.098	0.638	±	0.175	0.323	±	0.043

3rd_0.5-4-09C	SSELAFPYF	SPMFAq	PTYKPAM	0.737	±	0.015	0.629	±	0.467	0.974	±	0.137
1101-71	SSELNFPMF	SPMFQH	PTYMPST	0.971	±	0.032	0.629	±	0.120	0.668	±	0.061
H123#420	SSELTFPLF	SPMFQH	PYYLVSE	0.463	±	0.015	0.628	±	0.482	1.291	±	0.130
3rd_0.5-0.0625-10F	SSELqFPYF	SPMFAI	PSYLWSH	0.680	±	0.030	0.628	±	0.565	0.442	±	0.067
1101-30	SSELqFPYF	SPMFLH	PTYLPTA	0.389	±	0.004	0.625	±	0.402	1.253	±	0.137
3rd_0.5-0.25-09E	SSELqFPFF	SPMFNV	PGYIRPS	0.820	±	0.039	0.622	±	0.206	0.871	±	0.066
3rd-0.25-11A	SSEFLFPYF	SPMFAq	PFYHQQG	0.969	±	0.051	0.622	±	0.234	0.638	±	0.078
3RD-1-2E	SSELFFPFF	SPMFLq	PSYSPSH	0.417	±	0.002	0.621	±	0.343	0.813	±	0.089
H123#332	SSEMSFPFF	SPMFqN	PSYYLSG	0.244	±	0.005	0.621	±	1.720	1.636	±	0.121
H123#365	SSEATFPFF	SPMFDI	PSYRPSN	0.994	±	0.144	0.620	±	0.120	0.560	±	0.091
1026B24	SSELqFPFF	SPMFPQ	PVYYPSY	0.559	±	0.046	0.617	±	0.321	1.173	±	0.141
1026R75	SSEVAFPFF	SPMFPq	PTYYPTS	1.264	±	0.035	0.616	±	0.192	0.596	±	0.044
H123#333	SSETTFPHF	SPMFAq	PTYMPRH	0.573	±	0.020	0.616	±	0.463	0.551	±	0.043
3rd_0.5-0.5-6F	SSELqFPFF	SPMFAL	PTYRSSH	0.574	±	0.043	0.616	±	0.381	1.015	±	0.112
1st-F10-0.1-9H	SSELDFPFF	SPMFqV	PSYIYGA	1.184	±	0.089	0.613	±	0.217	0.485	±	0.050
H123#269	SSEIMFPYF	SPMFGq	PYYSSSP	0.636	±	0.018	0.613	±	0.254	0.490	±	0.044
1011-12	SSEWqFPYF	SPMFNL	PTYHADH	0.592	±	0.028	0.612	±	0.220	1.079	±	0.173
1st-F10-0.1-10F	SSEINFPFF	SPMFqS	PSYLPES	0.669	±	0.029	0.611	±	0.391	0.622	±	0.065
H123#198	SSEMNFPFF	SPMFKq	PAYRVNS	0.615	±	0.013	0.610	±	0.144	0.326	±	0.034
H123#264	SSEVGFPFF	SPMFqV	PTYYLPG	0.640	±	0.013	0.609	±	0.319	0.565	±	0.048
H123#59	SSELTFPLF	SPMFMq	PTYHPRS	0.311	±	0.004	0.608	±	1.402	1.282	±	0.101
H123#428	SSEFMFPSF	SPMFNH	PSYKYAS	0.471	±	0.001	0.606	±	0.298	0.673	±	0.078
1011-48	SSEFqFPYF	SPMFGH	PYYYATH	0.574	±	0.002	0.606	±	0.172	1.071	±	0.152
3rd_0.5-0-4G	SSEMTFPFF	SPMFQA	PTYSATK	0.905	±	0.032	0.604	±	0.174	0.803	±	0.052
1011-25	SSEPTFPHF	SPMFqL	PTYFITN	1.322	±	0.118	0.603	±	0.091	0.485	±	0.089
H123#39	SSEVFFPYF	SPMFGR	PSYYLTN	0.809	±	0.025	0.602	±	0.122	0.585	±	0.053
2D-0.5-8G	SSEMFFPqF	SPMFNL	PTYYFTG	0.606	±	0.026	0.601	±	0.551	0.461	±	0.053
3rd_1-0.0625-H-3	SSELMFPYF	SPMFqA	PTYSPSN	1.697	±	0.039	0.599	±	0.100	0.386	±	0.050
3rd-0.5-E10	SSEITFPMF	SPMFqI	PQYSSSP	1.410	±	0.064	0.597	±	0.211	0.510	±	0.041
3rd_0.5-0-2E	SSEVYFPFF	SPMFSV	PRYLNSP	0.405	±	0.013	0.596	±	0.945	0.710	±	0.102
3rd_0.5-0.0625-12E	SSELVFPYF	SPMFNq	PLYTPTH	0.768	±	0.083	0.596	±	0.507	0.634	±	0.094
1st-F10-0.1-8B	SSELFFPMF	SPMFMH	PTYQAGP	0.647	±	0.007	0.595	±	0.389	0.560	±	0.030
1101-65	SSEFTFPFF	SPMFNN	PSYMYPV	0.544	±	0.028	0.594	±	0.403	0.874	±	0.086
H123#444	SSEVTFSSF	SPMFqT	PSYKPVE	0.485	±	0.133	0.593	±	0.237	0.624	±	0.180
1101-175	SSEVTFSSF	SPMFqR	PSYLPAD	0.303	±	0.005	0.589	±	1.748	1.165	±	0.294
3rd_1-2-D-10	SSEILFPFF	SPMFMN	PAYRPTN	0.916	±	0.063	0.588	±	0.178	0.631	±	0.072
H123#74	SSEWSFPYF	SPMFQY	PTYTPTT	2.156	±	0.003	0.584	±	0.059	0.667	±	0.056
3rd_0.5-0.125-2G	SSELTFPFF	SPMFHV	PTYYPTQ	0.306	±	0.001	0.583	±	0.505	0.864	±	0.112
H123#238	SSELLFPFF	SPMFMT	PRYYLNH	0.700	±	0.067	0.582	±	0.310	0.898	±	0.116
1st-F10-0.1-2E	SSELTFPFF	SPMFGT	PGYIVPL	0.443	±	0.010	0.579	±	0.662	0.577	±	0.034
3rd_0.5-0.125-2E	SSELPFPYF	SPMFPT	PTYNSTN	0.339	±	0.004	0.579	±	0.573	1.383	±	0.111
3rd_0.5-2-4G	SSEILFPMF	SPMFGY	PDYIqSA	0.378	±	0.012	0.578	±	1.179	1.978	±	0.326
H123#232	SSEFLFPYF	SPMFNL	PCYGPAC	0.527	±	0.016	0.577	±	0.351	0.799	±	0.078
1011-37	SSEIVFPLF	SPMFqH	PTYWPPN	0.477	±	0.016	0.577	±	0.357	1.185	±	0.184
1026B45	SSELLFPAF	SPMFGq	PTYRPNP	0.328	±	0.007	0.577	±	1.379	1.807	±	0.162
3rd_0.5-0.25-10G	SSEFMFPYF	SPMFqL	PSYLMAD	1.184	±	0.017	0.567	±	0.286	0.597	±	0.038
1026R77	SSELVFPFF	SPMFqH	PSYSPDS	1.073	±	0.019	0.567	±	0.194	0.531	±	0.038
H123#111	SSEILFPHF	SPMFDH	PRYYPSG	1.440	±	0.043	0.561	±	0.077	0.719	±	0.104
3rd_0.5-0-1C	SSELNFPIF	SPMFHY	PGYNVTG	0.332	±	0.002	0.560	±	0.651	1.574	±	0.105

H123#146	SSELLFPMF	SPMFqR	PHYMHSK	0.810	±	0.006	0.560	±	0.081	0.961	±	0.139
3rd_0.5-0.5-5G	SSEWqFPYF	SPMFTS	PTYYSNA	0.731	±	0.091	0.557	±	0.617	0.878	±	0.130
3rd_0.5-0.25-12B	SSEIVFPNF	SPMFPq	PSYLPTH	0.972	±	0.036	0.553	±	0.180	0.505	±	0.043
1011-11	SSEWqFPFF	SPMFSN	PFYYLTE	0.859	±	0.024	0.548	±	0.156	0.829	±	0.119
3rd_1-2-D-08	SSEVqFPFF	SPMFSR	PTYYPNP	0.593	±	0.072	0.547	±	0.339	0.633	±	0.094
H123#270	SSEILFPYF	SPMFGq	PHYLITH	0.473	±	0.036	0.547	±	0.307	0.457	±	0.052
H123#363	SSEVGFPSF	SPMFPS	PSYRPTF	0.228	±	0.003	0.546	±	0.587	0.643	±	0.048
H123#117	SSELTFPLF	SPMFHT	PNYKPTS	0.781	±	0.107	0.540	±	0.191	0.564	±	0.116
H123#313	SSEFAFPFF	SPMFQS	PAYSRSH	0.293	±	0.015	0.537	±	1.152	0.551	±	0.056
H123#210	SSEINFPFF	SPMFHq	PSYFTDG	0.486	±	0.021	0.532	±	0.154	1.841	±	0.197
H123#278	SSEVNFPAF	SPMFPq	PSYRPTY	0.581	±	0.045	0.527	±	0.233	0.682	±	0.078
1011-404	SSELLFPYF	SPMFHq	PGYRPDM	1.312	±	0.073	0.520	±	0.163	0.533	±	0.058
3rd-0.25-9D	SSEPTFPMF	SPMFqH	PLYRTQG	1.036	±	0.043	0.518	±	0.196	0.313	±	0.022
3rd_0.5-0.125-3D	SSELqFPFF	SPMFAL	PHYTRSA	0.557	±	0.029	0.518	±	0.715	1.103	±	0.205
H123#449	SSEVNFPMF	SPMFPI	PTYYINQ	1.101	±	0.067	0.515	±	0.138	0.489	±	0.051
H123#280	SSELQFPNF	SPMFqY	PRYISKG	0.408	±	0.014	0.515	±	0.256	1.038	±	0.101
3rd_1-2-A-08	SSEFqFPHF	SPMFGY	PTYRPSS	0.610	±	0.094	0.513	±	0.389	0.875	±	0.154
3rd_0.5-0.5-5A	SSEqLFPYF	SPMFQR	PSYIPSN	1.651	±	0.024	0.508	±	0.103	0.368	±	0.020
H123#169	SSEWSFPFF	SPMFqN	PQYLVMP	0.792	±	0.025	0.506	±	0.097	0.680	±	0.075
1026B126	SSEITFPFF	SPMFAq	PAYTASP	1.370	±	0.038	0.505	±	0.159	0.348	±	0.038
1026B92	SSELLFPSF	SPMFqM	PTYYPVP	0.436	±	0.034	0.505	±	0.377	1.311	±	0.148
H123#65	SSEVLFPYF	SPMFHR	PQYVSTP	0.703	±	0.080	0.504	±	0.445	0.620	±	0.103
1026R72	SSELTFPYF	SPMFqT	PTYAPSQ	0.962	±	0.088	0.502	±	0.225	0.577	±	0.073
1011-402	SSEHTFPYF	SPMFAq	PTYVPSQ	0.459	±	0.030	0.502	±	0.358	0.862	±	0.101
2ND-0.5-F10-0.0625-E4	SSELVFPFF	SPMFNH	PTYSIAH	0.542	±	0.078	0.500	±	0.234	0.782	±	0.131
1st-F10-0.1-9E	SSELqFPFF	SPMFRL	PTYRPTA	0.561	±	0.009	0.499	±	0.317	0.731	±	0.083
1011-53	SSEWqFPFF	SPMFMV	PTYLPGQ	0.859	±	0.090	0.497	±	0.226	0.711	±	0.135
H123#474	SSEVTFPYF	SPMFYT	PHYLPSS	1.643	±	0.081	0.496	±	0.064	0.362	±	0.054
2D-0.5-9F	SSEVqFPFF	SPMFGT	PQYLPSS	1.346	±	0.071	0.493	±	0.153	0.246	±	0.030
3rd_0.5-0.0625-11H	SSEIqFPFF	SPMFNS	PTYYPLE	0.999	±	0.024	0.490	±	0.182	0.656	±	0.094
H123#122	SSELYFPKF	SPMFqL	PQYLNSP	0.498	±	0.002	0.489	±	0.115	0.716	±	0.073
H123#295	SSELLFPMF	SPMFGS	PTYLTSN	0.573	±	0.029	0.486	±	0.442	1.015	±	0.104
3RD-1-5F	SSEqLFPYF	SPMFAT	PSYITSG	0.204	±	0.003	0.484	±	1.382	0.972	±	0.088
H123#241	SSELLFPHF	SPMFGL	PSYLTSN	1.625	±	0.072	0.483	±	0.198	0.476	±	0.046
H123#68	SSEIqFPAF	SPMFNK	PSYVNPT	0.464	±	0.055	0.479	±	1.066	0.985	±	0.141
1026R84	SSELNFPFF	SPMFqW	PSYMNGG	0.689	±	0.045	0.476	±	0.478	0.800	±	0.081
H123#35	SSEIGFPYF	SPMFGF	PSYQSYA	0.906	±	0.051	0.476	±	0.254	0.268	±	0.024
1101-2	SSELqFPSF	SPMFQH	PEYIARG	0.354	±	0.006	0.474	±	0.617	1.542	±	0.136
H123#192	SSEIAFPLF	SPMFqL	PSYLPHE	0.480	±	0.019	0.472	±	0.193	1.007	±	0.329
3rd_1-0.0625-E-6	SSEMVFPFF	SPMFDM	PHYRPDH	1.032	±	0.039	0.470	±	0.221	0.508	±	0.101
3rd_1-4-H-04	SSELSFPMF	SPMFqN	PEYTHGA	0.584	±	0.011	0.467	±	0.272	0.641	±	0.094
H123#8	SSELNFPYF	SPMFAL	PSYINDG	1.076	±	0.019	0.466	±	0.401	1.093	±	0.092
3rd_0.5-1-07F	SSELMFPSF	SPMFDL	PTYHFqT	0.479	±	0.006	0.465	±	0.442	0.532	±	0.048
H123#30	SSEMHFPLF	SPMFQS	PRYMPSH	0.527	±	0.026	0.465	±	0.503	0.354	±	0.027
1011-10	SSEWLFPMF	SPMFLq	PSYQPSL	0.485	±	0.008	0.463	±	0.337	0.764	±	0.134
3rd_0.5-0-1F	SSELNFPHF	SPMFAH	PLYYPSY	0.329	±	0.001	0.463	±	0.615	1.332	±	0.074
3rd_0.5-0.25-11A	SSEFLFPMF	SPMFqT	PAYQVSP	0.925	±	0.015	0.461	±	0.270	0.725	±	0.046
2nd-0.5-F10-0.1-10F	SSELNFPqF	SPMFSH	PTYYPSN	0.712	±	0.057	0.461	±	0.541	0.798	±	0.078
3rd_0.5-0-3A	SSESAFPFF	SPMFYF	PEYIRGS	0.318	±	0.002	0.460	±	0.289	1.398	±	0.123

H123#50	SSEWRFPFF	SPMFQL	PTYRDSP	0.836	±	0.032	0.458	±	0.219	0.966	±	0.102
H123#234	SSELNFPSF	SPMFqF	PLYRPTH	0.515	±	0.012	0.457	±	0.254	0.509	±	0.046
H123#54	SSEPGFPYF	SPMFSH	PTYYTSQ	1.337	±	0.039	0.457	±	0.287	0.515	±	0.052
3rd_0.5-0.125-5B	SSELLFPAF	SPMFqQ	PRYLTTG	0.524	±	0.018	0.457	±	0.190	0.824	±	0.087
H123#314	SSEQTFPYF	SPMFAq	PTYNPRG	0.514	±	0.035	0.455	±	0.266	0.464	±	0.052
1101-78	SSEqPFPRF	SPMFPS	PTYYVNS	0.424	±	0.018	0.455	±	0.702	0.892	±	0.148
H123#18	SSEFLFPYF	SPMFLH	PGYSKPS	0.571	±	0.015	0.454	±	0.574	0.539	±	0.034
3rd_0.5-0.0625-10A	SSELSFPLF	SPMFqH	PGYLPGA	0.464	±	0.006	0.453	±	1.071	1.178	±	0.068
2ND-1-G3	SSEYLFPYF	SPMFNq	PSYIWSN	1.310	±	0.029	0.452	±	0.116	0.252	±	0.030
3rd_1-0-F-10	SSELqFPFF	SPMFPM	PHYMPTE	1.539	±	0.068	0.451	±	0.087	0.297	±	0.036
H123#149	SSEVVFPYF	SPMFqF	PKYKPTP	0.360	±	0.002	0.446	±	0.245	0.888	±	0.112
1011-14	SSEFFFPFF	SPMFAA	PTYMPTD	1.726	±	0.042	0.445	±	0.058	0.509	±	0.075
H123#94	SSEVVFPFF	SPMFNA	PRYLPGM	2.424	±	0.313	0.445	±	0.111	0.381	±	0.068
3RD-1-2C	SSEVqFPFF	SPMFYF	PTYKPSS	1.152	±	0.026	0.443	±	0.131	0.239	±	0.026
H123#168	SSEVTFPFF	SPMFAY	PFYLPNN	1.512	±	0.358	0.441	±	0.126	0.706	±	0.182
H123#155	SSEWqFPLF	SPMFQN	PTYSPFG	0.717	±	0.028	0.438	±	0.227	0.819	±	0.088
H123#42	SSELqFPMF	SPMFSW	PRYFTSG	0.332	±	0.011	0.433	±	0.478	0.832	±	0.076
H123#106	SSELSFPFF	SPMFqN	PTYTPAN	1.023	±	0.071	0.431	±	0.075	0.468	±	0.074
H123#78	SSEWSFPLF	SPMFDH	PMYFPTN	1.447	±	0.01	0.429	±	0.100	0.401	±	0.040
3rd_1-2-A-11	SSELAFPYF	SPMFqL	PSYMPKE	0.378	±	0.002	0.429	±	0.818	1.076	±	0.091
3rd_0.5-1-07D	SSEVLFPFF	SPMFPP	PVYTPSA	0.337	±	0.002	0.426	±	0.496	1.397	±	0.158
H123#137	SSEYqFPFF	SPMFPV	PTYFITN	0.991	±	0.136	0.426	±	0.148	0.412	±	0.069
3rd_1-4-E-03	SSEMTFPVF	SPMFqR	PTYVLPq	0.169	±	0.001	0.424	±	2.633	1.735	±	0.164
0.125-B4	SSELTFPMF	SPMFPT	PQYLNDY	0.370	±	0.055	0.423	±	0.761	1.617	±	0.286
3rd_0.5-0.5-5D	SSELTFPYF	SPMFRq	PTYTPTT	0.612	±	0.017	0.422	±	0.435	0.806	±	0.070
2D-0.5-8H	SSELqFPHF	SPMFRV	PQYLPTH	0.179	±	0.012	0.421	±	1.417	2.241	±	0.251
H123#129	SSELAFPLF	SPMFSN	PEYVARG	0.613	±	0.024	0.419	±	0.203	0.686	±	0.076
H123#64	SSEWqFPFF	SPMFQL	PSYLVTE	0.514	±	0.043	0.419	±	0.188	1.837	±	0.182
H123#199	SSELLFPYF	SPMFGF	PSYLVTK	1.585	±	0.027	0.417	±	0.067	0.597	±	0.074
3rd_1-0-F-8	SSEVTFPHF	SPMFGL	PTYLTNA	2.362	±	0.177	0.412	±	0.074	0.415	±	0.055
H123#144	SSEFqFPYF	SPMFHH	PTYNVNY	0.660	±	0.006	0.410	±	0.162	0.642	±	0.064
1101-98	SSEVSFPFF	SPMFqL	PAYLNGY	0.327	±	0.011	0.408	±	0.798	0.713	±	0.141
3rd_1-2-E-12	SSEIYFPFF	SPMFNS	PTYYPLE	0.857	±	0.035	0.408	±	0.214	0.500	±	0.050
3rd_1-0-D-10	SSELqFPAF	SPMFML	PTYITRA	0.745	±	0.022	0.408	±	0.455	0.899	±	0.116
1026B131	SSELSFPYF	SPMFAF	PRYKTRG	0.374	±	0.002	0.407	±	0.824	0.900	±	0.067
3rd_0.5-0-6B	SSELqFPFF	SPMFSF	PTYSTPS	0.886	±	0.044	0.407	±	0.510	0.562	±	0.086
H123#349	SSEIqFPYF	SPMFGH	PYYYIDS	0.448	±	0.069	0.405	±	0.968	0.537	±	0.092
1st-F10-0.1-11A	SSEWqFPFF	SPMFRT	PLYATRG	1.255	±	0.039	0.401	±	0.144	0.251	±	0.024
2ND-1-E2	SSEFMFPSF	SPMFPF	PTYYLqS	1.707	±	0.042	0.401	±	0.123	0.234	±	0.026
H123#1	SSEMqFPYF	SPMFGA	PTYMTTA	0.720	±	0.030	0.399	±	0.184	0.335	±	0.024
1101-69	SSELGFPFF	SPMFSL	PTYWSSG	0.360	±	0.003	0.399	±	0.659	0.800	±	0.120
H123#289	SSELSFPFF	SPMFPN	PAYTPTL	0.808	±	0.085	0.398	±	0.186	0.450	±	0.061
3rd_0.5-0.25-10F	SSELNFPYF	SPMFqV	PFYMPSD	1.191	±	0.162	0.397	±	0.134	0.487	±	0.073
1st-F10-0.1-8G	SSEWSFPqF	SPMFKL	PLYITRG	1.051	±	0.041	0.394	±	0.206	0.305	±	0.026
3rd_1-0.0625-H-5	SSEVLFPVF	SPMFqL	PTYRVSP	0.660	±	0.006	0.394	±	0.460	0.456	±	0.052
3rd_1-2-C-09	SSEqLFPYF	SPMFKH	PLYLTRG	1.172	±	0.147	0.390	±	0.142	0.407	±	0.064
H123#145	SSELPFPSF	SPMFPQ	PTYAPTD	0.709	±	0.034	0.385	±	0.330	1.012	±	0.147
H123#55	SSELqFPFF	SPMFPA	PEYRQSA	0.466	±	0.013	0.383	±	0.364	0.918	±	0.077
3rd_0.5-4-10G	SSEIQFPFF	SPMFQW	PTYIqGT	0.586	±	0.026	0.379	±	0.942	1.355	±	0.210

H123#281	SSEALFPYF	SPMFLA	PSYRPSP	0.666	±	0.015	0.376	±	0.166	0.526	±	0.045
1026B53	SSELTFPFF	SPMFqT	PSYLVGT	0.329	±	0.025	0.375	±	1.558	1.819	±	0.223
H123#150	SSELqFPLF	SPMFPS	PTYLPSS	0.578	±	0.099	0.372	±	0.313	0.672	±	0.134
H123#334	SSEITFPLF	SPMFqV	PSYSNPK	0.608	±	0.020	0.370	±	0.393	0.927	±	0.075
2nd-0.5-F10-0.1-7C	SSELLFPFF	SPMFqT	PTYNMSS	2.337	±	0.250	0.367	±	0.131	0.275	±	0.071
3rd-0.5-F10-0.0625-10G	SSEAAFPAF	SPMFqR	PRYYPTA	0.578	±	0.016	0.366	±	0.353	0.322	±	0.025
H123#84	SSEWqFPLF	SPMFNL	PVYINPG	0.686	±	0.009	0.364	±	0.192	1.135	±	0.073
1st-F10-0.1-11F	SSELqFPYF	SPMFDK	PTYLPNL	1.111	±	0.084	0.363	±	0.189	0.362	±	0.075
1011-16	SSELTFPHF	SPMFQq	PGYLHPD	0.365	±	0.009	0.362	±	0.798	1.628	±	0.242
H123#32	SSEVSFPFF	SPMFqL	PQYQVSN	1.585	±	0.208	0.362	±	0.217	0.293	±	0.046
H123#208	SSEFAFPFF	SPMFMI	PFYTPRG	0.525	±	0.017	0.355	±	0.228	0.918	±	0.097
1101-104	SSELQFPHF	SPMFqY	PRYVSTG	0.311	±	0.016	0.355	±	1.374	1.171	±	0.308
3rd_0.5-1-10A	SSELMFPMF	SPMFSS	PTYLSTA	0.895	±	0.022	0.351	±	0.170	0.570	±	0.080
H123#268	SSEYAFPAF	SPMFAF	PSYVTRG	0.478	±	0.035	0.349	±	0.328	0.619	±	0.069
H123#52	SSEVTFPLF	SPMFDL	PTYQTTM	1.508	±	0.078	0.347	±	0.358	0.341	±	0.036
1101-59	SSELqFPFF	SPMFPS	PSYRPTM	0.638	±	0.078	0.347	±	0.495	0.497	±	0.088
H123#170	SSEMGFPFF	SPMFQR	PGYIPRE	0.357	±	0.002	0.347	±	0.339	1.101	±	0.122
H123#99	SSEALFPYF	SPMFqI	PEYFQTP	2.047	±	0.065	0.346	±	0.046	0.441	±	0.089
H123#118	SSEWLFPYF	SPMFHY	PGYSPTA	2.096	±	0.678	0.344	±	0.130	0.450	±	0.161
1026B22	SSEMNFPFF	SPMFDQ	PEYMSSP	0.948	±	0.068	0.343	±	0.239	0.619	±	0.074
H123#251	SSEYMFPFF	SPMFqT	PGYITRA	0.593	±	0.010	0.340	±	0.492	0.767	±	0.067
H123#317	SSEVNFPMF	SPMFqR	PSYFYGK	0.359	±	0.002	0.340	±	1.119	1.298	±	0.108
H123#235	SSEILFPFF	SPMFNL	PDYRTSE	0.874	±	0.001	0.339	±	0.201	0.672	±	0.057
H123#134	SSELTFPqF	SPMFGI	PTYTRPA	1.046	±	0.132	0.335	±	0.108	0.458	±	0.074
H123#438	SSEIFFPFF	SPMFNT	PVYQESG	0.336	±	0.015	0.335	±	0.553	0.906	±	0.090
1026B39	SSEWqFPYF	SPMFqT	PAYLTRG	0.308	±	0.003	0.332	±	0.686	1.674	±	0.134
3rd_1-0.0625-E-4	SSELTFPMF	SPMFqH	PHYHVAS	1.050	±	0.048	0.330	±	0.062	0.406	±	0.092
1026R44	SSEWLFPYF	SPMFSL	PTYSPSS	2.351	±	0.089	0.330	±	0.102	0.234	±	0.019
H123#100	SSEYLFPAF	SPMFQL	PSYIPRD	1.621	±	0.051	0.329	±	0.072	0.447	±	0.065
H123#112	SSEALFPFF	SPMFRL	PSYRPTA	0.429	±	0.010	0.321	±	0.748	0.601	±	0.090
3rd_1-2-B-12	SSEFMFPYF	SPMFGY	PTYQPGL	1.913	±	0.063	0.321	±	0.082	0.413	±	0.038
1011-19	SSEYLFPYF	SPMFGI	PSYRPGL	2.706	±	0.138	0.318	±	0.044	0.380	±	0.058
H123#103	SSEVNFPFF	SPMFNq	PSYFPGQ	0.870	±	0.028	0.317	±	0.228	1.174	±	0.168
3rd_0.5-0.25-08F	SSEWqFPYF	SPMFSF	PTYNMTA	1.423	±	0.073	0.315	±	0.103	0.398	±	0.030
0.25-C1	SSETTFPMF	SPMFqM	PVYMRAA	0.822	±	0.007	0.309	±	0.326	0.262	±	0.030
3RD-1-5C	SSEMFFPLF	SPMFqQ	PSYLPRG	0.412	±	0.010	0.301	±	0.681	0.828	±	0.116
H123#180	SSEMTFPLF	SPMFNL	PQYNTSP	0.527	±	0.011	0.298	±	0.390	0.865	±	0.090
1101-32	SSELTFPMF	SPMFqS	PSYINAA	0.374	±	0.002	0.297	±	0.389	1.350	±	0.215
1026B48	SSELFFPMF	SPMFLT	PTYLPSA	0.439	±	0.029	0.295	±	0.422	1.494	±	0.156
0-E6	SSELqFPAF	SPMFDA	PTYVPSN	0.495	±	0.032	0.295	±	0.610	0.418	±	0.055
H123#163	SSELAFPHF	SPMFGI	PSYRPTS	1.165	±	0.104	0.294	±	0.209	0.393	±	0.052
1026R74	SSEIqFPFF	SPMFGI	PRYLPTG	1.378	±	0.046	0.294	±	0.137	0.303	±	0.079
H123#243	SSEPSFPRF	SPMFPT	PSYFPTA	0.787	±	0.058	0.293	±	0.138	0.283	±	0.032
1101-103	SSELLFPYF	SPMFKq	PGYRPSS	0.301	±	0.011	0.292	±	0.765	1.880	±	0.439
3rd_1-0.0625-C-3	SSELqFPYF	SPMFSA	PTYRVSS	0.996	±	0.046	0.292	±	0.269	0.263	±	0.033
3rd_1-0-F-7	SSEIqFPFF	SPMFQR	PTYIHPS	1.527	±	0.054	0.290	±	0.092	0.310	±	0.039
3rd-1-G4	SSEVTFPMF	SPMFQq	PRYVPLH	0.664	±	0.014	0.287	±	0.214	0.576	±	0.033
1011-32	SSEVTFPLF	SPMFQq	PSYSPGH	0.326	±	0.007	0.286	±	0.737	1.449	±	0.215
H123#442	SSEIqFPFF	SPMFYY	PFYYVHA	0.165	±	0.010	0.284	±	0.605	0.653	±	0.068

1ST-11D	SSEIqFPMF	SPMFRT	PTYLPSE	0.898	±	0.010	0.281	±	0.458	0.511	±	0.079
H123#164	SSEGSFPFF	SPMFMS	PMYKTRG	0.672	±	0.049	0.281	±	0.150	0.426	±	0.051
H123#97	SSEINFPFF	SPMFSq	PKYNVTP	0.493	±	0.026	0.280	±	0.175	2.338	±	0.464
H123#279	SSEFHFPNF	SPMFqL	PSYYPTY	0.633	±	0.013	0.279	±	0.219	1.101	±	0.094
3rd_1-0-H-9	SSELTFPqF	SPMFQL	PLYRTRG	1.570	±	0.031	0.278	±	0.084	0.332	±	0.036
H123#220	SSEMTFPYF	SPMFqM	PHYIqSD	0.322	±	0.001	0.277	±	0.261	0.556	±	0.050
H123#162	SSETSFPHF	SPMFGR	PEYLPAP	0.944	±	0.028	0.277	±	0.191	0.232	±	0.024
1st-F10-0.1-10E	SSEFqFPFF	SPMFNV	PGYIRPS	0.763	±	0.023	0.273	±	0.234	0.555	±	0.034
H123#211	SSELMFPFF	SPMFPL	PQYLVSP	0.340	±	0.009	0.272	±	0.734	1.885	±	0.188
3rd_0.5-4-11B	SSEFFFPYF	SPMFGT	PEYSAAS	0.851	±	0.027	0.270	±	0.421	0.855	±	0.121
3rd_1-4-G-06	SSEFFFPFF	SPMFqN	PTYGTSS	1.044	±	0.032	0.270	±	0.149	0.387	±	0.025
H123#185	SSELMFPYF	SPMFRM	PAYQVQP	0.748	±	0.032	0.269	±	0.044	0.468	±	0.050
H123#7	SSEVqFPFF	SPMFQH	PTYYTSA	1.634	±	0.048	0.269	±	0.237	0.474	±	0.056
H123#147	SSESMFPYF	SPMFqY	PSYRSSH	0.668	±	0.018	0.267	±	0.159	0.694	±	0.091
3rd_1-2-E-11	SSEWqFPFF	SPMFPS	PLYTTRG	1.142	±	0.060	0.267	±	0.167	0.471	±	0.050
1101-119	SSELLFPLF	SPMFGM	PHYQPRE	0.334	±	0.017	0.260	±	1.334	1.793	±	0.305
3rd_1-2-F-08	SSELLFPMF	SPMFHA	PSYLPQE	0.372	±	0.007	0.258	±	0.606	1.484	±	0.145
H123#87	SSEWLFPFF	SPMFPN	PRYRVNH	0.801	±	0.009	0.258	±	0.428	0.975	±	0.069
1101-38	SSEMTFPSF	SPMFRq	PFYYPTE	0.359	±	0.004	0.251	±	0.793	0.988	±	0.142
H123#299	SSELLFPFF	SPMFGK	PQYELSH	0.942	±	0.061	0.250	±	0.141	0.249	±	0.027
H123#410	SSELTFPYF	SPMFGL	PAYYTNE	1.536	±	0.069	0.250	±	0.089	0.445	±	0.037
1101-93	SSENMFPYF	SPMFGI	PEYTqPK	0.432	±	0.003	0.249	±	0.244	0.815	±	0.129
H123#362	SSELLFPYF	SPMFqS	PAYAESG	0.267	±	0.006	0.249	±	1.673	1.656	±	0.157
H123#282	SSETTFPYF	SPMFqL	PAYAASP	1.064	±	0.028	0.248	±	0.122	0.153	±	0.014
1026B127	SSELMFPMF	SPMFqS	PSYNSSH	0.607	±	0.004	0.247	±	0.604	0.602	±	0.048
H123#33	SSELNFPNF	SPMFSq	PSYTPRG	0.342	±	0.005	0.245	±	0.891	1.370	±	0.110
3rd-1-E2	SSEWqFPYF	SPMFSL	PSYYPNS	3.288	±	0.201	0.243	±	0.087	0.154	±	0.014
1101-94	SSEFqFPYF	SPMFST	PTYTTRG	0.394	±	0.001	0.241	±	0.526	0.872	±	0.104
H123#104	SSELSFPFF	SPMFAq	PRYYPSE	1.697	±	0.097	0.241	±	0.055	0.388	±	0.059
H123#354	SSELqFPFF	SPMFAK	PSYHPSH	0.261	±	0.001	0.241	±	1.253	1.037	±	0.074
1101-184	SSELNFPMF	SPMFNI	PAYYPNY	0.563	±	0.022	0.230	±	0.764	0.781	±	0.143
H123#327	SSELNFPSF	SPMFNq	PTYLSSG	0.280	±	0.013	0.230	±	1.256	1.124	±	0.110
3rd_0.5-1-11F	SSELAFPFF	SPMFSL	PSYLKTG	0.520	±	0.005	0.223	±	1.064	0.697	±	0.102
3rd_1-0-C-9	SSEPKFPYF	SPMFQH	PAYKTNG	0.622	±	0.036	0.220	±	0.371	0.508	±	0.078
H123#309	SSELqFPFF	SPMFPI	PFYHTSY	0.633	±	0.008	0.219	±	0.406	0.577	±	0.055
1101-41	SSELqFPHF	SPMFNY	PTYRSFG	0.378	±	0.033	0.216	±	1.203	1.233	±	0.162
H123#455	SSELqFPAF	SPMFSL	PTYRPAG	0.160	±	0.009	0.215	±	0.691	1.365	±	0.224
H123#470	SSETTFPSF	SPMFNq	PHYFSSQ	0.394	±	0.009	0.214	±	0.695	0.570	±	0.100
1026B91	SSELLFPLF	SPMFRq	PTYLPTS	0.271	±	0.024	0.212	±	1.537	2.704	±	0.358
H123#218	SSEFTFPFF	SPMFQI	PEYRSSM	0.407	±	0.007	0.211	±	0.391	0.943	±	0.104
H123#184	SSELGFPSF	SPMFHq	PTYYPSq	0.340	±	0.007	0.211	±	0.241	1.206	±	0.121
3rd-0.5-G7	SSEVTFPLF	SPMFNT	PTYMPSV	2.411	±	0.261	0.209	±	0.104	0.159	±	0.020
1026B36	SSEFqFPFF	SPMFQQ	PSYMPGY	0.422	±	0.027	0.205	±	0.813	1.205	±	0.128
3rd-1-E4	SSELVFPFF	SPMFGT	PLYWPSH	2.622	±	0.059	0.202	±	0.081	0.171	±	0.013
H123#67	SSEVTFPAF	SPMFGA	PTYTPGV	1.344	±	0.035	0.202	±	0.195	0.317	±	0.028
H123#379	SSEIqFPFF	SPMFSR	PQYIPRH	0.117	±	0.004	0.201	±	4.918	1.355	±	0.108
H123#328	SSEPMFPAF	SPMFFS	PSYYPGP	0.987	±	0.041	0.199	±	0.082	0.225	±	0.019
H123#113	SSEDNFPMF	SPMFPq	PTYHPTA	0.511	±	0.042	0.196	±	0.352	0.480	±	0.138
1st-F10-0.1-5G	SSEMTFPFF	SPMFNQ	PVYFMSY	1.608	±	0.060	0.193	±	0.254	0.235	±	0.025

3rd-0.5-A11	SSEFLFPAF	SPMFqQ	PEYRPSP	0.551	±	0.021	0.189	±	0.473	0.740	±	0.088
H123#320	SSETqFPLF	SPMFSA	PTYLPSH	0.349	±	0.013	0.188	±	0.340	0.537	±	0.049
3rd_1-0.0625-D-3	SSEITFPFF	SPMFqN	PGYQLTT	0.676	±	0.024	0.183	±	0.481	0.322	±	0.037
1026B20	SSEIIFPFF	SPMFGI	PTYSATF	1.069	±	0.090	0.179	±	0.309	0.605	±	0.072
3rd_0.5-0.5-2F	SSELLFPFF	SPMFNL	PRYLqYQ	0.448	±	0.026	0.176	±	0.198	0.513	±	0.047
1026R79	SSELqFPAF	SPMFPW	PSYQPSP	1.357	±	0.020	0.174	±	0.197	0.373	±	0.030
3rd_1-4-H-05	SSELLFPFF	SPMFSV	PNYNqTA	0.471	±	0.018	0.174	±	0.556	0.925	±	0.139
H123#90	SSEVNFPMF	SPMFAH	PQYSAqG	0.687	±	0.004	0.174	±	0.269	0.397	±	0.029
3rd_1-0.0625-F-3	SSEITFPFF	SPMFGq	PAYHLTS	3.417	±	0.183	0.173	±	0.079	0.189	±	0.025
2nd-0.5-0.0625-F-9	SSEFFFPFF	SPMFDR	PSYLKDG	1.304	±	0.069	0.172	±	0.327	0.206	±	0.025
H123#293	SSELMFPFF	SPMFHq	PRYWQSG	0.501	±	0.011	0.171	±	0.165	0.526	±	0.047
0-A6	SSEFLFPFF	SPMFNI	PQYKEHL	1.311	±	0.047	0.169	±	0.324	0.221	±	0.027
3RD-1-3B	SSEMTFPYF	SPMFqT	PGYLPSS	0.435	±	0.069	0.169	±	0.377	0.909	±	0.167
3rd_0.5-0.25-11H	SSEVqFPHF	SPMFET	PEYYPTD	0.595	±	0.026	0.169	±	0.249	0.381	±	0.036
0.125-H1	SSELLFPqF	SPMFST	PTYLPSH	0.542	±	0.010	0.166	±	0.832	0.693	±	0.075
H123#201	SSEHTFPYF	SPMFqN	PGYKPSN	0.701	±	0.014	0.162	±	0.052	0.487	±	0.049
H123#19	SSEWLFPFF	SPMFHL	PQYNRSA	0.864	±	0.034	0.161	±	0.184	0.240	±	0.029
3rd_0.5-0-3H	SSELqFPNF	SPMFQL	PTYLPTS	0.328	±	0.003	0.160	±	0.732	1.644	±	0.115
H123#109	SSERPFPAF	SPMFNY	PSYLPGM	1.684	±	0.086	0.159	±	0.080	0.117	±	0.018
H123#166	SSEVHFPNF	SPMFqH	PSYSPGP	0.519	±	0.030	0.158	±	0.287	0.725	±	0.087
H123#226	SSELSFPSF	SPMFNq	PSYVPRN	0.403	±	0.001	0.154	±	0.242	0.552	±	0.048
1101-42	SSEWLFPFF	SPMFHL	PDYRPTA	0.385	±	0.034	0.148	±	0.635	1.138	±	0.152
H123#79	SSELLFPMF	SPMFDT	PTYFAGP	1.145	±	0.068	0.147	±	0.081	0.439	±	0.050
H123#310	SSELMFPSF	SPMFGq	PRYTLQG	0.531	±	0.011	0.145	±	0.633	0.332	±	0.074
1101-34	SSEWVFPFF	SPMFHq	PTYNPSN	0.384	±	0.022	0.145	±	0.757	0.659	±	0.094
3rd-1-F1	SSELLFPFF	SPMFGI	PAYTRTH	2.021	±	0.170	0.144	±	0.148	0.205	±	0.020
3rd_1-0-A-8	SSEIVFPLF	SPMFqH	PSYQARG	0.587	±	0.003	0.138	±	0.866	1.163	±	0.147
H123#397	SSELTFPYF	SPMFTR	PTYHASS	0.140	±	0.001	0.136	±	1.989	1.686	±	0.126
3rd_0.5-0.5-5H	SSELLFPYF	SPMFqH	PAYKIEP	1.556	±	0.065	0.135	±	0.082	0.272	±	0.021
H123#346	SSEVTFPSF	SPMFGq	PRYRPVP	0.326	±	0.038	0.132	±	1.047	0.665	±	0.092
3RD-1-1C	SSELTFPLF	SPMFPq	PTYNSFN	0.852	±	0.045	0.130	±	0.317	0.368	±	0.044
H123#254	SSEYLFPMF	SPMFqL	PAYQPTA	0.757	±	0.012	0.120	±	0.434	0.374	±	0.033
H123#426	SSEVLFPYF	SPMFGL	PAYSPNN	1.299	±	0.107	0.119	±	0.143	0.159	±	0.019
1ST-8G	SSEYLFPLF	SPMFAF	PTYVPTD	0.402	±	0.035	0.113	±	0.413	0.709	±	0.088
H123#71	SSEPTFPTF	SPMFqN	PTYLPTE	0.560	±	0.008	0.112	±	0.381	0.356	±	0.027
1101-15	SSEMTFPMF	SPMFqI	PSYAPRG	0.347	±	0.002	0.111	±	0.972	1.730	±	0.209
1101-131	SSELLFPLF	SPMFQL	PSYKVSQ	0.348	±	0.007	0.105	±	0.366	1.572	±	0.315
2D-0.5-8A	SSELTFPHF	SPMFRq	PTYRPQP	1.038	±	0.010	0.105	±	0.625	0.382	±	0.043
0-F2	SSEVLFPLF	SPMFST	PSYHPTN	0.883	±	0.023	0.099	±	0.490	0.292	±	0.032
3rd_1-4-C-06	SSELqFPGF	SPMFPQ	PKYYVTG	0.271	±	0.033	0.097	±	1.609	0.684	±	0.103
H123#329	SSEFTFPMF	SPMFPK	PTYqAGM	0.664	±	0.031	0.095	±	0.650	0.897	±	0.076
H123#364	SSEARFPAF	SPMFNq	PTYHTSH	0.249	±	0.002	0.089	±	0.607	0.598	±	0.043
H123#95	SSESEFPFF	SPMFGI	PTYLPGA	0.972	±	0.053	0.087	±	0.471	0.517	±	0.040
3rd_1-0-E-9	SSEFHFPFF	SPMFqK	PAYDTDA	1.056	±	0.012	0.087	±	0.430	0.162	±	0.018
3rd_1-4-G-02	SSEISFPYF	SPMFqN	PAYYINQ	1.197	±	0.032	0.086	±	0.229	0.203	±	0.019
3rd_1-0.0625-F-2	SSEMTFPNF	SPMFNW	PVYNASA	1.554	±	0.024	0.083	±	0.104	0.117	±	0.013
1101-64	SSELqFPMF	SPMFHL	PAYLPRD	0.523	±	0.014	0.082	±	0.342	0.865	±	0.161
H123#9	SSEATFPFF	SPMFGQ	PEYIPTH	0.555	±	0.040	0.082	±	0.983	0.756	±	0.068
H123#21	SSELNFPSF	SPMFAT	PTYRPGA	1.678	±	0.028	0.080	±	0.204	0.115	±	0.007

H123#350	SSEVTFPLF	SPMFGF	PTYYFqP	0.500	±	0.013	0.078	±	0.744	0.803	±	0.125
H123#125	SSEVNFPMF	SPMFNV	PQYSPAP	2.436	±	0.133	0.077	±	0.028	0.129	±	0.015
3rd_0.5-0-4B	SSEINFPYF	SPMFqI	PGYLTPG	0.572	±	0.026	0.076	±	0.376	1.155	±	0.119
H123#340	SSELLFPYF	SPMFqR	PQYSWSH	0.733	±	0.014	0.076	±	0.730	0.556	±	0.042
H123#156	SSESTFPQF	SPMFPN	PTYYPSH	1.299	±	0.125	0.073	±	0.243	0.462	±	0.064
3rd_0.5-0.25-12G	SSELLFPFF	SPMFNS	PNYHDTR	0.856	±	0.109	0.071	±	0.151	0.546	±	0.091
1101-67	SSEAFFPFF	SPMFqH	PKYHVSP	0.399	±	0.004	0.064	±	0.881	0.509	±	0.086
3rd_1-0.0625-F-1	SSEMSFPLF	SPMFDH	PSYLLPK	0.764	±	0.026	0.063	±	0.809	0.262	±	0.032
H123#473	SSEMqFPFF	SPMFLS	PTYIPTA	0.533	±	0.016	0.063	±	0.602	0.542	±	0.088
H123#27	SSEQEFPYF	SPMFYL	PTYVPSS	0.996	±	0.047	0.058	±	0.513	0.229	±	0.017
H123#304	SSELTFPYF	SPMFQM	PAYAPTF	0.873	±	0.100	0.057	±	0.366	0.676	±	0.097
3RD-0.25-8B	SSELLFPqF	SPMFNL	PTYFSGP	1.545	±	0.021	0.055	±	0.390	0.231	±	0.025
H123#331	SSEMFFPYF	SPMFDM	PEYIPNq	0.244	±	0.012	0.055	±	1.983	0.874	±	0.095
3RD-1-4C	SSEFLFPLF	SPMFGH	PMYTPSY	0.124	±	0.008	0.054	±	2.121	2.748	±	0.329
1101-44	SSEFqFPHF	SPMFQI	PAYLPSQ	0.575	±	0.031	0.051	±	0.731	0.491	±	0.070
H123#283	SSEFNFPqF	SPMFGM	PTYQPRS	0.495	±	0.005	0.050	±	0.462	0.284	±	0.024
3rd_0.5-0.5-1G	SSELLFPLF	SPMFQq	PAYTPSM	0.569	±	0.019	0.050	±	0.126	0.592	±	0.056
H123#316	SSELqFPAF	SPMFSH	PGYYPSA	0.564	±	0.021	0.044	±	0.219	0.372	±	0.035
1101-58	SSEFLFPYF	SPMFPY	PTYHQRD	0.916	±	0.010	0.044	±	0.079	0.254	±	0.039
3rd_0.5-4-07H	SSEWqFPFF	SPMFGL	PEYMYGR	0.539	±	0.015	0.043	±	1.335	0.640	±	0.111
H123#48	SSEVTFPYF	SPMFqL	PqYRQDF	0.362	±	0.008	0.042	±	1.679	2.251	±	0.275
H123#223	SSEVNFPLF	SPMFqT	PTYYPRS	1.001	±	0.145	0.040	±	0.312	0.304	±	0.052
H123#130	SSEINFPAF	SPMFST	PRYLVNG	0.884	±	0.065	0.039	±	0.356	0.632	±	0.077
H123#249	SSEMTFPFF	SPMFHH	PGYMRTA	0.639	±	0.003	0.038	±	0.358	0.667	±	0.059
1026B54	SSELLFPMF	SPMFSH	PGYMPqE	0.400	±	0.049	0.031	±	1.521	1.893	±	0.316
H123#267	SSEqSFPFF	SPMFGT	PAYRPTS	0.537	±	0.024	0.028	±	0.450	0.294	±	0.028
H123#356	SSEYTFPMF	SPMFGq	PSYIMPS	0.367	±	0.005	0.027	±	0.676	0.760	±	0.063
H123#26	SSEYMFPLF	SPMFPq	PSYRVSS	0.446	±	0.024	0.026	±	0.967	0.389	±	0.029
H123#209	SSEWqFPYF	SPMFQR	PSYFPSP	0.545	±	0.079	0.026	±	0.288	1.431	±	0.253
1101-113	SSELAFPYF	SPMFqH	PAYYVSG	0.308	±	0.017	0.025	±	1.034	1.397	±	0.233
H123#402	SSEFTFPYF	SPMFqL	PTYQVAP	0.848	±	0.066	0.022	±	0.536	0.514	±	0.057
1026R49	SSELLFPYF	SPMFGq	PSYTARQ	0.622	±	0.050	0.022	±	0.732	1.016	±	0.107
2nd-0.5-F10-0.1-12F	SSEMVFPHF	SPMFQV	PqYIPTG	0.481	±	0.022	0.022	±	0.438	1.696	±	0.246
2D-0.5-12E	SSETLFPMF	SPMFqF	PVYYTSG	0.162	±	0.004	0.013	±	1.666	1.190	±	0.116
H123#5	SSELNFPYF	SPMFML	PTYLSTG	0.519	±	0.031	0.009	±	0.606	0.375	±	0.032
3rd_1-1-A-3	SSEWqFPSF	SPMFSV	PHYLLPL	1.148	±	0.026	0.008	±	0.430	0.175	±	0.019
3rd_1-0.0625-C-6	SSELqFPYF	SPMFSI	PQYTPTQ	1.026	±	0.008	0.006	±	0.217	0.297	±	0.040
H123#22	SSELTFPYF	SPMFTL	PSYNPNP	0.854	±	0.022	0.005	±	0.699	0.505	±	0.039
2nd-0.5-0.0625-D-11	SSEYTFPYF	SPMFqT	PTYTTSE	1.956	±	0.052	0.003	±	0.347	0.135	±	0.015
1026B2	SSEIqFPFF	SPMFPA	PTYVPRS	0.341	±	0.013	-0.001	±	0.648	1.227	±	0.117
H123#56	SSELqFPFF	SPMFNL	PHYMNPM	1.202	±	0.057	-0.003	±	0.418	0.701	±	0.077
3rd_0.5-0-1D	SSELYFPFF	SPMFPR	PGYKPSA	0.315	±	0.005	-0.005	±	0.993	1.207	±	0.114
1101-35	SSELTFPHF	SPMFSL	PQYVEGS	0.692	±	0.031	-0.010	±	0.387	0.652	±	0.061
H123#256	SSEIMFPFF	SPMFPq	PTYLASN	0.529	±	0.009	-0.010	±	0.765	0.874	±	0.075
H123#242	SSEIAFPYF	SPMFGT	PAYTTSP	0.964	±	0.167	-0.017	±	0.087	0.161	±	0.031
3RD-1-1B	SSELqFPYF	SPMFPR	PTYYPIH	1.693	±	0.069	-0.018	±	0.172	0.238	±	0.031
3rd_0.5-2-2C	SSEAVFPFF	SPMFNQ	PTYTPNG	0.329	±	0.003	-0.019	±	2.607	0.748	±	0.139
H123#395	SSEMNFPFF	SPMFGQ	PTYGLIN	1.593	±	0.087	-0.022	±	0.228	0.080	±	0.007
H123#398	SSESDFPYF	SPMFNA	PAYYTTG	0.564	±	0.069	-0.032	±	0.879	0.596	±	0.086

H123#165	SSELTFPRF	SPMFPT	PSYYPDS	0.944	±	0.011	-0.034	±	0.140	0.371	±	0.037
H123#143	SSELNFPYF	SPMFMQ	PSYLVGA	0.783	±	0.046	-0.035	±	0.177	0.334	±	0.038
3rd_0.5-0-3G	SSEFqFPHF	SPMFSS	PTYLPSL	0.429	±	0.039	-0.036	±	1.082	1.252	±	0.171
3rd_1-0.0625-F-6	SSEFLFPLF	SPMFAT	PTYTPTY	0.835	±	0.017	-0.039	±	0.715	0.503	±	0.056
H123#11	SSELTFPMF	SPMFNN	PTYLLNG	0.797	±	0.039	-0.040	±	0.523	0.527	±	0.039
3rd_1-2-B-07	SSEVVFPFF	SPMFqI	PAYSKAS	1.106	±	0.035	-0.042	±	0.230	0.164	±	0.016
H123#73	SSELTFPAF	SPMFPT	PQYRPTH	0.328	±	0.015	-0.046	±	1.617	1.511	±	0.221
3rd_0.5-0-1A	SSEFLFPHF	SPMFYM	PSYVTKG	0.330	±	0.000	-0.051	±	1.006	1.021	±	0.115
H123#158	SSELVFPLF	SPMFqR	PSYVPSG	0.384	±	0.005	-0.056	±	0.563	1.482	±	0.150
H123#436	SSESTFPMF	SPMFqL	PRYAPSP	0.274	±	0.015	-0.057	±	0.865	0.900	±	0.095
H123#366	SSEMMFPNF	SPMFqT	PTYKPTN	1.072	±	0.017	-0.059	±	0.404	0.181	±	0.013
0.25-E4	SSELqFPMF	SPMFPR	PEYTASP	0.811	±	0.045	-0.061	±	0.540	0.418	±	0.058
H123#406	SSEVqFPFF	SPMFQT	PEYRASP	0.192	±	0.003	-0.063	±	0.835	1.813	±	0.147
1011-401	SSELqFPFF	SPMFNH	PGYHVST	0.595	±	0.022	-0.065	±	0.693	0.497	±	0.046
H123#339	SSEWQFPFF	SPMFQA	PTYSPGE	0.963	±	0.037	-0.066	±	0.477	0.265	±	0.022
H123#174	SSEIMFPFF	SPMFqR	PAYWASP	0.391	±	0.015	-0.069	±	0.643	0.900	±	0.095
H123#302	SSEIMFPAF	SPMFGq	PSYRMTV	0.425	±	0.006	-0.081	±	0.079	0.333	±	0.030
H123#297	SSEVNFPMF	SPMFHI	PHYFNHA	1.226	±	0.059	-0.085	±	0.088	0.147	±	0.014
H123#6	SSEVTFPLF	SPMFQM	PFYLPSS	1.426	±	0.047	-0.109	±	0.350	0.528	±	0.039
H123#300	SSELSFPqF	SPMFSY	PAYTVSP	0.427	±	0.012	-0.110	±	0.782	0.320	±	0.029
3rd_0.5-0.25-10B	SSELqFPLF	SPMFSL	PSYSGSP	0.648	±	0.070	-0.114	±	0.318	0.080	±	0.010
0.25-E6	SSENAFPSF	SPMFNF	PTYYATH	0.625	±	0.034	-0.131	±	0.729	0.509	±	0.062
3rd_1-2-C-10	SSELSFPFF	SPMFqT	PSYTLRG	0.627	±	0.111	-0.137	±	0.554	0.293	±	0.057
H123#43	SSEEYFPNF	SPMFqL	PTYLTKG	0.383	±	0.005	-0.142	±	1.251	0.654	±	0.042
1026B98	SSELHFPVF	SPMFNT	PTYFTTq	0.396	±	0.016	-0.144	±	0.266	0.792	±	0.080
H123#384	SSEIVFPFF	SPMFST	PSYATKG	0.368	±	0.020	-0.150	±	1.827	1.746	±	0.158
1026B79	SSEFLFPLF	SPMFqR	PGYLPRD	0.385	±	0.014	-0.152	±	0.941	1.651	±	0.237
H123#222	SSEPTFPFF	SPMFHS	PTYAKAG	0.923	±	0.034	-0.167	±	0.371	0.183	±	0.017
H123#181	SSEFLFPSF	SPMFSQ	PSYRPSE	0.694	±	0.022	-0.175	±	0.220	0.666	±	0.090
H123#17	SSELIFPFF	SPMFLN	PTYHINS	0.615	±	0.010	-0.183	±	0.721	0.582	±	0.042
1101-16	SSEWqFPFF	SPMFNT	PEYVPRD	0.381	±	0.009	-0.196	±	0.871	0.910	±	0.108
3RD-1-5H	SSEFqFPSF	SPMFHV	PDYLPSS	0.201	±	0.009	-0.202	±	1.298	1.641	±	0.164
H123#447	SSELLFPFF	SPMFqP	PLYTTNG	0.449	±	0.042	-0.216	±	0.656	0.674	±	0.085
3rd_1-1-C-3	SSELqFPHF	SPMFTF	PTYTPSL	0.836	±	0.039	-0.221	±	0.489	0.623	±	0.087
H123#371	SSEASFPAF	SPMFPR	PTYYNDS	0.739	±	0.013	-0.227	±	0.883	0.194	±	0.015
H123#389	SSELqFPSF	SPMFQF	PTYNVGS	0.252	±	0.005	-0.229	±	1.890	0.620	±	0.047
3rd_0.5-0.0625-8B	SSELTFPFF	SPMFRq	PTYNTTY	0.317	±	0.008	-0.229	±	0.746	0.940	±	0.080
H123#445	SSELqFPFF	SPMFRN	PTYTPSM	0.432	±	0.039	-0.233	±	0.742	1.011	±	0.134
0-E4	SSEFLFPqF	SPMFNT	PEYFLGS	0.924	±	0.042	-0.238	±	0.844	0.210	±	0.025
H123#287	SSELLFPLF	SPMFGQ	PRYRASA	0.518	±	0.007	-0.241	±	0.144	0.779	±	0.066
H123#345	SSEITFPNF	SPMFqL	PTYLGSA	0.258	±	0.026	-0.250	±	1.907	1.049	±	0.131
3rd_0.5-0.125-1B	SSELqFPFF	SPMFHM	PLYQTRA	0.405	±	0.009	-0.253	±	0.662	0.712	±	0.152
H123#358	SSEWqFPLF	SPMFRL	PVYMPDS	0.337	±	0.011	-0.256	±	0.727	0.417	±	0.034
H123#373	SSELSFPQF	SPMFNA	PTYLPSL	1.662	±	0.054	-0.257	±	0.559	0.121	±	0.010
H123#323	SSELqFPLF	SPMFNV	PSYMRTD	0.564	±	0.028	-0.269	±	1.043	0.241	±	0.022
3rd_1-0.0625-A-6	SSELTFPLF	SPMFHq	PQYIANG	0.611	±	0.006	-0.269	±	0.880	1.187	±	0.137
3rd_1-0.0625-G-2	SSELqFPNF	SPMFPL	PSYNPRG	1.790	±	0.045	-0.272	±	0.376	0.102	±	0.011
H123#380	SSELNFPqF	SPMFAY	PFYFVNP	0.257	±	0.013	-0.274	±	2.744	0.566	±	0.052
H123#318	SSELqFPSF	SPMFHS	PSYNVDG	0.311	±	0.039	-0.276	±	1.978	0.595	±	0.090

H123#361	SSETQFPFF	SPMFqV	PTYVPNS	1.005	±	0.049	-0.277	±	0.627	0.190	±	0.017
3RD-0.25-7C	SSEIqFPYF	SPMFNT	PSYVPRE	0.602	±	0.016	-0.287	±	1.079	0.705	±	0.085
3rd_0.5-0.0625-07A	SSEFqFPYF	SPMFQL	PSYQSSP	0.537	±	0.038	-0.289	±	1.608	0.717	±	0.125
H123#92	SSELFFPHF	SPMFQI	PAYLVTD	0.946	±	0.105	-0.292	±	0.248	0.328	±	0.042
H123#403	SSEALFPIF	SPMFGT	PTYRPTG	0.146	±	0.003	-0.293	±	3.154	1.020	±	0.075
3rd_1-2-E-09	SSELqFPLF	SPMFHK	PTYLPGS	0.898	±	0.082	-0.296	±	0.341	0.236	±	0.030
3rd_0.5-4-11H	SSEWLFPLF	SPMFQA	PLYYPTS	0.700	±	0.021	-0.308	±	0.762	0.535	±	0.074
1101-70	SSELTFPYF	SPMFHS	PTYASSP	0.759	±	0.100	-0.316	±	0.452	0.389	±	0.081
H123#88	SSELLFPFF	SPMFNT	PGYATSN	0.333	±	0.005	-0.322	±	0.721	1.694	±	0.179
1026B50	SSEFLFPMF	SPMFqN	PQYYVDN	0.355	±	0.016	-0.328	±	1.290	1.618	±	0.166
1101-167	SSELAFPYF	SPMFAq	PTYYQSS	0.367	±	0.002	-0.335	±	0.543	0.743	±	0.150
3rd_0.5-0.0625-09F	SSELTFPFF	SPMFYY	PTYqATK	0.318	±	0.018	-0.341	±	1.279	0.596	±	0.075
H123#396	SSEWVFPHF	SPMFGI	PSYVTNN	0.254	±	0.003	-0.343	±	1.636	0.507	±	0.036
1026B84	SSEMLFPFF	SPMFDA	PTYYQVG	0.219	±	0.000	-0.350	±	1.938	3.020	±	0.292
H123#387	SSEHIFPHF	SPMFGq	PTYYVSY	0.135	±	0.004	-0.354	±	2.627	1.351	±	0.112
H123#306	SSEYMFPNF	SPMFAF	PEYLPTN	0.914	±	0.060	-0.373	±	0.207	0.276	±	0.030
H123#248	SSEATFPFF	SPMFNT	PHYRFSA	0.998	±	0.021	-0.400	±	0.215	0.189	±	0.017
3rd_1-2-F-10	SSEILFPMF	SPMFDq	PQYNPSH	0.380	±	0.058	-0.400	±	1.310	0.755	±	0.133
3rd_0.5-0.0625-08A	SSERqFPFF	SPMFPP	PTYYPTq	0.306	±	0.001	-0.408	±	0.632	0.826	±	0.099
2ND-1-C1	SSEVLFPLF	SPMFGq	PYYFPSE	0.534	±	0.026	-0.413	±	0.805	1.027	±	0.127
1101-101	SSELqFPHF	SPMFSI	PNYIRPS	0.341	±	0.032	-0.420	±	1.557	0.795	±	0.183
3rd_1-0.0625-E-3	SSEqNFPFF	SPMFQN	PTYRSSA	1.116	±	0.063	-0.429	±	0.597	0.182	±	0.022
3RD-1-4E	SSELqFPLF	SPMFGV	PLYYPME	0.248	±	0.025	-0.435	±	1.053	1.113	±	0.149
H123#230	SSELQFPVF	SPMFRQ	PTYIPSL	0.558	±	0.011	-0.440	±	0.227	0.294	±	0.028
H123#41	SSETAFPHF	SPMFqY	PTYLLSH	0.392	±	0.031	-0.447	±	1.029	0.473	±	0.049
H123#247	SSELWFPFF	SPMFqV	PTYGPSA	0.436	±	0.017	-0.457	±	0.377	0.798	±	0.074
H123#49	SSEWqFPMF	SPMFAL	PGYTPDS	0.559	±	0.037	-0.462	±	0.795	0.307	±	0.027
0.5-B2	SSEIqFPLF	SPMFPH	PTYQPDA	0.189	±	0.025	-0.463	±	1.768	1.450	±	0.228
H123#342	SSEIGFPFF	SPMFQI	PTYLRYD	0.557	±	0.040	-0.471	±	1.388	0.312	±	0.032
1011-1	SSELqFPFF	SPMFTA	PEYNKSG	0.449	±	0.017	-0.474	±	0.466	0.701	±	0.109
1st-F10-0.1-4C	SSEqWFPVF	SPMFVN	PSYSPNP	0.573	±	0.010	-0.485	±	0.191	0.270	±	0.014
H123#435	SSEVNFPFF	SPMFTq	PTYMNTR	0.254	±	0.006	-0.486	±	1.150	0.421	±	0.037
H123#357	SSELqFPYF	SPMFGL	PQYLVNA	0.249	±	0.003	-0.489	±	1.909	0.943	±	0.075
3rd_0.5-0.0625-07H	SSELqFPMF	SPMFGI	PTYLRGT	0.344	±	0.002	-0.500	±	0.920	1.445	±	0.091
3rd_1-1-F-2	SSELqFPSF	SPMFSN	PSYVPSH	0.689	±	0.005	-0.513	±	0.893	0.452	±	0.054
3RD-1-1G	SSELqFPMF	SPMFGL	PSYLRGA	0.199	±	0.020	-0.518	±	1.720	2.328	±	0.321
H123#392	SSELTFPFF	SPMFqN	PDYqPSS	0.120	±	0.002	-0.520	±	5.355	5.175	±	0.386
2D-0.5-12A	SSELMFPAF	SPMFWS	PTYTTSP	0.543	±	0.004	-0.560	±	1.055	0.684	±	0.078
3rd_1-0.0625-F-5	SSEANFPqF	SPMFPI	PTYFqNG	0.626	±	0.003	-0.573	±	0.199	0.310	±	0.034
H123#441	SSEVqFPSF	SPMFKL	PTYIPTA	0.261	±	0.026	-0.574	±	1.186	0.984	±	0.133
H123#24	SSEVTFPMF	SPMFGT	PTYTSSA	1.060	±	0.077	-0.582	±	0.519	0.165	±	0.015
H123#385	SSELqFPYF	SPMFQQ	PTYLPGY	0.260	±	0.049	-0.584	±	1.747	1.705	±	0.344
0.5-A2	SSEVNFPMF	SPMFKq	PHYSFQG	0.504	±	0.001	-0.618	±	0.762	0.347	±	0.037
H123#448	SSELWFPHF	SPMFSP	PTYYLqA	0.473	±	0.026	-0.642	±	0.564	0.265	±	0.027
1101-176	SSEFLFPLF	SPMFDI	PEYYPTS	0.277	±	0.001	-0.645	±	1.182	2.077	±	0.394
2nd-0.5-F10-0.1-11H	SSEWQFPIF	SPMFqN	PAYYPIS	0.765	±	0.044	-0.650	±	0.357	0.217	±	0.017
H123#57	SSEIHFPGF	SPMFNA	PEYWKSA	0.283	±	0.001	-0.657	±	0.480	0.494	±	0.026
1026B34	SSELqFPFF	SPMFRL	PEYLPNG	0.302	±	0.001	-0.659	±	1.713	1.892	±	0.157
H123#378	SSEVSFPMF	SPMFLS	PEYFVPT	0.117	±	0.003	-0.676	±	5.038	1.090	±	0.082

H123#25	SSEWEFPSF	SPMFTA	PTYTTRG	0.878	±	0.068	-0.679	±	0.446	0.292	±	0.028
H123#359	SSEATFPFF	SPMFGN	PRYRDTT	0.227	±	0.001	-0.680	±	0.917	0.620	±	0.045
H123#72	SSELqFPLF	SPMFDL	PTYTPTY	0.314	±	0.004	-0.681	±	1.730	0.858	±	0.078
H123#399	SSEVGFPFF	SPMFqV	PEYFAFN	0.442	±	0.004	-0.698	±	1.951	0.671	±	0.052
H123#374	SSELMFPMF	SPMFqQ	PAYASRG	0.132	±	0.004	-0.705	±	2.975	1.625	±	0.124
3rd_0.5-0.125-3C	SSEWTFPFF	SPMFAS	PGYHAGS	0.379	±	0.015	-0.706	±	1.353	0.435	±	0.069
H123#432	SSEWNFPYF	SPMFqL	PRYSVGP	0.618	±	0.166	-0.728	±	1.010	0.373	±	0.106
H123#240	SSELqFPFF	SPMFAR	PSYTKAF	0.410	±	0.017	-0.738	±	0.463	0.424	±	0.041
H123#330	SSEWqFPFF	SPMFqL	PLYQPTP	0.370	±	0.003	-0.746	±	0.845	0.535	±	0.041
H123#416	SSELAFPSF	SPMFAL	PRYRPDG	0.415	±	0.028	-0.765	±	1.014	1.028	±	0.109
H123#217	SSEVNFPSF	SPMFRW	PSYSPGG	0.485	±	0.038	-0.780	±	0.781	0.505	±	0.064
H123#376	SSEVTFPMF	SPMFNQ	PSYGqTT	0.671	±	0.005	-0.782	±	1.299	0.227	±	0.017
3rd_1-4-D-03	SSELLFPFF	SPMFqT	PTYKWMq	0.317	±	0.036	-0.805	±	1.168	0.499	±	0.076
H123#338	SSEMTFPFF	SPMFNq	PHYKNNK	0.268	±	0.005	-0.806	±	2.732	0.729	±	0.055
H123#343	SSEISFPSF	SPMFPW	PTYWMSH	0.256	±	0.015	-0.823	±	2.864	0.782	±	0.079
H123#394	SSELLFPMF	SPMFSq	PQYSPGP	0.249	±	0.022	-0.844	±	1.899	1.532	±	0.176
H123#450	SSEYTFPLF	SPMFGE	PTYqATY	0.187	±	0.008	-0.850	±	1.139	0.704	±	0.067
H123#337	SSEVSFPFF	SPMFNM	PVYRSSE	0.380	±	0.018	-0.887	±	1.369	0.361	±	0.031
H123#440	SSEVTFPLF	SPMFqM	PSYTSGF	0.261	±	0.021	-0.966	±	1.230	1.309	±	0.157
H123#355	SSEPQFPMF	SPMFqL	PTYVPWG	0.233	±	0.002	-1.004	±	1.826	0.757	±	0.054
H123#322	SSEWqFPAF	SPMFGT	PHYMTPK	0.340	±	0.002	-1.005	±	1.622	0.410	±	0.030
H123#40	SSETqFPAF	SPMFNM	PSYQPTM	0.558	±	0.017	-1.032	±	0.744	0.267	±	0.017
H123#344	SSEVSFPqF	SPMFQL	PTYWAKG	0.255	±	0.017	-1.041	±	1.115	0.631	±	0.062
1026B49	SSEITFPMF	SPMFSR	PTYLTGS	0.305	±	0.004	-1.044	±	2.294	2.314	±	0.188
H123#369	SSELFFPFF	SPMFST	PTYHATH	0.128	±	0.014	-1.054	±	5.138	3.492	±	0.465
H123#451	SSEMHFPSF	SPMFQH	PTYFITA	0.131	±	0.004	-1.068	±	4.948	0.830	±	0.077
H123#303	SSELqFPFF	SPMFKH	PGYWGHG	0.410	±	0.008	-1.094	±	0.746	0.367	±	0.032
3rd_0.5-0.0625-09H	SSEMqFPYF	SPMFSW	PGYLPRP	0.35	±	0.022	-1.148	±	0.863	0.358	±	0.038
H123#368	SSELqFPFF	SPMFHY	PSYGSSP	0.243	±	0.005	-1.248	±	3.063	0.623	±	0.047
0.125-H3	SSELSFPLF	SPMFSL	PTYLTRA	0.166	±	0.019	-1.298	±	2.638	1.745	±	0.250
H123#367	SSEIHFPFF	SPMFLq	PTYLVAG	0.269	±	0.005	-1.318	±	2.658	1.059	±	0.096
H123#401	SSELAFPFF	SPMFQQ	PSYIAGV	0.294	±	0.004	-1.333	±	2.232	1.342	±	0.114
1026B55	SSELVFPSF	SPMFNT	PTYRPTY	0.247	±	0.013	-1.344	±	2.678	1.799	±	0.177
3rd_0.5-0.25-07A	SSEFqFPMF	SPMFNH	PVYMARG	0.343	±	0.001	-1.350	±	0.558	0.860	±	0.102
H123#375	SSEMTFPFF	SPMFqP	PTYKIGG	0.184	±	0.019	-1.356	±	3.399	1.166	±	0.149
H123#360	SSELQFPSF	SPMFqL	PVYITRG	0.286	±	0.004	-1.498	±	1.765	1.092	±	0.087
H123#382	SSELqFPYF	SPMFPY	PRYVTRG	0.207	±	0.007	-1.566	±	3.030	0.812	±	0.069
H123#400	SSELLFPLF	SPMFSM	PSYLPKG	0.107	±	0.001	-2.082	±	5.115	3.074	±	0.235
3RD-1-1H	SSEFVFPLF	SPMFqL	PTYSPGA	0.168	±	0.005	-2.483	±	2.606	1.729	±	0.185
H123#377	SSELFFPMF	SPMFAA	PAYFPTF	0.118	±	0.008	-2.882	±	5.628	1.202	±	0.119
H123#408	SSEPTFPHF	SPMFqH	PSYTTWG	0.209	±	0.007	-3.656	±	7.932	1.009	±	0.083

Supplementary Table S6. Data associated with Figure 6c for the purified scFv variants selected from the F10-CDRH123 library. The 1st column from left shows the name assigned to the scFv variants; the 2nd~4th columns show respectively the sequences of CDR-H1~H3; the 5th and 6th columns shows IC_{50} against CA/09 H1N1 virus infection and EC_{50} binding to CA/09 H1 HA respectively. At least three independent measurements were carried out to derive each averaged measurement and standard deviation for each of the scFv variants.

scFv	CDRH1	CDRH2	CDRH3	IC_{50} against CA/09 H1N1 (ng/ml)			EC_{50} against CA/09 H1 HA (ng/ml)		
F10	SSEVTFSSF	SPMFGT	PSYICSGGTC	202.71	±	6.30	22.54	±	2.06
H123#437	SSEFMFPAF	SPMFDH	PTYLPSG	130.54	±	10.97	18.06	±	4.06
1101-178	SSELLFPYF	SPMFNQ	PTYSTKG	163.66	±	14.09	20.87	±	2.00
3rd_0.5_4_09H	SSELVFPLF	SPMFNK	PTYLPRH	109.50	±	5.43	10.18	±	0.31
3rd_0.5_2_2A	SSELLFPFF	SPMFQS	PSYLPGG	142.72	±	2.55	11.95	±	3.03
1101-181	SSEVTFPMF	SPMFTM	PTYLPSY	204.79	±	11.96	110.99	±	10.39
H123#425	SSEVLFPFF	SPMFQR	PTYAPSH	194.87	±	21.34	136.95	±	6.50
3rd_1-4-B-02	SSELMFPYF	SPMFNQ	PSYKPIG	232.94	±	10.43	14.06	±	2.22
1101-150	SSELFFPYF	SPMFSL	PSYRPSH	135.65	±	1.50	29.97	±	1.05
1026B52	SSELFFPYF	SPMFSL	PSYRPSH	74.41	±	2.73	6.28	±	0.83
1026R66	SSEFMFPYF	SPMFEL	PTYVPSG	106.70	±	3.10	24.51	±	0.89
1026B77	SSEMLFPYF	SPMFAT	PTYRPSG	65.30	±	5.50	9.01	±	2.46
1101-170	SSELGFPYF	SPMFDF	PTYLPGA	170.30	±	4.64	14.75	±	0.09

Supplementary References

1. Ekiert, D.C. et al. Antibody recognition of a highly conserved influenza virus epitope. *Science* **324**, 246-251 (2009).
2. Yu, C.M. et al. Rationalization and design of the complementarity determining region sequences in an antibody-antigen recognition interface. *PLoS One* **7**, e33340 (2012).
3. Stewart, M.J. & Watson, I.D. Standard units for expressing drug concentrations in biological fluids. *Br J Clin Pharmacol* **16**, 3-7 (1983).
4. Sui, J. et al. Structural and functional bases for broad-spectrum neutralization of avian and human influenza A viruses. *Nat Struct Mol Biol* **16**, 265-273 (2009).
